# The Financial Risk Measurement EVaR Based on DTARCH Models

**DOI:** 10.3390/e25081204

**Published:** 2023-08-13

**Authors:** Xiaoqian Liu, Zhenni Tan, Yuehua Wu, Yong Zhou

**Affiliations:** 1 Department of Mathematics and Statistics, York University, Toronto, ON M3J 1P3, Canada; xqliu@yorku.ca (X.L.); ztan43@yorku.ca (Z.T.); 2 Key Laboratory of Advanced Theory and Application in Statistics and Data Science, MOE, and Academy of Statistics and Interdisciplinary Sciences and School of Statistics, East China Normal University, Shanghai 200062, China; yzhou@fem.ecnu.edu.cn

**Keywords:** expectile-based VaR (EVaR), double-threshold autoregressive conditional heteroscedastic (DTARCH) models, weighted composite expectile regression (WCER) estimation, risk measurement

## Abstract

The value at risk based on expectile (EVaR) is a very useful method to measure financial risk, especially in measuring extreme financial risk. The double-threshold autoregressive conditional heteroscedastic (DTARCH) model is a valuable tool in assessing the volatility of a financial asset’s return. A significant characteristic of DTARCH models is that their conditional mean and conditional variance functions are both piecewise linear, involving double thresholds. This paper proposes the weighted composite expectile regression (WCER) estimation of the DTARCH model based on expectile regression theory. Therefore, we can use EVaR to predict extreme financial risk, especially when the conditional mean and the conditional variance of asset returns are nonlinear. Unlike the existing papers on DTARCH models, we do not assume that the threshold and delay parameters are known. Using simulation studies, it has been demonstrated that the proposed WCER estimation exhibits adequate and promising performance in finite samples. Finally, the proposed approach is used to analyze the daily Hang Seng Index (HSI) and the Standard & Poor’s 500 Index (SPI).

## 1. Introduction

Scientifically and accurately measuring financial risk is the core part of the financial risk management process. Developing efficient statistical methods of financial risk measurement is essential for effectively controlling financial risk. We aim to develop financial risk models that account for extreme events, thereby enhancing the accuracy and efficacy of risk assessments in the field of finance. Due to the increasing complexity, time-variation and randomness of financial markets, nonlinear time series models are used to provide a more reasonable description of the markets’ behaviors or phenomena, the double-threshold autoregressive conditional heteroscedastic (DTARCH) model is one of nonlinear time series models which are designed for this purpose (see [1] for details). A significant characteristic of DTARCH models is that their conditional mean and conditional variance functions both are piecewise linear involving double thresholds. Our investigation will focus on developing an expectile-based value at risk (EVaR) model with a DTARCH structure.

DTARCH models are very useful and flexible in analyzing asymmetric financial time series, making them a subject of considerable attention in recent statistical and econometric papers. Ref. [1] investigated the model identification, estimation and diagnostic checking techniques based on the maximum likelihood principle under the normal assumption of the conditional distribution of the observed data. Ref. [2] investigated robust modeling techniques without a specific form of the conditional distribution, focusing on the $L_{1}$ estimation of DTARCH models and deriving limiting distributions for the proposed estimators. Ref. [3] further studied the parameter estimation of DTARCH models using the weighted composite quantile regression procedure, which includes quantile regression as a special case while significantly improving efficiency and inheriting robustness. Ref. [4] investigated DTARCH models with restrictions on parameters and proposed both unrestricted and restricted weighted composite quantile regression estimation for the model parameters, which can be utilized to construct the likelihood ratio-type test statistic. However, these papers are all based on the known threshold and delay parameters of DTARCH models.

The risk measure EVaR proposed by [5] is based on expectile regression theory. Ref. [6] proposed the concept of expectile. The expectile is a one-to-one mapping relationship with the quantile and has similar properties as the quantile. So, the expectile can be regarded as an estimation of quantile; see [7,8,9,10] for details. Expectile has gained popularity in recent years as a subject of interest. Ref. [11] discovered that similar to quantiles, time-varying expectiles can be estimated using a state space signal extraction algorithm. Ref. [12] proposed a new model based on expectile regression–geoadditive expectile regression model. Ref. [13] proposed regularized expectile regression with smoothly clipped absolute deviation (SCAD) penalty for analyzing heteroscedasticity in high dimensions when the error has finite moments. Ref. [14] considered penalized linear expectile regression using SCAD penalty function. Ref. [15] proposed aggregated expectile regression by exponential weighting. Ref. [16] derived joint weighted Gaussian approximations of the tail empirical expectile and quantile processes. Ref. [17]focused on the semi-parametric estimation of multivariate expectiles for extreme levels of risk. Ref. [18] proposed expectHill estimators, which are used as the basis for estimating tail expectiles and expected shortfall. Ref. [19] built a general theory for the estimation of extreme conditional expectiles in heteroscedastic regression models with heavy-tailed noise. Ref. [20] developed a weighted expectile regression approach for estimating the conditional expectile when covariates are missing at random. Ref. [21] studied the problem of the nonparametric estimation of the expectile regression model for strong mixing functional time series. Ref. [22] considered model averaging for expectile regressions. Ref. [23] exploited the fact that the expectiles of a distribution *F* are in fact the quantiles of another distribution *E* explicitly linked to *F*, in order to construct nonparametric kernel estimators of extreme conditional expectiles. Ref. [24] dealt with the problem of the nonparametric estimation of the functional expectile regression, and so on.

Since EVaR is derived from expectile theory and utilizes a squared loss function as its loss function, it exhibits higher sensitivity to extreme values and is mathematically easier to handle. In addition, EVaR is a weighted average of the lower risk (expected shortfall, i.e., ES) and upper risk in conditions. Currently, several research papers on EVaR have been published, exploring various aspects of its application and properties. For example, Ref. [11] proved that EVaR is a consistent risk measure when the confidence level *p* is less than 0.5. Ref. [25] studied risk measurement EVaR under a variable coefficient model. Ref. [26] proposed a weighted composite expectile regression estimation for autoregressive models. Ref. [27] discussed the financial meaning of EVaR, compared them with VaR and ES, and studied their asymptotic behavior. Ref. [28] considered a new class of conditional dynamic expectile models with partially varying coefficients in assessing the tail risk of asset returns for S&P 500 Index. Ref. [29] proposed a class of semiparametric composite expectile models with varying coefficients. Ref. [30] proposed a semi-parametric model with varying-coefficients to analyze the EVaR under the assumption of α-mixing. Ref. [31] forecasted the expectile-based risk measures by using the expected-based procedures. Ref. [32] provided a basis for inference on extreme expectiles and expectile-based marginal expected shortfall in a general β-mixing context that encompasses ARMA and GARCH models with heavy-tailed innovations. Ref. [33] developed a single-index approach for modeling the expectile-based value at risk. Ref. [34] studied the estimation of extremal conditional expectile based on quantile regression and expectile regression models. Considering the advantages of EVaR, we will propose the estimation of the DTARCH model based on expectile regression theory. Unlike the existing papers on the DTARCH model, we do not assume that the threshold and delay parameters of DTARCH models are known.

The rest of the paper is organized as follows. Section 2 investigates the estimation problem of DTARCH models based on expectile regression theory. We propose WCER estimation of DTARCH models in Section 2.3, and the proposed expectile regression estimation in Section 2.2 is a special case of WCER estimation, while the least squares estimation in Section 2.1 is a special case of the expectile regression estimation. In Section 2.4, we show that the asymptotic efficiency of WCER estimators calculated using weights obtained through data-driven methods is the same as those of WCER estimators calculated using known weights. We compare the least squares estimation, quantile regression estimation, expectile regression estimation and weighted composite expectile regression estimation of DTARCH models based on the maximum likelihood estimation in Section 3. The proposed methodology is also applied to analyze the daily Hang Seng Index (HSI) and the Standard & Poor’s 500 Composite Index (SPI) in Section 4. We summarize our work in Section 5. Also, for readers interested in the theoretical basis of our results, the proofs of our theoretical results are provided in Appendix A. In addition, some of our simulation results are given in Appendix B.

## 2. Estimation of the DTARCH Model

Ref. [1] proposed the DTARCH model based on the autoregressive conditional heteroskedasticity model (ARCH) model (see [35]) and the threshold model (see [36]). The DTARCH model can handle situations where both conditional mean and conditional variance specifications are piecewise linear based on previous information. Given a time series $y_{t}$, t=1,2,⋯,n, let $F_{t}$ be the σ-field generated from the realized value $\left\{y_{t},y_{t-1},\cdots \right\}$ at time *t*. Assume that $y_{t}$ is generated by

(1) $$\begin{array}{c} y_{t}=x_{t,j}^{⊤}\alpha^{\left(j\right)}+\epsilon_{t},\;\;\;\;\;\mathrm{if}\;\;r_{j-1}<y_{t-d}\leq r_{j}, \end{array}$$

where j=1,2,⋯,m; the delay parameter *d* is a positive integer; the threshold parameters $r_{j}$ satisfy $-\infty =r_{0}<r_{1}<\cdots <r_{m}=\infty$; $x_{t,j}={\left(1,y_{t-1},\cdots ,y_{t-p_{j}}\right)}^{⊤}$ is a $(p_{j}+1)\times 1$ vector of lagged variables; and $\alpha^{\left(j\right)}={\left(\alpha_{0}^{\left(j\right)},\alpha_{1}^{\left(j\right)},\cdots ,\alpha_{p_{j}}^{\left(j\right)}\right)}^{⊤}$ is a $(p_{j}+1)\times 1$ parameter vector. The stochastic error satisfies $\epsilon_{t}=\sqrt{h_{t}\left(\gamma \right)}u_{t}$ with

(2) $$\begin{array}{c} h_{t}\left(\gamma \right)=\sum_{j=1}^{m}I_{t,j}\left(\gamma_{0}^{\left(j\right)}+\gamma_{1}^{\left(j\right)}\epsilon_{t-1}^{2}+\cdots +\gamma_{q_{j}}^{\left(j\right)}\epsilon_{t-q_{j}}^{2}\right), \end{array}$$

where $I_{t,j}=I\left(r_{j-1}<y_{t-d}\leq r_{j}\right)$, and $\gamma =\mathrm{vec}\left(\gamma^{\left(1\right)},\cdots ,\gamma^{\left(m\right)}\right)$ with $\gamma^{\left(j\right)}={\left(\gamma_{0}^{\left(j\right)},\gamma_{1}^{\left(j\right)},\cdots ,\gamma_{q_{j}}^{\left(j\right)}\right)}^{⊤}$ is a $(q_{j}+1)\times 1$ parameter vector, j=1,⋯,m. Because (2) is an ARCH process, the innovations $u_{t}$ are independently and identically distributed random variables with $E(u_{t})=0$, $\mathrm{Var}(u_{t})=1$, the parameters $\gamma_{0}^{\left(j\right)}>0$, $\gamma_{i}^{\left(j\right)}\geq 0$$\left(i=1,\cdots ,q_{j}\right)$ and $\sum_{i=1}^{q_{j}}\gamma_{i}^{\left(j\right)}<1$. This is the DTARCH model proposed by [1]. A significant characteristic of DTARCH models is that their conditional mean and conditional variance functions both are piecewise linear involving double thresholds.

We have made a slight modification to the DTARCH model under consideration. As reported by [3,4], the stochastic error satisfies $\epsilon_{t}=h_{t}\left(\beta \right)u_{t}$ with

(3) $$\begin{array}{c} h_{t}\left(\beta \right)=\sum_{j=1}^{m}I_{t,j}\left(\beta_{0}^{\left(j\right)}+\beta_{1}^{\left(j\right)}|\epsilon_{t-1}|+\cdots +\beta_{q_{j}}^{\left(j\right)}\left|\epsilon_{t-q_{j}}\right|\right)\equiv \sum_{j=1}^{m}I_{t,j}z_{t,j}^{⊤}\beta^{\left(j\right)}, \end{array}$$

where $z_{t,j}={\left(1,|\epsilon_{t-1}\left|,\cdots ,\right|\epsilon_{t-q_{j}}|\right)}^{⊤}$, $\beta =\mathrm{vec}\left(\beta^{\left(1\right)},\cdots ,\beta^{\left(m\right)}\right)$ with $\beta^{\left(j\right)}={\left(\beta_{0}^{\left(j\right)},\beta_{1}^{\left(j\right)},\cdots ,\beta_{q_{j}}^{\left(j\right)}\right)}^{⊤}$ (j=1,⋯,m), and $\beta_{0}^{\left(j\right)}>0$, $\beta_{i}^{\left(j\right)}\geq 0$$\left(i=1,\cdots ,q_{j}\right)$. The innovations $u_{t}$ are independently and identically distributed random variables with an unknown distribution F(u) and a density function f(u).

Let $\alpha =\mathrm{vec}\left(\alpha^{\left(1\right)},\cdots ,\alpha^{\left(m\right)}\right)$, $x_{t}=\mathrm{vec}\left(I_{t,1}x_{t,1},\cdots ,I_{t,m}x_{t,m}\right)$, $z_{t}=\mathrm{vec}\left(I_{t,1}z_{t,1},\cdots ,I_{t,m}z_{t,m}\right)$, and denote $\sum_{j=1}^{m}\left(p_{j}+1\right)=p$ and $\sum_{j=1}^{m}\left(q_{j}+1\right)=q$. Then Equations (1) and (3) can be written as

(4) $$\begin{array}{c} y_{t}=x_{t}^{⊤}\alpha +\epsilon_{t}, \end{array}$$

and $\epsilon_{t}=h_{t}\left(\beta \right)u_{t}$ with

(5) $$\begin{array}{c} h_{t}\left(\beta \right)=z_{t}^{⊤}\beta , \end{array}$$

respectively. As in [1,3,4], we denote the model defined by (4) and (5) by

$$\mathrm{DTARCH}(p_{1},\cdots ,p_{m};q_{1},\cdots ,q_{m}),$$

where $p_{1},\cdots ,p_{m}$ represent the autoregressive model (AR) orders in the *m* regimes and $q_{1},\cdots ,q_{m}$ denote the ARCH orders in the *m* regimes. We use the DTARCH model with a conditional scale, rather than a conditional variance, because modeling the conditional scale is very important. Previous studies emphasized that such a scale provides a more natural dispersion concept than the variance and offers substantial advantages in terms of robustness. The advantage of such an approach with conditional scale instead of conditional variance can be found in [37,38,39,40,41] and so on.

### 2.1. Least Squares Estimators of the DTARCH Model

Most of the research papers on DTARCH models are based on the condition that the threshold parameters $\left\{r_{0},r_{1},\cdots ,r_{m}\right\}$ and delay parameter *d* are known. But in real data analysis, we know that this condition is hard to meet. In the literature on threshold models, there are also a few studies that are based on scenarios where the threshold or delay parameters are unknown. For example, [42] proposed the least squares (LS) estimators for a threshold AR(1) model with an unknown threshold and proved that LS estimators of the threshold parameters were strongly consistent. Ref. [43] proposed the conditional least squares (CLS) estimators for the threshold autoregressive model with unknown threshold and delay parameters and proved that CLS estimators of the threshold parameters were convergent in distribution. In this paper, we propose the parameter estimation methods for the DTARCH model based on expectile regression theory, which includes the expectile regression estimation and the weighted composite expectile regression estimation of the DTARCH model. Note that the expectile regression estimation can be seen as a special case of the weighted composite expectile regression estimation when the expectile takes on a certain value (see Section 2.3 of this paper for details), while the least squares estimation can be seen as a special value of the expectile regression estimation (see Section 2.2 of this paper for details). Under some conditions, we can show that the proposed estimators of the threshold and delay parameters are consistent.

Using the least squares estimation method, we can obtain the least squares estimation of the DTARCH model DTARCH$\left(p_{1},\cdots ,p_{m};q_{1},\cdots ,q_{m}\right)$, ${\left({\left({\hat{\alpha }}_{0}^{LS}\right)}^{⊤},{\left({\hat{\beta }}_{0}^{LS}\right)}^{⊤}\right)}^{⊤}$. Denote the threshold parameters ${\left(r_{0},r_{1},\cdots ,r_{m}\right)}^{⊤}=r$, the least squares estimator of r by ${\hat{r}}_{0}^{LS}$, and the least squares estimator of the delay parameter *d* by ${\hat{d}}_{0}^{LS}$. However, these estimators are biased. Obviously, the distribution of $|\epsilon_{t}|$ is skewed and the log-transformation is an intuitive mechanism that can make the distribution less skewed; see [44] for details. Thus, in light of [3,4], we introduce a modified form of the model DTARCH$\left(p_{1},\cdots ,p_{m};q_{1},\cdots ,q_{m}\right)$. Let

(6) $$\begin{array}{c} \log\left\{|\epsilon_{t}\left(\alpha \right)|\right\}=\log\left\{h_{t}\left(\alpha ,\beta \right)\right\}+e_{t}, \end{array}$$

where $e_{t}=\log\left\{|u_{t}|\right\}$, and

$$h_{t}\left(\alpha ,\beta \right)=\sum_{j=1}^{m}I_{t,j}\left(\beta_{0}^{\left(j\right)}+\beta_{1}^{\left(j\right)}|\epsilon_{t-1}\left(\alpha \right)|+\cdots +\beta_{q_{j}}^{\left(j\right)}\left|\epsilon_{t-q_{j}}\left(\alpha \right)\right|\right).$$

$h_{t}\left(\alpha ,\beta \right)$ is equivalent to $h_{t}\left(\beta \right)$, as we can see that $h_{t}\left(\beta \right)$ is also related to α. Therefore, we rename $h_{t}\left(\beta \right)$ as $h_{t}\left(\alpha ,\beta \right)$. Apply the least squares method again, we can obtain LS estimators of DTARCH$\left(p_{1},\cdots ,p_{m};q_{1},\cdots ,q_{m}\right)$ denoted by ${\left({\left({\hat{\alpha }}^{LS}\right)}^{⊤},{\left({\hat{\beta }}^{LS}\right)}^{⊤}\right)}^{⊤}$, ${\hat{r}}^{LS}$ and ${\hat{d}}^{LS}$, respectively. We study the properties of the least squares estimators under the following conditions.

For j=1,2,⋯,m, suppose that $x_{t,j}$ are all Markov chains. Their *l*-step transition probability is denoted by $P^{l}\left(x_{j},A_{j}\right)$, where $x_{j}\in R^{p}$ and $A_{j}$ are Borel sets. Later on, we will need the following set of regularity conditions.

(C1) $\left\{x_{t,j}\right\}$ admits a unique invariant measure $\pi_{j}\left(\cdot \right)$ such that ∃$K_{j}$, $\rho_{j}<1$, $\forall x_{j}\in R^{p}$, $\forall n_{j}\in N$, $∥P^{n_{j}}\left(x_{j},\cdot \right)-\pi_{j}\left(\cdot \right)∥\leq K_{j}\left(1+\right|x_{j}\left|\right)\rho_{j}^{n_{j}}$, where ∥·∥ and |·| denote the total variation norm and the Euclidean norm, respectively.
(C2) $E|y_{t}{|}^{2+\delta }<+\infty$ for δ>0, and $\left\{y_{t}\right\}$ is strictly stationary and ergodic.
(C3) $\left\{y_{t}\right\}$ has the first derivative, and the stationary distribution of the derivative admits a density positive everywhere.
(C4) Error $e_{t}$ has the cumulative distribution function G(·) with density g(·) being positive and having a continuous second derivative. Furthermore, $E(e_{t}^{4})<+\infty$.

Let $\alpha^{*}$, $\beta^{*}$, $d^{*}$ and $r^{*}$ be the true values of α, β, *d* and r, respectively. Then, we can obtain the following theorems and corollary.

**Theorem** **1.** *Suppose that the conditions (C2) and (C3) hold. Then, the estimators $\left(\begin{array}{c} {\hat{\alpha }}^{LS}\\ {\hat{\beta }}^{LS} \end{array}\right)$ are strongly consistent, that is,*

$$\left(\begin{array}{c} {\hat{\alpha }}^{LS}\\ {\hat{\beta }}^{LS} \end{array}\right)\overset{\;a.s.\;}{\to }\left(\begin{array}{c} \alpha^{*}\\ \beta^{*} \end{array}\right).$$

Under some additional conditions, we have the following corollary.

**Corollary** **1.** *Suppose that the conditions (C1), (C2) and (C4) hold. Then it follows from Theorem 1 that the estimator ${\hat{d}}^{LS}$ is strongly consistent, that is,*

$${\hat{d}}^{LS}\overset{\;a.s.\;}{\to }d^{*}.$$

**Theorem** **2.** Suppose that the conditions (C1), (C2) and (C4) hold. Then, the estimator ${\hat{r}}^{LS}$ converges to $r^{*}$ in distribution, that is,

$${\hat{r}}^{LS}\overset{\;D\;}{\to }r^{*}.$$

According to Corollary 1 and Theorem 2, both the threshold and delay parameters converge to their true values. Therefore, after obtaining estimated values for threshold and delay parameters, estimating the remaining parameters of the DTARCH model will yield convergence properties that are equivalent to those obtained by estimating the parameters using the known threshold and delay parameters. In order to simplify the theoretical analysis, without loss of generality, we assume that the threshold and delay parameters are known throughout the remainder of this paper.

### 2.2. Expectile Regression Estimators of the DTARCH Model

The definition of expectile regression proposed by [6] states that the τ-th expectile of a random variable *u* can be obtained minimizing the following check function,

$$Q_{\tau }\left(u\right)=\left\{\begin{array}{cc} \left(1-\tau \right)u^{2}, & u\leq 0,\\ \tau u^{2}, & u>0, \end{array}\right.$$

and the derivative $\frac{1}{2}{\dot{Q}}_{\tau }\left(u\right)=\left\{\begin{array}{cc} (1-\tau )u, & u\leq 0,\\ \tau u, & u>0, \end{array}\right.$ satisfies

$$E{\dot{Q}}_{\tau }\left(u\right)=0,$$

i.e.,

$$\left(1-\tau \right)\int_{-\infty }^{0}uf\left(u\right)du+\tau \int_{0}^{\infty }uf\left(u\right)du=0,$$

where f(·) is the density function of *u*. Therefore, the τ-th expectile of *u* is 0.

Let $\epsilon_{t}\left(\alpha \right)=y_{t}-x_{t}^{⊤}\alpha =h_{t}\left(\alpha ,\beta \right)u_{t}$ and the τ-th expectile of $u_{t}$ be μ(τ). Based on Theorem 1 in [6], the τ-th conditional expectile of $\epsilon_{t}$ given $F_{t-1}$ is

$$\mu_{\tau }\left(\epsilon_{t}|F_{t-1}\right)=h_{t}\left(\alpha ,\beta \right)\mu \left(\tau \right).$$

The τ-th expectile regression (ER) estimator of α and β can be obtained by minimizing

(7) $$\begin{array}{c} \sum_{t=s+1}^{n}Q_{\tau }\left\{\frac{\epsilon_{t}\left(\alpha \right)}{h_{t}\left(\alpha ,\beta \right)}-b_{\tau }\right\}, \end{array}$$

over $b_{\tau }$, α and β, where 0<τ<1, $s=\max\left(p_{1},\cdots ,p_{m},q_{1},\cdots ,q_{m}\right)$ and $b_{\tau }$ is the τ-th expectile of $u_{t}$. Let the resulting estimators from (7) be ${\left({\hat{b}}_{\tau }^{ER},{\left({\hat{\alpha }}_{0}^{ER}\right)}^{⊤},{\left({\hat{\beta }}_{0}^{ER}\right)}^{⊤}\right)}^{⊤}$. Not surprisingly, these estimators are also biased. To correct the bias, we should still perform expectile regression estimation on the DTARCH model (6) that has undergone a logarithmic transformation.

From model (6), the τ-th expectile of $\log\left|\epsilon_{t}\left(\alpha \right)\right|$ given $F_{t-1}$ is

$$\left(\left.\log\left|\epsilon_{t}\left(\alpha \right)\right|\right|F_{t-1}\right)=\log\left(h_{t}\left(\alpha ,\beta \right)\right)+c_{\tau },$$

where $c_{\tau }$ is the τ-th expectile of $e_{t}$. Applying the expectile regression scheme, we can obtain the expectile regression estimators of $c_{\tau }$, α and β by minimizing

(8) $$\begin{array}{c} \sum_{t=s+1}^{n}Q_{\tau }\left(\log(|\epsilon_{t}\left(\alpha \right)\left|\right)-\log\left\{h_{t}\left(\alpha ,\beta \right)\right\}-c_{\tau }\right), \end{array}$$

over $c_{\tau }$, α and β. Obviously, when τ=0.5, the expectile regression estimators are the least square estimators in Section 2.1.

Let the resulting estimators of ${\left(c_{\tau },\alpha^{⊤},\beta^{⊤}\right)}^{⊤}$ be ${\left({\hat{c}}_{\tau }^{ER},{\left({\hat{\alpha }}^{ER}\right)}^{⊤},{\left({\hat{\beta }}^{ER}\right)}^{⊤}\right)}^{⊤}$. To derive the asymptotic property of the proposed estimator, we introduce some notations and conditions. Let $c_{\tau }^{*}$ be the τ-th expectile of $e_{t}$, $\epsilon_{t}=\epsilon_{t}\left(\alpha^{*}\right)$, $h_{t}=h_{t}\left(\alpha^{*},\beta^{*}\right)$, $r_{t}=\frac{x_{t}}{\epsilon_{t}}+\frac{f_{t}}{h_{t}}$ with

$$f_{t}=-\sum_{j=1}^{m}I_{t,j}\left(0,\mathrm{sgn}\left(\epsilon_{t-1}\right)x_{t-1},\cdots ,\mathrm{sgn}\left(\epsilon_{t-q_{j}}\right)x_{t-q_{j}}\right)\beta^{\left(j\right)},$$

$\mu ={\left(\mu_{r}^{⊤},\mu_{z}^{⊤}\right)}^{⊤}$ with $\mu_{r}=E\left(r_{t}\right)$ and $\mu_{z}=E\left(z_{t}/h_{t}\right)$, $\Omega =\left(\begin{array}{cc} \Omega_{r} & \Omega_{rz}\\ \Omega_{rz}^{⊤} & \Omega_{z} \end{array}\right)$ with $\Omega_{r}=E\left(r_{t}r_{t}^{⊤}\right)$, $\Omega_{rz}=E\left(\frac{z_{t}}{h_{t}}r_{t}^{⊤}\right)$ and $\Omega_{z}=E\left(z_{t}z_{t}^{⊤}h_{t}^{-2}\right)$ and $\Pi =\left(\begin{array}{cc} \Pi_{r} & \Pi_{rz}\\ \Pi_{rz}^{⊤} & \Pi_{z} \end{array}\right)$ with $\Pi_{r}=\mathrm{Var}\left(r_{t}\right)$, $\Pi_{rz}=\mathrm{Cov}\left(r_{t},\frac{z_{t}}{h_{t}}\right)$ and $\Pi_{z}=\mathrm{Var}\left(\frac{z_{t}}{h_{t}}\right)$. We assume that

(C5) Covariance matrix Π is positive definite.

Then we have the following asymptotic results for ${\left({\hat{c}}_{\tau }^{ER},{\left({\hat{\alpha }}^{ER}\right)}^{⊤},{\left({\hat{\beta }}^{ER}\right)}^{⊤}\right)}^{⊤}$.

**Theorem** **3.** Suppose that the conditions (C2), (C4) and (C5) hold. Then, ${\left({\hat{c}}_{\tau }^{ER},{\left({\hat{\alpha }}^{ER}\right)}^{⊤},{\left({\hat{\beta }}^{ER}\right)}^{⊤}\right)}^{⊤}$ converge to ${\left(c_{\tau }^{*},{\left(\alpha^{*}\right)}^{⊤},{\left(\beta^{*}\right)}^{⊤}\right)}^{⊤}$ in distribution, that is

$$\sqrt{n}{\left({\hat{c}}_{\tau }^{ER}-c_{\tau }^{*},{\left({\hat{\alpha }}^{ER}-\alpha^{*}\right)}^{⊤},{\left({\hat{\beta }}^{ER}-\beta^{*}\right)}^{⊤}\right)}^{⊤}\overset{\;D\;}{\to }N\left(0,\Psi \right),$$

*where* Ψ *is a block matrix with blocks*

$$\begin{array}{c} \Psi_{11}=\zeta \left(1+\mu^{⊤}\Pi^{-1}\mu \right),\;\;\;\;\;\Psi_{12}=-\zeta \left(\mu^{⊤}\Pi^{-1}\right),\\ \Psi_{21}=-\zeta \left(\Pi^{-1}\mu \right),\;\;\;\;\;\Psi_{22}=\zeta \Pi^{-1}, \end{array}$$

with $\zeta =\frac{1}{4}{\left[\left(1-2\tau \right)G\left(c_{\tau }^{*}\right)+\tau \right]}^{-2}E\left[{\dot{Q}}_{\tau }^{2}\left(e_{t}-c_{\tau }^{*}\right)\right]$.

By Theorem 3, it follows that

$$\sqrt{n}\left({\hat{c}}_{\tau }^{ER}-c_{\tau }^{*}\right)\overset{\;D\;}{\to }N\left(0,\Psi_{11}\right),$$

$$\sqrt{n}\left(\begin{array}{c} {\hat{\alpha }}^{ER}-\alpha^{*}\\ {\hat{\beta }}^{ER}-\beta^{*} \end{array}\right)\overset{\;D\;}{\to }N\left(0,\Psi_{22}\right).$$

### 2.3. Weight Composite Expectile Regression Estimators of the DTARCH Model

Refs. [45,46,47] considered the composite quantile regression (CQR) estimation, which is obtained by incorporating the information of multiple quantiles into the objective function. This estimation method incorporates more comprehensive model information. Subsequently, ref. [29] introduced an estimation called composite expectile regression (CER) and established large sample properties of the resulting CER estimator. However, both CQR and CER estimations assign equal weights to different quantiles and expectiles, respectively. Intuitively, using different weights for different quantile regression (QR) and expectile regression (ER) models might lead to improved efficiency. Hence, ref. [48,49] proposed the weighted composite quantile regression (WCQR) estimation method. The standard deviation (SD) of the WCQR estimator is smaller than the SD of the CQR estimator and QR estimator as discussed in [4]. Furthermore, ref. [26] proposed a weighted composite expectile regression (WCER) estimation for AR models and established its large sample’properties.

From model (6), the $\tau_{k}$-th expectile of $\log\left|\epsilon_{t}\left(\alpha \right)\right|$ given $F_{t-1}$ is

$$\left(\left.\log\left|\epsilon_{t}\left(\alpha \right)\right|\right|F_{t-1}\right)=\log\left(h_{t}\left(\alpha ,\beta \right)\right)+c_{\tau_{k}},$$

where $c_{\tau_{k}}$ is the $\tau_{k}$-th expectile of $e_{t}$. Applying the WCER scheme, we can jointly estimate the AR and ARCH parameters by minimizing

(9) $$\begin{array}{c} \sum_{k=1}^{K}\omega_{k}\sum_{t=s+1}^{n}Q_{\tau_{k}}\left(\log(|\epsilon_{t}\left(\alpha \right)\left|\right)-\log\left\{h_{t}\left(\alpha ,\beta \right)\right\}-c_{\tau_{k}}\right), \end{array}$$

over $c_{\tau_{k}}$, α and β, where $\omega ={\left(\omega_{1},\cdots ,\omega_{K}\right)}^{⊤}$ is a vector of weights such that ∥ω∥=1, with ∥·∥ denoting the Euclidean norm. Without loss of generality, we assume that $0<\tau_{1}<\cdots <\tau_{K}<1$. If $\omega_{i}=1/\sqrt{K}$, the estimation obtained from Equation (9) is CER estimation. Obviously, the weight $\omega_{k}$ is the contribution rate of the $\tau_{k}$-th expectile. Since $Q_{\tau_{k}}\left(\log(\right|\epsilon_{t}\left(\alpha \right)|)-\log h_{t}\left(\alpha ,\beta \right)-c_{\tau_{k}})$ may not have a positive correlation, it is possible for the weight component ω to be negative. Therefore, the WCER estimation is not a simple extension of the CER estimation. Due to the limited space, we will not discuss the CER estimation in detail.

Let the resulting estimator of ${\left(c_{\tau_{1}},\cdots ,c_{\tau_{K}},\alpha^{⊤},\beta^{⊤}\right)}^{⊤}$ be ${\left({\hat{c}}_{\tau_{1}},\cdots ,{\hat{c}}_{\tau_{K}},{\hat{\alpha }}^{⊤},{\hat{\beta }}^{⊤}\right)}^{⊤}$ and $c_{\tau_{k}}^{*}$ be the true value of the $\tau_{k}$-th expectile of $e_{t}$. Under certain conditions, we have the following asymptotic results for ${\left({\hat{c}}_{\tau_{1}},\cdots ,{\hat{c}}_{\tau_{K}},{\hat{\alpha }}^{⊤},{\hat{\beta }}^{⊤}\right)}^{⊤}$.

**Theorem** **4.** Suppose that the conditions (C2), (C4) and (C5) hold. Then ${\left({\hat{c}}_{\tau_{1}},\cdots ,{\hat{c}}_{\tau_{K}},{\hat{\alpha }}^{⊤},{\hat{\beta }}^{⊤}\right)}^{⊤}$ converge to ${\left(c_{\tau_{1}}^{*},\cdots ,c_{\tau_{K}}^{*},{\left(\alpha^{*}\right)}^{⊤},{\left(\beta^{*}\right)}^{⊤}\right)}^{⊤}$ in distribution, that is

$$\sqrt{n}{\left({\hat{c}}_{\tau_{1}}-c_{\tau_{1}}^{*},\cdots ,{\hat{c}}_{\tau_{K}}-c_{\tau_{K}}^{*},{\left(\hat{\alpha }-\alpha^{*}\right)}^{⊤},{\left(\hat{\beta }-\beta^{*}\right)}^{⊤}\right)}^{⊤}\overset{\;D\;}{\to }N\left(0,\Sigma \right),$$

*where* Σ *is a block matrix with blocks*

$$\begin{array}{c} \Sigma_{11}=\xi +\sigma^{2}\left(\omega \right)\left(\mu^{⊤}\Pi^{-1}\mu \right)11^{⊤},\;\;\;\;\;\Sigma_{12}=-\sigma^{2}\left(\omega \right)1⊗\left(\mu^{⊤}\Pi^{-1}\right),\\ \Sigma_{21}=-\sigma^{2}\left(\omega \right)1^{⊤}⊗\left(\Pi^{-1}\mu \right),\;\;\;\;\;\Sigma_{22}=\sigma^{2}\left(\omega \right)\Pi^{-1}, \end{array}$$

*with* $1={\left(1,\cdots ,1\right)}^{⊤}$*, ξ is a K×K matrix with the (j,k)th element being*

$$\xi_{jk}=\frac{E\left[{\dot{Q}}_{\tau_{j}}\left(e_{t}-c_{\tau_{j}}^{*}\right){\dot{Q}}_{\tau_{k}}\left(e_{t}-c_{\tau_{k}}^{*}\right)\right]}{4\left[\left(1-2\tau_{j}\right)G\left(c_{\tau_{j}}^{*}\right)+\tau_{j}\right]\left[\left(1-2\tau_{k}\right)G\left(c_{\tau_{k}}^{*}\right)+\tau_{k}\right]},$$

$\sigma^{2}\left(\omega \right)=\frac{\omega^{⊤}\Lambda \omega }{{\left[\sum_{k=1}^{K}\omega_{k}\left(\left(1-2\tau_{k}\right)G\left(c_{\tau_{k}}^{*}\right)+\tau_{k}\right)\right]}^{2}}$, Λ* is also a K×K matrix with the (j,k)th element being*

$$\begin{array}{cc} \Lambda \left(j,k\right) & =\frac{1}{4}E\left\{{\dot{Q}}_{\tau_{j}}\left(e_{t}-c_{\tau_{j}}^{*}\right){\dot{Q}}_{\tau_{k}}\left(e_{t}-c_{\tau_{k}}^{*}\right)\right\}\\ & =\int \left|\tau_{j}\tau_{k}-\tau_{j}I\left(e_{t}\leq c_{\tau_{k}}^{*}\right)-\tau_{k}I\left(e_{t}\leq c_{\tau_{j}}^{*}\right)+I\left(e_{t}\leq c_{\tau_{j}}^{*}\wedge c_{\tau_{k}}^{*}\right)\right|\\ & \;\;\times \left[e_{t}^{2}-\left(c_{\tau_{j}}^{*}+c_{\tau_{k}}^{*}\right)e_{t}+c_{\tau_{j}}^{*}c_{\tau_{k}}^{*}\right]dG\left(e_{t}\right). \end{array}$$

### 2.4. Selection of Optimal Weight

By Theorem 4, we have

$$\sqrt{n}\left(\begin{array}{c} \hat{\alpha }-\alpha^{*}\\ \hat{\beta }-\beta^{*} \end{array}\right)\overset{\;D\;}{\to }N\left(0,\Sigma_{22}\right),$$

where $\Sigma_{22}=\sigma^{2}\left(\omega \right)\Pi^{-1}$. Because Π does not contain weight ω, to obtain the optimal weight, we only need to minimize $\sigma^{2}\left(\omega \right)$ under the condition of ∥ω∥=1, which yields

$$\omega_{\mathrm{opt}}={\left(g^{⊤}\Lambda^{-2}g\right)}^{-1/2}\Lambda^{-1}g,$$

where

$$g={\left(\left(1-2\tau_{1}\right)G\left(c_{\tau_{1}}^{*}\right)+\tau_{1},\cdots ,\left(1-2\tau_{K}\right)G\left(c_{\tau_{K}}^{*}\right)+\tau_{K}\right)}^{⊤}.$$

The cumulative distribution function G(·) with density g(·) of error $\left\{e_{t}\right\}$ can be obtained by the kernel smooth estimation. Then, the nonparametric estimator of $\omega_{\mathrm{opt}}$ is given by

$$\hat{\omega }={\left({\hat{\omega }}_{1},\cdots ,{\hat{\omega }}_{K}\right)}^{⊤}={\left({\hat{g}}^{⊤}{\hat{\Lambda }}^{-2}\hat{g}\right)}^{-1/2}{\hat{\Lambda }}^{-1}\hat{g},$$

so that estimator of $\left(\begin{array}{c} \alpha \\ \beta \end{array}\right)$ denoted by $\left(\begin{array}{c} {\hat{\alpha }}_{0}\\ {\hat{\beta }}_{0} \end{array}\right)$ can be obtained by the following formula,

(10) $$\begin{array}{c} \min_{c_{\tau_{k}},\alpha ,\beta }\sum_{k=1}^{K}{\hat{\omega }}_{k}\sum_{t=s+1}^{n}Q_{\tau_{k}}\left(\log(|\epsilon_{t}\left(\alpha \right)\left|\right)-\log\left\{h_{t}\left(\alpha ,\beta \right)\right\}-c_{\tau_{k}}\right). \end{array}$$

Then, under certain conditions, we have the following asymptotic results for $\left(\begin{array}{c} {\hat{\alpha }}_{0}\\ {\hat{\beta }}_{0} \end{array}\right)$.

**Corollary** **2.** Suppose that the conditions (C2), (C4) and (C5) hold. Then $\left(\begin{array}{c} {\hat{\alpha }}_{0}\\ {\hat{\beta }}_{0} \end{array}\right)$ converge to $\left(\begin{array}{c} \alpha^{*}\\ \beta^{*} \end{array}\right)$ in distribution, that is

$$\sqrt{n}\left(\begin{array}{c} {\hat{\alpha }}_{0}-\alpha^{*}\\ {\hat{\beta }}_{0}-\beta^{*} \end{array}\right)\overset{\;D\;}{\to }N\left(0,{\left(g^{⊤}\Lambda^{-1}g\right)}^{-1}\Pi^{-1}\right).$$

Note that $\sigma^{2}\left(\omega_{\mathrm{opt}}\right)={\left(g^{⊤}\Lambda^{-1}g\right)}^{-1}$. When $\omega_{\mathrm{opt}}$ is known, the asymptotic covariance of $\left(\begin{array}{c} {\hat{\alpha }}_{0}\\ {\hat{\beta }}_{0} \end{array}\right)$ is the same as the covariance variance of $\left(\begin{array}{c} \hat{\alpha }\\ \hat{\beta } \end{array}\right)$. In other words, the asymptotic efficiency of WCER estimators calculated using weights obtained through data-driven optimal weighting is the same as those of WCER estimators calculated using known weights.

## 3. Comparison of Estimation Methods

In this section, we compare the least squares estimation, quantile regression estimation, expectile regression estimation and weighted composite expectile regression estimation of DTARCH models by using the maximum likelihood estimation (MLE) of DTARCH models as the benchmark. We consider the following DTARCH(2,2;2,2) model:

$$y_{t}=\left\{\begin{array}{cc} \alpha_{1}^{\left(1\right)}y_{t-1}+\alpha_{2}^{\left(1\right)}y_{t-2}+\epsilon_{t}, & \mathrm{if}\;y_{t-1}\leq 0,\\ \alpha_{1}^{\left(2\right)}y_{t-1}+\alpha_{2}^{\left(2\right)}y_{t-2}+\epsilon_{t}, & \mathrm{if}\;y_{t-1}>0, \end{array}\right.$$

where $\left(\alpha_{1}^{\left(1\right)},\alpha_{2}^{\left(1\right)}\right)=\left(0.25,0.30\right)$, $\left(\alpha_{1}^{\left(2\right)},\alpha_{2}^{\left(2\right)}\right)=\left(0.45,0.20\right)$ and $\epsilon_{t}=h_{t}u_{t}$, with

$$h_{t}=\left\{\begin{array}{cc} \beta_{0}^{\left(1\right)}+\beta_{1}^{\left(1\right)}\left|\epsilon_{t-1}\right|+\beta_{2}^{\left(1\right)}\left|\epsilon_{t-2}\right|, & \mathrm{if}\;y_{t-1}\leq 0,\\ \beta_{0}^{\left(2\right)}+\beta_{1}^{\left(2\right)}\left|\epsilon_{t-1}\right|+\beta_{2}^{\left(2\right)}\left|\epsilon_{t-2}\right|, & \mathrm{if}\;y_{t-1}>0, \end{array}\right.$$

where $\left(\beta_{0}^{\left(1\right)},\beta_{1}^{\left(1\right)},\beta_{2}^{\left(1\right)}\right)=\left(0.03,0.30,0.50\right)$, $\left(\beta_{0}^{\left(2\right)},\beta_{1}^{\left(2\right)},\beta_{2}^{\left(2\right)}\right)=\left(0.06,0.45,0.35\right)$.

We consider three types of innovation variables, which are distributed as N(0,1), t(6) and $\chi^{2}\left(4\right)$. They are centralized and normalized so that the medians of the absolute innovations are 1, i.e., $u_{t}$ is normalized to satisfy Median$(|u_{t}|)=1$. The sample size *n* is chosen are 100, 300, 800, 1500 and 2500. All the simulation results are based on 500 Monte Carlo replications. Seven equally spaced expectiles in (0,1) are chosen for each simulation setting when we apply the WCER estimation process. For QR and ER estimation, we take τ=0.25 and 0.75, respectively. In each simulation, the root mean squared error (RMSE) for different estimators are calculated, and they are reported in Table 1, Table 2 and Table 3. In addition, the parameter estimators obtained from different estimation methods of the DTARCH model are listed in Table A1, Table A2 and Table A3 in Appendix B.

As expected, the oracle MLE performs the best, while the WCER estimators outperform both the QR estimators and the ER estimators. As can be seen from Table 1 and Table A1, the WCER estimators slightly underperform LS estimators only when the residual error follows a normal distribution. From Table 2, Table 3, Table A2 and Table A3, we can see that the WCER estimators greatly outperform the LS estimators in terms of RMSE when the error follows a heavy-tailed or asymmetric distribution. In studies that apply time series models to study financial data, it is more realistic to assume that the error follows a non-normal and heavy-tailed distribution. For example, [50] considered the heavy-tailed nature and extreme volatility of asset returns, and demonstrated these statistical characteristics using financial data. Ref. [51] introduced a new heavy-tailed distribution to characterize errors in the ARCH/GARCH model and applied it to financial data. Furthermore, [52] assumed that there are two different types of heavy-tail distributions for GARCH model errors: the student’s t-distribution and the normal reciprocal inverse Gaussian distribution. They compared the application of these distributions to South Korea’s daily stock market returns.

Therefore, it is possible to obtain a WCER estimator with favorable statistical properties similar to those of the MLE, even when the distribution of the error is unknown. Moreover, the RMSEs of all estimation methods decrease with the increase of sample size *n*, indicating that all estimators are consistent.

We also make an empirical analysis of the DTARCH model with delay parameter d≠1. Similarly, based on the maximum likelihood estimation, we compare the QR estimation, ER estimation and WCER estimation of this DTARCH model. We present the simulation results in the supplementary materials.

## 4. Real Data Analysis

In this section, we use the proposed method to analyze the Hang Seng Index (HSI) and the Standard & Poor’s 500 Index (SPI) daily from 7 February 2013, to 6 February 2023. The formula for calculating returns series $y_{t}$ uses the daily returns of the exponential market, which are represented by the first-order difference of the logarithm of the closing prices of the index on adjacent days,

$$y_{t}=\log\left(x_{t}\right)-\log\left(x_{t-1}\right),$$

where $x_{t}$ represents the closing price of the HSI or SPI on day *t*. The sample size for SPI is n=2516, and the sample size for HSI is n=2453. We are interested in the asymmetry of the conditional mean and conditional variance of the stock market.

First, we need to identify the values of *m*, the delay parameter *d* and the threshold parameters $r_{i}$. Applying the same method as in [3], we obtain the values of *m*, *d* and $r_{i}$ as d=1, m=2 and $\left(r_{0},r_{1},r_{2}\right)=\left(-\infty ,0,+\infty \right)$. This aligns with stock market observations and supports our goal of examining the asymmetry in conditional mean and variance. Similar to [3,4], we employ the generalized Akaike information criterion (GAIC) and the generalized Bayesian information criterion (GBIC) methods to determine the orders of DTARCH models before fitting the models to HSI and SPI. We find out that both GAIC and GBIC reach their minimum values for HSI and SPI with p=2 and q=4. The minimum GAIC and GBIC values for HSI are 0.3144 and 0.3876, respectively. The minimum GAIC and GBIC values for SPI are 0.2975 and 0.3463, respectively. This designates a DTARCH(2,2;4,4) model for the return series. Thus, the following DTARCH model is taken into account for the return series $y_{t}$,

(11) $$\begin{array}{c} y_{t}=\left\{\begin{array}{cc} \alpha_{1}^{\left(1\right)}y_{t-1}+\alpha_{2}^{\left(1\right)}y_{t-2}+\epsilon_{t}, & \mathrm{if}\;y_{t-1}\leq 0,\\ \alpha_{1}^{\left(2\right)}y_{t-1}+\alpha_{2}^{\left(2\right)}y_{t-2}+\epsilon_{t}, & \mathrm{if}\;y_{t-1}>0, \end{array}\right. \end{array}$$

and $\epsilon_{t}=h_{t}u_{t}$, with

(12) $$\begin{array}{c} h_{t}=\left\{\begin{array}{cc} \beta_{0}^{\left(1\right)}+\beta_{1}^{\left(1\right)}\left|\epsilon_{t-1}\right|+\beta_{2}^{\left(1\right)}\left|\epsilon_{t-2}\right|+\beta_{3}^{\left(1\right)}\left|\epsilon_{t-3}\right|+\beta_{4}^{\left(1\right)}\left|\epsilon_{t-4}\right|, & \mathrm{if}\;y_{t-1}\leq 0,\\ \beta_{0}^{\left(2\right)}+\beta_{1}^{\left(2\right)}\left|\epsilon_{t-1}\right|+\beta_{2}^{\left(2\right)}\left|\epsilon_{t-2}\right|+\beta_{3}^{\left(2\right)}\left|\epsilon_{t-3}\right|+\beta_{4}^{\left(2\right)}\left|\epsilon_{t-4}\right|, & \mathrm{if}\;y_{t-1}>0. \end{array}\right. \end{array}$$

For comparison, we calculate the proposed WCER estimate, ER estimate, QR estimate and LS estimate, respectively. Seven equally spaced expectiles in (0,1) are chosen when we apply the WCER estimation process. For QR and ER estimation, we take τ=0.25 and 0.75 respectively.

These estimates and their standard errors are listed in Table 4 and Table 5, which show some interesting results. First, the estimated $\left({\hat{\alpha }}_{1}^{\left(1\right)},{\hat{\alpha }}_{2}^{\left(1\right)},{\hat{\alpha }}_{1}^{\left(2\right)},{\hat{\alpha }}_{2}^{\left(2\right)}\right)$ are all negative, which suggest that if current and past returns are negative, the forecasted mean return will be positive; if they are positive, the forecasted mean return will be negative. Second, all the estimated coefficients ${\hat{\beta }}_{0}^{\left(1\right)}$, $\left({\hat{\beta }}_{1}^{\left(1\right)},{\hat{\beta }}_{2}^{\left(1\right)},{\hat{\beta }}_{3}^{\left(1\right)},{\hat{\beta }}_{4}^{\left(1\right)}\right)$, ${\hat{\beta }}_{0}^{\left(2\right)}$ and $\left({\hat{\beta }}_{1}^{\left(2\right)},{\hat{\beta }}_{2}^{\left(2\right)},{\hat{\beta }}_{3}^{\left(2\right)},{\hat{\beta }}_{4}^{\left(2\right)}\right)$ are positive, which aligns with the expectations as the DTARCH models assume non-negative volatility coefficients. Third, the values of $\left({\hat{\beta }}_{1}^{\left(1\right)},{\hat{\beta }}_{2}^{\left(1\right)},{\hat{\beta }}_{3}^{\left(1\right)},{\hat{\beta }}_{4}^{\left(1\right)}\right)$ are significantly different from those of $\left({\hat{\beta }}_{1}^{\left(2\right)},{\hat{\beta }}_{2}^{\left(2\right)},{\hat{\beta }}_{3}^{\left(2\right)},{\hat{\beta }}_{4}^{\left(2\right)}\right)$. These show that the volatility of HSI and SPI exhibit obvious asymmetry. Fourth, the absolute values of the parameter estimates of HSI are almost greater than those of SPI. This shows that the market volatility of HSI is higher than that of SPI. Indeed, over the past 20 years, the volatility of the S&P 500 Index has been lower than that of the Hang Seng Index, leading some to believe that the former’s returns would be lower than the latter. However, since the S&P 500 Index has experienced smaller market drops in the past compared to the Hang Seng Index, its absolute returns have been higher over the past 20 years and both its short-term and long-term risk-adjusted returns are higher as well.

To evaluate the predictive performance of the models we built, we split the dataset into two parts: a larger part for model building and a smaller part for model validation. For example, we split the SPI dataset with sample size n=2516 into two subsets: sample 1 (the sample from 7 February 2013 to 21 December 2022) of size $n_{1}=2486$ and sample 2 (the sample from 22 December 2022 to 6 February 2023) of $n_{2}=30$. We build a DARCH model using the sample 1, and then apply the model to predict the dataset from 22 December 2022 to 6 February 2023. We compare the predicted values with the sample 2 to evaluate the performance of different estimation methods. The chosen evaluation metric is Median Absolute Percentage Error (MAPE), calculated as the median absolute difference between predicted values ${\hat{y}}_{i}$ and observed values $y_{i}$ ($i=1,2,\ldots ,n_{2}$). This approach is similar to that used in [29]. We perform the same procedure on the SPI and HSI datasets with sample 2 of different sizes, specifically $n_{2}=10$, 20 and 30. The results obtained are shown in Table 6 and Table 7, respectively.

Based on the MAPE from Table 6 and Table 7, it can be see that the WCER estimation consistently produces lower MAPE values compared to the other methods. Therefore, we conclude that the WCER estimation method outperforms other methods.

## 5. Concluding Remarks

In this paper, we develop an estimation method for DTARCH models based on the expectile theory. We propose the WCER estimators for DTARCH models and derive the large sample properties of the proposed estimators. Unlike the existing papers on the study of DTARCH models, we do not need to know the threshold and delay parameters. We conduct a simulation study to test the proposed theory and find that our WCER estimator outperforms the LS estimator in terms of RMSE, particularly when the errors follow a heavy-tailed or asymmetric distribution. The simulation results are consistent with our theoretical results. Even if the common distribution of errors is unknown, we can still obtain a WCER estimator with good statistical properties like the MLE. Furthermore, we apply the proposed WCER estimation method to estimate the parameters of DTARCH models using daily returns data for HSI and SPI.

It is noted that the proposed WCER estimation method is more effective for DTARCH models when the errors follow a non-normal heavy-tail distribution. This finding is consistent with real data examples, which adds to the practical significance of our study. Therefore, our future work will focus on further practical data analysis using the proposed methods. In addition, considering the high dimensionality of many real datasets, one of our next key steps is to come up with estimates based on expectile regression theory in such a scenario. This is one of the important research tasks that we will undertake. 

## Figures and Tables

**Table 1:** RMSE comparison of various estimation methods, $\epsilon_{t}∼N\left(0,1\right)$.

	n=100									
Estimate	$\alpha_{1}^{\left(1\right)}$	$\alpha_{2}^{\left(1\right)}$	$\alpha_{1}^{\left(2\right)}$	$\alpha_{2}^{\left(2\right)}$	$\beta_{0}^{\left(1\right)}$	$\beta_{1}^{\left(1\right)}$	$\beta_{2}^{\left(1\right)}$	$\beta_{0}^{\left(2\right)}$	$\beta_{1}^{\left(2\right)}$	$\beta_{2}^{\left(2\right)}$
MLE	13.14	11.43	10.14	11.07	1.63	17.38	14.11	1.82	15.03	10.08
LS	14.83	12.72	11.63	11.97	1.93	19.97	15.37	2.31	16.61	11.27
QR${}_{0.25}$	21.16	23.47	20.09	23.43	3.09	28.97	27.62	3.99	25.38	24.13
QR${}_{0.75}$	22.37	25.64	21.47	23.31	3.31	29.93	25.89	3.68	27.15	24.47
ER${}_{0.25}$	22.51	24.18	21.21	22.19	3.14	29.91	26.71	4.01	26.93	24.51
ER${}_{0.75}$	22.79	24.65	21.35	24.09	3.19	30.07	26.96	3.99	27.24	25.48
WCER	18.75	19.78	16.71	18.45	1.98	20.09	15.61	2.41	17.52	11.98
	n=300									
**Estimate**	$\alpha_{1}^{\left(1\right)}$	$\alpha_{2}^{\left(1\right)}$	$\alpha_{1}^{\left(2\right)}$	$\alpha_{2}^{\left(2\right)}$	$\beta_{0}^{\left(1\right)}$	$\beta_{1}^{\left(1\right)}$	$\beta_{2}^{\left(1\right)}$	$\beta_{0}^{\left(2\right)}$	$\beta_{1}^{\left(2\right)}$	$\beta_{2}^{\left(2\right)}$
MLE	9.21	9.47	8.96	9.11	0.92	10.29	9.41	1.01	9.34	6.49
LS	10.11	10.38	9.11	9.32	0.99	11.10	9.54	1.14	9.49	6.75
QR${}_{0.25}$	17.98	21.42	15.98	18.76	2.19	23.89	18.91	2.59	21.99	18.49
QR${}_{0.75}$	18.99	21.39	16.09	18.94	2.29	23.94	18.99	2.63	22.03	18.53
ER${}_{0.25}$	18.91	21.01	16.67	18.92	2.22	23.71	18.85	2.67	22.31	18.56
ER${}_{0.75}$	19.12	21.05	16.71	19.01	2.32	23.79	19.01	2.68	22.45	18.67
WCER	13.42	13.32	9.58	9.97	1.01	11.43	9.88	1.12	10.12	7.18
	n=800									
**Estimate**	$\alpha_{1}^{\left(1\right)}$	$\alpha_{2}^{\left(1\right)}$	$\alpha_{1}^{\left(2\right)}$	$\alpha_{2}^{\left(2\right)}$	$\beta_{0}^{\left(1\right)}$	$\beta_{1}^{\left(1\right)}$	$\beta_{2}^{\left(1\right)}$	$\beta_{0}^{\left(2\right)}$	$\beta_{1}^{\left(2\right)}$	$\beta_{2}^{\left(2\right)}$
MLE	6.81	7.01	5.72	6.52	0.49	8.19	7.45	0.71	4.21	3.68
LS	7.45	7.21	6.14	7.11	0.54	8.92	7.94	0.79	4.35	3.81
QR${}_{0.25}$	14.96	16.57	12.68	14.38	1.79	17.09	14.48	1.89	16.71	13.09
QR${}_{0.75}$	14.99	16.66	12.74	14.46	1.88	17.04	14.49	1.94	16.79	13.11
ER${}_{0.25}$	15.02	16.65	12.65	14.31	1.86	16.92	14.46	1.92	16.62	13.16
ER${}_{0.75}$	15.36	16.78	12.87	14.41	1.96	16.98	14.52	2.02	16.87	13.18
WCER	7.65	8.05	6.86	7.56	0.60	9.21	8.19	0.86	4.56	3.99
	n=1500									
**Estimate**	$\alpha_{1}^{\left(1\right)}$	$\alpha_{2}^{\left(1\right)}$	$\alpha_{1}^{\left(2\right)}$	$\alpha_{2}^{\left(2\right)}$	$\beta_{0}^{\left(1\right)}$	$\beta_{1}^{\left(1\right)}$	$\beta_{2}^{\left(1\right)}$	$\beta_{0}^{\left(2\right)}$	$\beta_{1}^{\left(2\right)}$	$\beta_{2}^{\left(2\right)}$
MLE	4.78	5.46	3.27	3.49	0.28	6.79	3.94	0.42	2.93	2.07
LS	4.84	5.79	3.41	3.62	0.36	6.90	4.04	0.47	3.11	2.18
QR${}_{0.25}$	9.92	10.14	8.11	9.52	1.21	10.09	10.21	1.01	11.34	8.92
QR${}_{0.75}$	9.99	10.20	8.23	9.51	1.28	10.07	10.28	0.99	11.26	8.89
ER${}_{0.25}$	9.95	10.13	8.25	9.43	1.17	11.13	10.12	1.04	11.26	8.86
ER${}_{0.75}$	10.10	10.24	8.37	9.44	1.25	11.09	10.25	1.02	11.66	8.92
WCER	5.03	5.98	3.68	3.84	0.41	7.12	4.23	0.56	3.24	2.37
	n=2500									
**Estimate**	$\alpha_{1}^{\left(1\right)}$	$\alpha_{2}^{\left(1\right)}$	$\alpha_{1}^{\left(2\right)}$	$\alpha_{2}^{\left(2\right)}$	$\beta_{0}^{\left(1\right)}$	$\beta_{1}^{\left(1\right)}$	$\beta_{2}^{\left(1\right)}$	$\beta_{0}^{\left(2\right)}$	$\beta_{1}^{\left(2\right)}$	$\beta_{2}^{\left(2\right)}$
MLE	2.21	3.13	1.56	1.70	0.11	3.99	1.98	0.20	1.15	0.98
LS	2.30	3.47	1.72	1.89	0.18	4.14	2.15	0.28	1.30	1.13
QR${}_{0.25}$	4.65	5.84	4.01	4.44	0.51	7.10	5.49	0.59	5.79	4.24
QR${}_{0.75}$	4.68	5.89	4.04	4.50	0.54	7.08	5.51	0.57	5.76	4.29
ER${}_{0.25}$	4.64	5.86	4.05	4.46	0.50	7.12	5.42	0.60	5.75	4.22
ER${}_{0.75}$	4.70	5.92	4.08	4.42	0.55	7.15	5.44	0.58	5.77	4.27
WCER	2.42	3.56	1.84	2.01	0.22	4.23	2.26	0.36	1.39	1.21

Notes: All RMSEs are multiplied by $10^{2}$.

**Table 2:** RMSE comparison of various estimation methods, $\epsilon_{t}∼t\left(6\right)$.

	n=100									
Estimate	$\alpha_{1}^{\left(1\right)}$	$\alpha_{2}^{\left(1\right)}$	$\alpha_{1}^{\left(2\right)}$	$\alpha_{2}^{\left(2\right)}$	$\beta_{0}^{\left(1\right)}$	$\beta_{1}^{\left(1\right)}$	$\beta_{2}^{\left(1\right)}$	$\beta_{0}^{\left(2\right)}$	$\beta_{1}^{\left(2\right)}$	$\beta_{2}^{\left(2\right)}$
MLE	13.72	11.69	10.98	11.06	1.76	17.89	14.61	1.94	14.43	10.61
LS	20.54	23.98	19.91	21.98	2.75	27.84	23.32	3.49	23.25	19.94
QR${}_{0.25}$	20.08	23.81	20.01	21.99	2.81	28.11	24.06	3.52	23.99	19.98
QR${}_{0.75}$	20.01	23.86	20.14	22.12	2.90	28.20	24.01	3.60	24.12	21.11
ER${}_{0.25}$	19.56	23.89	20.13	22.20	2.78	28.16	24.16	3.47	24.00	20.22
ER${}_{0.75}$	20.75	23.45	20.16	22.67	2.83	28.56	24.70	3.55	24.05	20.53
WCER	19.12	21.62	16.59	19.65	1.89	25.71	17.84	2.56	19.08	12.19
	n=300									
**Estimate**	$\alpha_{1}^{\left(1\right)}$	$\alpha_{2}^{\left(1\right)}$	$\alpha_{1}^{\left(2\right)}$	$\alpha_{2}^{\left(2\right)}$	$\beta_{0}^{\left(1\right)}$	$\beta_{1}^{\left(1\right)}$	$\beta_{2}^{\left(1\right)}$	$\beta_{0}^{\left(2\right)}$	$\beta_{1}^{\left(2\right)}$	$\beta_{2}^{\left(2\right)}$
MLE	8.72	8.89	8.36	8.61	0.94	9.74	8.94	0.96	8.87	5.05
LS	16.98	19.39	14.44	15.92	1.98	19.09	15.63	2.11	18.42	13.99
QR${}_{0.25}$	17.62	20.01	14.98	16.92	2.11	20.38	16.11	2.26	19.99	15.13
QR${}_{0.75}$	17.69	19.92	14.87	16.57	2.22	20.97	16.04	2.31	20.08	15.24
ER${}_{0.25}$	17.59	20.02	15.07	16.91	2.07	20.46	16.01	2.37	20.13	15.72
ER${}_{0.75}$	18.01	20.43	15.16	16.13	2.12	20.71	16.07	2.41	20.34	15.83
WCER	12.56	12.48	9.06	9.52	1.05	10.67	9.37	1.12	9.62	6.26
	n=800									
**Estimate**	$\alpha_{1}^{\left(1\right)}$	$\alpha_{2}^{\left(1\right)}$	$\alpha_{1}^{\left(2\right)}$	$\alpha_{2}^{\left(2\right)}$	$\beta_{0}^{\left(1\right)}$	$\beta_{1}^{\left(1\right)}$	$\beta_{2}^{\left(1\right)}$	$\beta_{0}^{\left(2\right)}$	$\beta_{1}^{\left(2\right)}$	$\beta_{2}^{\left(2\right)}$
MLE	6.13	6.16	5.33	6.02	0.39	7.36	6.89	0.63	3.83	3.16
LS	12.30	13.87	10.39	11.99	1.34	13.82	12.03	1.51	11.87	10.01
QR${}_{0.25}$	12.41	13.90	10.98	12.14	1.45	14.72	12.58	1.72	12.61	10.99
QR${}_{0.75}$	12.47	13.99	11.13	12.31	1.49	14.70	12.64	1.86	12.59	11.07
ER${}_{0.25}$	12.43	13.98	11.17	12.51	1.47	14.62	12.63	1.68	12.56	11.09
ER${}_{0.75}$	12.69	13.78	11.23	12.63	1.58	14.67	12.72	1.90	12.49	11.03
WCER	7.22	8.11	5.64	7.31	0.57	8.16	7.87	0.78	4.53	3.92
	n=1500									
**Estimate**	$\alpha_{1}^{\left(1\right)}$	$\alpha_{2}^{\left(1\right)}$	$\alpha_{1}^{\left(2\right)}$	$\alpha_{2}^{\left(2\right)}$	$\beta_{0}^{\left(1\right)}$	$\beta_{1}^{\left(1\right)}$	$\beta_{2}^{\left(1\right)}$	$\beta_{0}^{\left(2\right)}$	$\beta_{1}^{\left(2\right)}$	$\beta_{2}^{\left(2\right)}$
MLE	4.27	5.13	3.02	4.01	0.23	5.65	3.38	0.40	1.98	1.95
LS	8.80	9.96	7.60	7.93	0.99	10.54	9.33	0.94	8.98	7.56
QR${}_{0.25}$	9.33	10.43	7.87	8.13	1.01	10.98	10.11	1.05	9.38	7.91
QR${}_{0.75}$	9.30	10.58	7.82	8.17	1.02	10.93	10.07	1.08	9.45	7.87
ER${}_{0.25}$	9.40	10.70	7.98	8.09	1.03	11.18	10.06	1.03	9.97	7.85
ER${}_{0.75}$	9.45	10.78	7.78	8.11	1.01	10.99	10.19	1.07	10.01	7.86
WCER	5.11	5.89	3.67	4.27	0.39	6.74	4.19	0.59	2.92	2.59
	n=2500									
**Estimate**	$\alpha_{1}^{\left(1\right)}$	$\alpha_{2}^{\left(1\right)}$	$\alpha_{1}^{\left(2\right)}$	$\alpha_{2}^{\left(2\right)}$	$\beta_{0}^{\left(1\right)}$	$\beta_{1}^{\left(1\right)}$	$\beta_{2}^{\left(1\right)}$	$\beta_{0}^{\left(2\right)}$	$\beta_{1}^{\left(2\right)}$	$\beta_{2}^{\left(2\right)}$
MLE	2.32	3.07	1.44	1.78	0.12	3.96	1.92	0.18	1.09	0.96
LS	4.63	5.81	3.96	4.39	0.44	7.01	5.39	0.56	5.68	4.15
QR${}_{0.25}$	4.72	5.87	4.04	4.41	0.47	7.16	5.42	0.59	5.73	4.23
QR${}_{0.75}$	4.68	5.89	4.09	4.45	0.49	7.19	5.45	0.57	5.75	4.27
ER${}_{0.25}$	4.76	5.92	4.06	4.40	0.46	7.21	5.41	0.58	5.71	4.21
ER${}_{0.75}$	4.78	5.90	4.08	4.47	0.47	7.20	5.44	0.60	5.72	4.25
WCER	2.49	3.29	1.56	1.89	0.21	4.15	2.17	0.28	1.27	1.15

Notes: All RMSEs are multiplied by $10^{2}$.

**Table 3:** RMSE comparison of various estimation methods, $\epsilon_{t}∼\chi^{2}\left(4\right)$.

	n=100									
Estimate	$\alpha_{1}^{\left(1\right)}$	$\alpha_{2}^{\left(1\right)}$	$\alpha_{1}^{\left(2\right)}$	$\alpha_{2}^{\left(2\right)}$	$\beta_{0}^{\left(1\right)}$	$\beta_{1}^{\left(1\right)}$	$\beta_{2}^{\left(1\right)}$	$\beta_{0}^{\left(2\right)}$	$\beta_{1}^{\left(2\right)}$	$\beta_{2}^{\left(2\right)}$
MLE	11.32	11.06	10.09	9.85	1.59	17.53	13.27	1.82	13.58	11.03
LS	20.56	23.45	19.82	20.89	3.17	27.96	24.38	3.95	22.50	21.94
QR${}_{0.25}$	20.11	23.24	19.59	20.52	3.03	27.02	24.11	3.91	22.43	21.87
QR${}_{0.75}$	20.52	23.37	19.81	20.78	3.11	27.21	24.10	3.98	22.52	21.99
ER${}_{0.25}$	19.48	23.13	19.41	20.19	2.99	26.87	24.07	3.80	22.37	20.20
ER${}_{0.75}$	20.78	23.58	19.98	21.04	3.24	28.01	24.89	3.99	22.63	22.06
WCER	18.34	19.64	15.57	15.99	1.97	20.16	14.13	2.52	16.59	13.09
	n=300									
**Estimate**	$\alpha_{1}^{\left(1\right)}$	$\alpha_{2}^{\left(1\right)}$	$\alpha_{1}^{\left(2\right)}$	$\alpha_{2}^{\left(2\right)}$	$\beta_{0}^{\left(1\right)}$	$\beta_{1}^{\left(1\right)}$	$\beta_{2}^{\left(1\right)}$	$\beta_{0}^{\left(2\right)}$	$\beta_{1}^{\left(2\right)}$	$\beta_{2}^{\left(2\right)}$
MLE	8.24	8.36	7.69	8.13	0.92	10.33	8.85	0.95	9.48	7.41
LS	17.83	19.45	16.05	16.53	2.02	19.32	17.45	2.30	19.63	16.71
QR${}_{0.25}$	17.49	19.10	16.07	16.31	2.01	19.15	17.31	2.22	19.47	16.56
QR${}_{0.75}$	17.58	19.21	16.02	16.43	2.07	19.20	17.41	2.28	19.58	16.61
ER${}_{0.25}$	17.35	18.98	15.88	16.13	1.92	18.98	17.24	2.15	19.38	16.40
ER${}_{0.75}$	17.98	19.48	16.13	16.75	2.11	19.60	17.58	2.38	19.75	16.82
WCER	12.24	12.71	10.17	10.09	1.09	14.04	10.89	1.17	10.29	10.57
	n=800									
**Estimate**	$\alpha_{1}^{\left(1\right)}$	$\alpha_{2}^{\left(1\right)}$	$\alpha_{1}^{\left(2\right)}$	$\alpha_{2}^{\left(2\right)}$	$\beta_{0}^{\left(1\right)}$	$\beta_{1}^{\left(1\right)}$	$\beta_{2}^{\left(1\right)}$	$\beta_{0}^{\left(2\right)}$	$\beta_{1}^{\left(2\right)}$	$\beta_{2}^{\left(2\right)}$
MLE	6.11	6.24	5.32	5.49	0.48	7.23	6.67	0.71	4.81	3.71
LS	13.66	14.52	11.65	13.03	1.77	15.15	13.21	1.81	15.12	12.01
QR${}_{0.25}$	13.28	14.47	11.21	12.78	1.68	15.08	13.12	1.80	14.98	11.99
QR${}_{0.75}$	13.42	14.58	11.35	12.92	1.79	15.19	13.23	1.87	15.07	12.03
ER${}_{0.25}$	12.96	14.35	10.99	12.63	1.59	14.98	12.97	1.74	14.87	11.75
ER${}_{0.75}$	13.72	14.63	11.74	13.18	1.79	15.26	13.26	1.86	15.24	12.09
WCER	7.06	7.88	6.39	7.17	0.64	8.92	7.51	0.82	5.12	4.51
	n=1500									
**Estimate**	$\alpha_{1}^{\left(1\right)}$	$\alpha_{2}^{\left(1\right)}$	$\alpha_{1}^{\left(2\right)}$	$\alpha_{2}^{\left(2\right)}$	$\beta_{0}^{\left(1\right)}$	$\beta_{1}^{\left(1\right)}$	$\beta_{2}^{\left(1\right)}$	$\beta_{0}^{\left(2\right)}$	$\beta_{1}^{\left(2\right)}$	$\beta_{2}^{\left(2\right)}$
MLE	3.75	3.52	3.11	3.68	0.29	5.64	3.17	0.47	2.91	1.87
LS	9.97	9.33	8.77	8.65	1.12	11.14	10.23	1.28	10.09	9.26
QR${}_{0.25}$	9.25	9.28	8.54	8.33	1.11	11.10	10.11	1.21	10.07	9.20
QR${}_{0.75}$	9.43	9.37	8.76	8.59	1.17	11.19	10.21	1.30	10.11	9.27
ER${}_{0.25}$	8.99	9.08	7.94	8.17	1.06	10.19	9.96	1.19	9.97	9.14
ER${}_{0.75}$	10.02	9.49	8.89	8.78	1.23	11.19	10.31	1.31	10.14	9.33
WCER	4.98	4.69	3.75	4.08	0.37	6.91	4.51	0.61	3.28	2.71
	n=2500									
**Estimate**	$\alpha_{1}^{\left(1\right)}$	$\alpha_{2}^{\left(1\right)}$	$\alpha_{1}^{\left(2\right)}$	$\alpha_{2}^{\left(2\right)}$	$\beta_{0}^{\left(1\right)}$	$\beta_{1}^{\left(1\right)}$	$\beta_{2}^{\left(1\right)}$	$\beta_{0}^{\left(2\right)}$	$\beta_{1}^{\left(2\right)}$	$\beta_{2}^{\left(2\right)}$
MLE	2.27	2.69	1.56	1.84	0.11	3.82	1.89	0.17	1.03	0.94
LS	4.68	5.78	4.01	4.42	0.47	7.08	5.42	0.55	5.72	4.21
QR${}_{0.25}$	4.77	5.89	4.12	4.50	0.49	7.19	5.49	0.57	5.83	4.30
QR${}_{0.75}$	4.74	5.92	4.14	4.48	0.48	7.23	5.51	0.59	5.85	4.28
ER${}_{0.25}$	4.76	5.98	4.15	4.44	0.51	7.25	5.55	0.56	5.81	4.27
ER${}_{0.75}$	4.78	5.96	4.13	4.47	0.54	7.21	5.50	0.60	5.87	4.26
WCER	2.41	2.98	1.76	1.93	0.22	3.99	2.05	0.27	1.19	1.08

Notes: All RMSEs are multiplied by $10^{2}$.

**Table 4:** Estimates of parameters for HSI.

Estimates	LS	QR${}_{0.25}$	QR${}_{0.75}$	ER${}_{0.25}$	ER${}_{0.75}$	WCER
$\alpha_{1}^{\left(1\right)}$	−0.1199 (0.18)	−0.1212 (0.23)	−0.1124 (0.19)	−0.1293 (0.20)	−0.1237 (0.19)	−0.1279 (0.08)
$\alpha_{2}^{\left(1\right)}$	−0.0909 (0.21)	−0.0921 (0.20)	−0.0914 (0.19)	−0.0937 (0.23)	−0.0955 (0.22)	−0.972 (0.08)
$\alpha_{1}^{\left(2\right)}$	−0.0424 (0.23)	−0.0492 (0.25)	−0.0433 (0.21)	−0.0481 (0.23)	−0.0475 (0.23)	−0.0487 (0.07)
$\alpha_{2}^{\left(2\right)}$	−0.0360 (0.15)	−0.0345 (0.15)	−0.0379 (0.14)	−0.0388 (0.17)	−0.0375 (0.16)	−0.0372 (0.04)
$\beta_{0}^{\left(1\right)}$	0.0059 (0.07)	0.0059 (0.08)	0.0062 (0.08)	0.0061 (0.07)	0.0058 (0.09)	0.0061 (0.05)
$\beta_{1}^{\left(1\right)}$	0.1798 (0.85)	0.1759 (0.79)	0.1694 (0.88)	0.1707 (0.84)	0.1763 (0.80)	0.1748 (0.47)
$\beta_{2}^{\left(1\right)}$	0.2901 (0.50)	0.2892 (0.48)	0.2859 (0.41)	0.2866 (0.44)	0.2873 (0.43)	0.2817 (0.22)
$\beta_{3}^{\left(1\right)}$	0.1837 (1.11)	0.1861 (1.14)	0.1790 (1.09)	0.1811 (1.17)	0.1843 (1.01)	0.1893 (0.89)
$\beta_{4}^{\left(1\right)}$	0.1298 (0.60)	0.1305 (0.63)	0.1321 (0.58)	0.1313 (0.55)	0.1355 (0.61)	0.1349 (0.21)
$\beta_{0}^{\left(2\right)}$	0.0077 (0.05)	0.0079 (0.06)	0.0074 (0.05)	0.0076 (0.07)	0.0078 (0.05)	0.0077 (0.04)
$\beta_{1}^{\left(2\right)}$	0.2877 (0.60)	0.2841 (0.62)	0.2763 (0.58)	0.2788 (0.64)	0.2859 (0.57)	0.2856 (0.34)
$\beta_{2}^{\left(2\right)}$	0.2134 (0.99)	0.2209 (0.89)	0.2098 (0.82)	0.2177 (0.86)	0.2106 (0.84)	0.2081 (0.68)
$\beta_{3}^{\left(2\right)}$	0.1298 (1.54)	0.1343 (1.62)	0.1266 (1.49)	0.1331 (1.57)	0.1276 (1.44)	0.1315 (1.19)
$\beta_{4}^{\left(2\right)}$	0.1923 (1.21)	0.1904 (1.30)	0.1898 (1.26)	0.1867 (1.19)	0.1854 (1.12)	0.1834 (0.93)

Note: Estimated SEs are given in parentheses, and all SEs are multiplied by $10^{2}$.

**Table 5:** Estimates of parameters for SPI.

Estimates	LS	QR${}_{0.25}$	QR${}_{0.75}$	ER${}_{0.25}$	ER${}_{0.75}$	WCER
$\alpha_{1}^{\left(1\right)}$	−0.0840 (0.18)	−0.0919 (0.21)	−0.0850 (0.22)	−0.0976 (0.17)	−0.0938 (0.17)	−0.0963 (0.09)
$\alpha_{2}^{\left(1\right)}$	−0.0750 (0.23)	−0.0736 (0.18)	−0.0709 (0.18)	−0.0769 (0.25)	−0.0801 (0.24)	−0.0798 (0.07)
$\alpha_{1}^{\left(2\right)}$	−0.0385 (0.24)	−0.0395 (0.21)	−0.0392 (0.19)	−0.0403 (0.22)	−0.0359 (0.25)	−0.0361 (0.07)
$\alpha_{2}^{\left(2\right)}$	−0.0257 (0.13)	−0.0214 (0.17)	−0.0290 (0.14)	−0.0276 (0.15)	−0.0265 (0.14)	−0.0282 (0.05)
$\beta_{0}^{\left(1\right)}$	0.0037 (0.09)	0.0038 (0.08)	0.0038 (0.08)	0.0039 (0.09)	0.0039 (0.09)	0.0039 (0.04)
$\beta_{1}^{\left(1\right)}$	0.1635 (0.69)	0.1634 (0.76)	0.1692 (0.72)	0.1609 (0.68)	0.1628 (0.73)	0.1596 (0.31)
$\beta_{2}^{\left(1\right)}$	0.2697 (0.54)	0.2614 (0.52)	0.2527 (0.49)	0.2496 (0.51)	0.2469 (0.55)	0.2445 (0.29)
$\beta_{3}^{\left(1\right)}$	0.1965 (1.24)	0.1912 (1.19)	0.1958 (1.31)	0.1892 (1.22)	0.1881 (1.25)	0.1786 (0.98)
$\beta_{4}^{\left(1\right)}$	0.1011 (0.41)	0.1280 (0.32)	0.1222 (0.37)	0.1173 (0.43)	0.1123 (0.35)	0.1094 (0.14)
$\beta_{0}^{\left(2\right)}$	0.0045 (0.10)	0.0044 (0.12)	0.0044 (0.11)	0.0046 (0.11)	0.0046 (0.11)	0.0045 (0.05)
$\beta_{1}^{\left(2\right)}$	0.2389 (0.68)	0.2347 (0.73)	0.2372 (0.70)	0.2452 (0.76)	0.2410 (0.81)	0.2433 (0.43)
$\beta_{2}^{\left(2\right)}$	0.1754 (0.78)	0.1731 (0.72)	0.1801 (0.75)	0.1780 (0.69)	0.1799 (0.77)	0.1823 (0.45)
$\beta_{3}^{\left(2\right)}$	0.1125 (1.43)	0.1083 (1.58)	0.1136 (1.61)	0.1101 (1.51)	0.1098 (1.47)	0.1037 (1.01)
$\beta_{4}^{\left(2\right)}$	0.1593 (1.09)	0.1436 (1.12)	0.1449 (1.19)	0.1498 (1.02)	0.1473 (1.11)	0.1505 (0.82)

Note: Estimated SEs are given in parentheses, and all SEs are multiplied by $10^{2}$.

**Table 6:** Comparing the fitted values and predicted values of HSI using MAPE.

Estimates	LS	QR${}_{0.25}$	QR${}_{0.75}$	ER${}_{0.25}$	ER${}_{0.75}$	WCER
$n_{2}=10$	0.0178	0.0181	0.0185	0.0174	0.0187	0.0120
$n_{2}=20$	0.0223	0.0234	0.0239	0.0219	0.0239	0.0157
$n_{2}=30$	0.0252	0.0268	0.0271	0.0248	0.0273	0.0186

**Table 7:** Comparing the fitted values and predicted values of SPI using MAPE.

Estimates	LS	QR${}_{0.25}$	QR${}_{0.75}$	ER${}_{0.25}$	ER${}_{0.75}$	WCER
$n_{2}=10$	0.0171	0.0176	0.0174	0.0176	0.0179	0.0105
$n_{2}=20$	0.0214	0.0223	0.0219	0.0222	0.0229	0.0144
$n_{2}=30$	0.0249	0.0256	0.0252	0.0258	0.0261	0.0179

## Data Availability

Not applicable.

## Supplementary Materials

The following supporting information can be downloaded at: https://www.mdpi.com/article/10.3390/e25081204/s1, Table S1: RMSE comparison of various estimation methods, $\epsilon_{t}∼N\left(0,1\right)$; Table S2: RMSE comparison of various estimation methods, $\epsilon_{t}∼t\left(6\right)$; Table S3: RMSE comparison of various estimation methods, $\epsilon_{t}∼\chi^{2}\left(4\right)$; Table S4: Parameter estimate of various estimation methods, $\epsilon_{t}∼N\left(0,1\right)$; Table S5: Parameter estimate of various estimation methods, $\epsilon_{t}∼t\left(6\right)$; Table S6: Parameter estimate of various estimation methods, $\epsilon_{t}∼\chi^{2}\left(4\right)$.

## Abbreviations

The following abbreviations are used in this manuscript:

MLE	Maximum Likelihood Estimation
LS	Least Squares
ER	Expectile Regression
QR	Quantile Regression
CER	Composite Expectile Regression
WCER	Weighted Composite Expectile Regression
EVaR	Expectile-based Value at Risk
DTARCH	Double-threshold Autoregressive Conditional Heteroscedastic
RMSE	Root Mean Squared Error

## Appendix A

This appendix contains proofs of the various theorems and corollaries used herein. The proofs of Theorem 1, Corollary 1 and Theorem 2 are similar to those of [42,43], which will not be listed in detail here. Theorem 3 is a special case when k=1 and ω=1 in Theorem 4, and hence its proof is omitted. Corollary 2 can be obtained in the same way as Theorem 4. Thus, we only give the detailed proof of Theorem 4, in which the following lemmas are needed.

**Lemma** **A1.** *Suppose that the conditions (C2), (C4) and (C5) hold. Then,*

$$\ell_{n}\left(v,\delta ,u\right)=\sum_{k=1}^{K}\omega_{k}\sum_{t=s+1}^{n}\left\{Q_{\tau_{k}}\left(S_{tk}^{*}-\Delta_{tk}\right)-Q_{\tau_{k}}\left(S_{tk}^{*}\right)\right\},$$

*which is defined in (A11), can be written as*

$$\ell_{n}\left(v,\delta ,u\right)=L_{n}^{⊤}\theta +\theta^{⊤}G\theta +o_{p}\left(1\right),$$

 
*where* $\theta ={\left(v^{⊤},\delta^{⊤},u^{⊤}\right)}^{⊤}$*;* $L_{n}={\left(L_{n1}^{⊤},L_{n2}^{⊤},L_{n3}^{⊤}\right)}^{⊤}$*, with*

$$\begin{array}{c} L_{n1}={\left(\begin{array}{c} \omega_{1}q_{n,1},\cdots ,\omega_{K}q_{n,K} \end{array}\right)}^{⊤},\;q_{n,k}=-\frac{1}{\sqrt{n}}\sum_{t=s+1}^{n}{\dot{Q}}_{\tau_{k}}\left(e_{t}-c_{\tau_{k}}^{*}\right)\left(k=1,\cdots ,K\right),\\ L_{n2}=-\frac{1}{\sqrt{n}}\sum_{k=1}^{K}\omega_{k}\sum_{t=s+1}^{n}{\dot{Q}}_{\tau_{k}}\left(e_{t}-c_{\tau_{k}}^{*}\right)r_{t},\\ L_{n3}=-\frac{1}{\sqrt{n}}\sum_{k=1}^{K}\omega_{k}\sum_{t=s+1}^{n}{\dot{Q}}_{\tau_{k}}\left(e_{t}-c_{\tau_{k}}^{*}\right)\frac{z_{t}}{h_{t}}; \end{array}$$

*$G=\left(\begin{array}{cc} G_{11} & G_{12}\\ G_{21} & G_{22} \end{array}\right)$ is a block matrix with each block being*

$$G_{11}=\mathrm{diag}\left(\omega_{1}\left[\left(1-2\tau_{1}\right)G\left(c_{\tau_{1}}^{*}\right)+\tau_{1}\right],\cdots ,\omega_{K}\left[\left(1-2\tau_{K}\right)G\left(c_{\tau_{K}}^{*}\right)+\tau_{K}\right]\right),$$

$$G_{12}=\left[G_{11}1\right]⊗\mu^{⊤},\;G_{21}=\left[1^{⊤}G_{11}\right]⊗\mu ,\;G_{22}=1^{⊤}G_{11}1\Omega .$$

**Proof** **of** **Lemma** **A1**. To facilitate the proof, we denote by

$$H_{t}=\left(\begin{array}{cc} H_{t11} & H_{t12}\\ H_{t21} & H_{t22} \end{array}\right),$$

where $H_{t}={\left.-\frac{\partial^{2}\log\left\{h_{t}\left(\alpha ,\beta \right)\right\}}{\partial \gamma \partial \gamma^{⊤}}\right|}_{\gamma^{*}}$ is a 2×2 block matrix with $\gamma =\left(\begin{array}{c} \alpha \\ \beta \end{array}\right)$, $\gamma^{*}=\left(\begin{array}{c} \alpha^{*}\\ \beta^{*} \end{array}\right)$, and

$$H=EH_{t}=\left(\begin{array}{cc} H_{11} & H_{12}\\ H_{21} & H_{22} \end{array}\right).$$

Note that for arbitrary positive number *a*, we have

$$P\left(\left|\frac{n^{-1/2}x_{t}^{⊤}\delta }{\epsilon_{t}}\right|<a\right)\to 1.$$

Then, it follows from Taylor’s expansion for the natural logarithm $\log|\epsilon_{t}\left(\alpha \right)|$ that

(A1) $$\begin{array}{c} \log|\epsilon_{t}\left(\alpha \right)|-\log|\epsilon_{t}\left(\alpha^{*}\right)|=-\frac{x_{t}^{⊤}\delta }{\sqrt{n}\epsilon_{t}}-\frac{1}{2n}\frac{\delta^{⊤}x_{t}x_{t}^{⊤}\delta }{\epsilon_{t}^{2}}+o_{p}\left(n^{-1}\right). \end{array}$$

And by Taylor’s expansions for the natural logarithm $\log\left\{h_{t}\left(\alpha ,\beta \right)\right\}$, we have

(A2) $$\begin{array}{cc} & \log\left\{h_{t}\left(\alpha ,\beta \right)\right\}-\log\left\{h_{t}\left(\alpha^{*},\beta^{*}\right)\right\}\\ = & \frac{f_{t}^{⊤}\delta }{\sqrt{n}h_{t}}+\frac{z_{t}^{⊤}u}{\sqrt{n}h_{t}}-\frac{1}{2n}\delta^{⊤}H_{t11}\delta -\frac{1}{2n}u^{⊤}H_{t22}u-\frac{1}{n}\delta^{⊤}H_{t12}u+o_{p}\left(n^{-1}\right), \end{array}$$

where

$$f_{t}=-\sum_{j=1}^{m}I_{t,j}\left(0,\mathrm{sgn}\left(\epsilon_{t-1}\right)x_{t-1},\cdots ,\mathrm{sgn}\left(\epsilon_{t-q_{j}}\right)x_{t-q_{j}}\right)\beta^{\left(j\right)}.$$

By substituting Equations (A1) and (A2) into $\Delta_{tk}$, where the definition of $\Delta_{tk}$ is given in the proof of Theorem 4, we can obtain that

(A3) $$\begin{array}{c} \begin{array}{cc} \Delta_{tk} & =\frac{v_{k}}{\sqrt{n}}+\frac{z_{t}^{⊤}u}{\sqrt{n}h_{t}}+\frac{r_{t}^{⊤}\delta }{\sqrt{n}}+\frac{1}{2n}\frac{\delta^{⊤}x_{t}x_{t}^{⊤}\delta }{\epsilon_{t}^{2}}\\ & \;\;\;\;\;\;-\frac{1}{2n}\delta^{⊤}H_{t11}\delta -\frac{1}{2n}u^{⊤}H_{t22}u-\frac{1}{n}\delta^{⊤}H_{t12}u+o_{p}\left(n^{-1}\right), \end{array} \end{array}$$

where $r_{t}=\frac{x_{t}}{\epsilon_{t}}+\frac{f_{t}}{h_{t}}$. Note that $\ell_{n}\left(v,\delta ,u\right)$ is convex in ${\left(v^{⊤},\delta^{⊤},u^{⊤}\right)}^{⊤}$. Showing that $\ell_{n}\left(v,\delta ,u\right)$ converges pointwise to its conditional expectation is enough, and the convergence is uniformly valid on any compact set of ${\left(v^{⊤},\delta^{⊤},u^{⊤}\right)}^{⊤}$, which is because of the convexity lemma in [53].

(A4) $$\begin{array}{c} \begin{array}{cc} & \;\;\;\;\ell_{n}\left(v,\delta ,u\right)\\ & =\sum_{k=1}^{K}\omega_{k}\sum_{t=s+1}^{n}E\left\{\left.Q_{\tau_{k}}\left(S_{tk}^{*}-\Delta_{tk}\right)-Q_{\tau_{k}}\left(S_{tk}^{*}\right)+{\dot{Q}}_{\tau_{k}}\left(S_{tk}^{*}\right)\Delta_{tk}\right|x_{t},z_{t}\right\}\\ & \;\;\;\;-\sum_{k=1}^{K}\omega_{k}\sum_{t=s+1}^{n}{\dot{Q}}_{\tau_{k}}\left(S_{tk}^{*}\right)\Delta_{tk}+R_{n,k}\left(v,\delta ,u\right), \end{array} \end{array}$$

where

$$\begin{array}{cc} & \;\;\;\;Q_{\tau_{k}}\left(S_{tk}^{*}-\Delta_{tk}\right)-Q_{\tau_{k}}\left(S_{tk}^{*}\right)+{\dot{Q}}_{\tau_{k}}\left(S_{tk}^{*}\right)\Delta_{tk}\\ & =\left(1-2\tau_{k}\right){\left[S_{tk}^{*}-\Delta_{tk}\right]}^{2}\left[I\left(S_{tk}^{*}\leq \Delta_{tk}\right)-I\left(S_{tk}^{*}\leq 0\right)\right]+\left(1-\tau_{k}\right)\Delta_{tk}^{2}I\left(S_{tk}^{*}\leq 0\right)+\tau_{k}\Delta_{tk}^{2}I\left(S_{tk}^{*}>0\right), \end{array}$$

and

$$E\left\{\left.\left[I\left(S_{tk}^{*}<\Delta_{tk}\right)-I\left(S_{tk}^{*}<0\right)\right]\right|x_{t},z_{t}\right\}=g\left(c_{\tau_{k}}^{*}\right)\Delta_{tk}+\frac{1}{2}\dot{g}\left(c_{\tau_{k}}^{*}\right)\Delta_{tk}^{2}+o\left(\Delta_{tk}^{2}\right),$$

$$E\left\{\left.e_{t}\left[I\left(S_{tk}^{*}<\Delta_{tk}\right)-I\left(S_{tk}^{*}<0\right)\right]\right|x_{t},z_{t}\right\}=c_{\tau_{k}}^{*}g\left(c_{\tau_{k}}^{*}\right)\Delta_{tk}+\frac{1}{2}\left[g\left(c_{\tau_{k}}^{*}\right)+c_{\tau_{k}}^{*}\dot{g}\left(c_{\tau_{k}}^{*}\right)\right]\Delta_{tk}^{2}+o\left(\Delta_{tk}^{2}\right),$$

$$\begin{array}{cc} & \;\;\;\;E\left\{\left.e_{t}^{2}\left[I\left(S_{tk}^{*}<\Delta_{tk}\right)-I\left(S_{tk}^{*}<0\right)\right]\right|x_{t},z_{t}\right\}\\ & ={\left(c_{\tau_{k}}^{*}\right)}^{2}g\left(c_{\tau_{k}}^{*}\right)\Delta_{tk}+\frac{1}{2}\left[2c_{\tau_{k}}^{*}g\left(c_{\tau_{k}}^{*}\right)+{\left(c_{\tau_{k}}^{*}\right)}^{2}\dot{g}\left(c_{\tau_{k}}^{*}\right)\right]\Delta_{tk}^{2}+o\left(\Delta_{tk}^{2}\right). \end{array}$$

From the above equations, we can obtain that

(A5) $$\begin{array}{c} \begin{array}{cc} & \;\;\;\;\sum_{k=1}^{K}\omega_{k}\sum_{t=s+1}^{n}E\left\{\left.Q_{\tau_{k}}\left(S_{tk}^{*}-\Delta_{tk}\right)-Q_{\tau_{k}}\left(S_{tk}^{*}\right)+{\dot{Q}}_{\tau_{k}}\left(S_{tk}^{*}\right)\Delta_{tk}\right|x_{t},z_{t}\right\}\\ & =\sum_{k=1}^{K}\omega_{k}\left[\left(1-2\tau_{k}\right)G\left(c_{\tau_{k}}^{*}\right)+\tau_{k}\right]\left(v_{k},\delta^{⊤},u^{⊤}\right)\\ & \;\;\;\;\;\;\;\;\times \left(\begin{array}{ccc} \frac{1}{n}\sum_{t=s+1}^{n}1 & \frac{1}{n}\sum_{t=s+1}^{n}r_{t}^{⊤} & \frac{1}{n}\sum_{t=s+1}^{n}\frac{z_{t}^{⊤}}{h_{t}}\\ \frac{1}{n}\sum_{t=s+1}^{n}r_{t} & \frac{1}{n}\sum_{t=s+1}^{n}r_{t}r_{t}^{⊤} & \frac{1}{n}\sum_{t=s+1}^{n}\frac{r_{t}z_{t}^{⊤}}{h_{t}}\\ \frac{1}{n}\sum_{t=s+1}^{n}\frac{z_{t}}{h_{t}} & \frac{1}{n}\sum_{t=s+1}^{n}\frac{z_{t}r_{t}^{⊤}}{h_{t}} & \frac{1}{n}\sum_{t=s+1}^{n}\frac{z_{t}z_{t}^{⊤}}{h_{t}^{2}} \end{array}\right)\left(\begin{array}{c} v_{k}\\ \delta \\ u \end{array}\right)+O_{p}\left(\frac{1}{\sqrt{n}}\right). \end{array} \end{array}$$

By the Chebyshev’s weak law of large numbers, we obtain that

$$\left(\begin{array}{ccc} \frac{1}{n}\sum_{t=s+1}^{n}1 & \frac{1}{n}\sum_{t=s+1}^{n}r_{t}^{⊤} & \frac{1}{n}\sum_{t=s+1}^{n}\frac{z_{t}^{⊤}}{h_{t}}\\ \frac{1}{n}\sum_{t=s+1}^{n}r_{t} & \frac{1}{n}\sum_{t=s+1}^{n}r_{t}r_{t}^{⊤} & \frac{1}{n}\sum_{t=s+1}^{n}\frac{r_{t}z_{t}^{⊤}}{h_{t}}\\ \frac{1}{n}\sum_{t=s+1}^{n}\frac{z_{t}}{h_{t}} & \frac{1}{n}\sum_{t=s+1}^{n}\frac{z_{t}r_{t}^{⊤}}{h_{t}} & \frac{1}{n}\sum_{t=s+1}^{n}\frac{z_{t}z_{t}^{⊤}}{h_{t}^{2}} \end{array}\right)\overset{\;p\;}{\to }\left(\begin{array}{ccc} 1 & \mu_{r}^{⊤} & \mu_{z}^{⊤}\\ \mu_{r} & \Omega_{r} & \Omega_{rz}^{⊤}\\ \mu_{z} & \Omega_{rz} & \Omega_{z} \end{array}\right)≜\Gamma .$$

By substituting the above equations into Equation (A4), we can obtain that

(A6) $$\begin{array}{c} \begin{array}{cc} & \;\;\;\;\ell_{n}\left(v,\delta ,u\right)\\ & =-\sum_{k=1}^{K}\omega_{k}v_{k}\frac{1}{\sqrt{n}}\sum_{t=s+1}^{n}{\dot{Q}}_{\tau_{k}}\left(e_{t}-c_{\tau_{k}}^{*}\right)-\sum_{k=1}^{K}\omega_{k}\frac{1}{\sqrt{n}}\sum_{t=s+1}^{n}{\dot{Q}}_{\tau_{k}}\left(e_{t}-c_{\tau_{k}}^{*}\right)r_{t}^{⊤}\delta \\ & \;\;\;\;-\sum_{k=1}^{K}\omega_{k}\frac{1}{\sqrt{n}}\sum_{t=s+1}^{n}{\dot{Q}}_{\tau_{k}}\left(e_{t}-c_{\tau_{k}}^{*}\right)\frac{z_{t}^{⊤}}{h_{t}}u\\ & \;\;\;\;+\sum_{k=1}^{K}\omega_{k}\delta^{⊤}\left[\frac{1}{n}\sum_{t=s+1}^{n}{\dot{Q}}_{\tau_{k}}\left(e_{t}-c_{\tau_{k}}^{*}\right)H_{t12}\right]u\\ & \;\;\;\;+\sum_{k=1}^{K}\omega_{k}u^{⊤}\left[\frac{1}{2n}\sum_{t=s+1}^{n}{\dot{Q}}_{\tau_{k}}\left(e_{t}-c_{\tau_{k}}^{*}\right)H_{t22}\right]u\\ & \;\;\;\;+\sum_{k=1}^{K}\omega_{k}\delta^{⊤}\left[\frac{1}{2n}\sum_{t=s+1}^{n}{\dot{Q}}_{\tau_{k}}\left(e_{t}-c_{\tau_{k}}^{*}\right)\left(H_{t11}-\frac{x_{t}x_{t}^{⊤}}{\epsilon_{t}^{2}}\right)\right]\delta \\ & \;\;\;\;+\sum_{k=1}^{K}\omega_{k}\left[\left(1-2\tau_{k}\right)G\left(c_{\tau_{k}}^{*}\right)+\tau_{k}\right]\theta_{k}^{⊤}\Gamma \theta_{k}+o_{p}\left(1\right)+R_{n,k}\left(v,\delta ,u\right), \end{array} \end{array}$$

where $\theta_{k}={\left(v_{k},\delta^{⊤},u^{⊤}\right)}^{⊤}$. Since $E\left[{\dot{Q}}_{\tau_{k}}\left(e_{t}-c_{\tau_{k}}^{*}\right)\right]=0$, by the Chebyshev’s weak law of large numbers, we obtain that

$$\frac{1}{n}\sum_{t=s+1}^{n}{\dot{Q}}_{\tau_{k}}\left(e_{t}-c_{\tau_{k}}^{*}\right)H_{t12}\overset{\;p\;}{\to }0,$$

$$\frac{1}{n}\sum_{t=s+1}^{n}{\dot{Q}}_{\tau_{k}}\left(e_{t}-c_{\tau_{k}}^{*}\right)H_{t22}\overset{\;p\;}{\to }0,$$

$$\frac{1}{n}\sum_{t=s+1}^{n}{\dot{Q}}_{\tau_{k}}\left(e_{t}-c_{\tau_{k}}^{*}\right)\left(H_{t11}-\frac{x_{t}x_{t}^{⊤}}{\epsilon_{t}^{2}}\right)\overset{\;p\;}{\to }0.$$

Subsequently, we obtain that

(A7) $$\begin{array}{c} \begin{array}{cc} & \;\;\;\;\ell_{n}\left(v,\delta ,u\right)\\ & =-\sum_{k=1}^{K}\omega_{k}\frac{1}{\sqrt{n}}\sum_{t=s+1}^{n}{\dot{Q}}_{\tau_{k}}\left(e_{t}-c_{\tau_{k}}^{*}\right)v_{k}\\ & \;\;\;\;-\sum_{k=1}^{K}\omega_{k}\frac{1}{\sqrt{n}}\sum_{t=s+1}^{n}{\dot{Q}}_{\tau_{k}}\left(e_{t}-c_{\tau_{k}}^{*}\right)r_{t}^{⊤}\delta \\ & \;\;\;\;-\sum_{k=1}^{K}\omega_{k}\frac{1}{\sqrt{n}}\sum_{t=s+1}^{n}{\dot{Q}}_{\tau_{k}}\left(e_{t}-c_{\tau_{k}}^{*}\right)\frac{z_{t}^{⊤}}{h_{t}}u\\ & \;\;\;\;+\sum_{k=1}^{K}\omega_{k}\left[\left(1-2\tau_{k}\right)G\left(c_{\tau_{k}}^{*}\right)+\tau_{k}\right]\theta_{k}^{⊤}\Gamma \theta_{k}+o_{p}\left(1\right)+R_{n,k}\left(v,\delta ,u\right). \end{array} \end{array}$$

According to Lemma A2, we can obtain that

$$R_{n,k}\left(v,\delta ,u\right)=o_{p}\left(n^{-1}\right)=o_{p}\left(1\right),$$

which combining with (A7) and performing some straightforward calculations, we arrive at the conclusion of Lemma A1. □

**Lemma** **A2.**
*Suppose that the conditions (C2), (C4) and (C5) hold. Then,*

$$R_{n,k}\left(v,\delta ,u\right)=o_{p}\left(n^{-1}\right)=o_{p}\left(1\right),$$

*which is defined in (A4).*

**Proof** **of** **Lemma** **A2**. We can manipulate Equation (A4) to obtain the following form

$$\begin{array}{c} \begin{array}{cc} & \;\;\;\;E\left\{\ell_{n}\left(v,\delta ,u\right)\right\}\\ & =E\left\{E\left\{\left.\ell_{n}\left(v,\delta ,u\right)\right|x,z\right\}\right\}-\left[E\left\{\sum_{k=1}^{K}\omega_{k}\sum_{t=s+1}^{n}{\dot{Q}}_{\tau_{k}}\left(S_{tk}^{*}\right)\Delta_{tk}\right\}\right.\\ & \;\;\;\;\;\;\;\;\;\;\left.-E\left\{\sum_{k=1}^{K}\omega_{k}\sum_{t=s+1}^{n}E\left\{\left.{\dot{Q}}_{\tau_{k}}\left(S_{tk}^{*}\right)\Delta_{tk}\right|x_{t},z_{t}\right\}\right\}\right]+E\left\{R_{n,k}\left(v,\delta ,u\right)\right\}, \end{array} \end{array}$$

which yields that

(A8) $$\begin{array}{c} E\left\{R_{n,k}\left(v,\delta ,u\right)\right\}=0. \end{array}$$

According to Equations (A6) and (A11), we obtain that

(A9) $$\begin{array}{c} \begin{array}{cc} R_{n,k}\left(v,\delta ,u\right) & =\sum_{k=1}^{K}\omega_{k}\sum_{t=s+1}^{n}\left\{Q_{\tau_{k}}\left(S_{tk}^{*}-\Delta_{tk}\right)-Q_{\tau_{k}}\left(S_{tk}^{*}\right)+{\dot{Q}}_{\tau_{k}}\left(S_{tk}^{*}\right)\Delta_{tk}\right\}\\ & \;\;\;\;-\sum_{k=1}^{K}\omega_{k}\left[\left(1-2\tau_{k}\right)G\left(c_{\tau_{k}}^{*}\right)+\tau_{k}\right]\theta_{k}^{⊤}\Gamma \theta_{k}+o_{p}\left(1\right). \end{array} \end{array}$$

By Lemma 2 in [9], we obtain that

$$\begin{array}{c} \begin{array}{cc} \mathrm{Var}\left\{R_{n,k}\left(v,\delta ,u\right)\right\} & =n\sum_{k=1}^{K}\omega_{k}\mathrm{Var}\left\{Q_{\tau_{k}}\left(S_{tk}^{*}-\Delta_{tk}\right)-Q_{\tau_{k}}\left(S_{tk}^{*}\right)+{\dot{Q}}_{\tau_{k}}\left(S_{tk}^{*}\right)\Delta_{tk}\right\}\\ & \leq n\sum_{k=1}^{K}\omega_{k}E{\left\{Q_{\tau_{k}}\left(S_{tk}^{*}-\Delta_{tk}\right)-Q_{\tau_{k}}\left(S_{tk}^{*}\right)+{\dot{Q}}_{\tau_{k}}\left(S_{tk}^{*}\right)\Delta_{tk}\right\}}^{2}\\ & \leq n\sum_{k=1}^{K}\omega_{k}E{\left(4\Delta_{tk}^{2}\right)}^{2}\\ & =O\left(n^{-1}\right), \end{array} \end{array}$$

i.e.,

(A10) $$\begin{array}{c} \mathrm{Var}\left\{R_{n,k}\left(v,\delta ,u\right)\right\}=o\left(n^{-1}\right). \end{array}$$

From (A8) and (A10), it follows that

$$R_{n,k}\left(v,\delta ,u\right)=o_{p}\left(n^{-1}\right)=o_{p}\left(1\right).$$

This completes the proof of Lemma A2. □

**Lemma** **A3.**
*Suppose that the conditions (C2), (C4) and (C5) hold. Then,*

$$L_{n}\overset{\;D\;}{\to }N\left(0,\Sigma^{0}\right),$$

*where $L_{n}$ is defined in Lemma A1, and $\Sigma^{0}=\left(\begin{array}{cc} \Sigma_{11}^{0} & \Sigma_{12}^{0}\\ \Sigma_{21}^{0} & \Sigma_{22}^{0} \end{array}\right)$ is a block matrix with blocks $\Sigma_{11}^{0}$ is a K×K matrix with the (j,k)th element being*

$$\Sigma_{11}^{0}\left(j,k\right)=\omega_{j}\omega_{k}E\left[{\dot{Q}}_{\tau_{j}}\left(e_{t}-c_{\tau_{j}}^{*}\right){\dot{Q}}_{\tau_{k}}\left(e_{t}-c_{\tau_{k}}^{*}\right)\right];$$

$$\Sigma_{12}^{0}=\left[\Sigma_{11}^{0}1\right]⊗\mu^{⊤};\;\Sigma_{21}^{0}=\left[1^{⊤}\Sigma_{11}^{0}\right]⊗\mu ;\;\Sigma_{22}^{0}=1^{⊤}\Sigma_{11}^{0}1\Omega .$$

**Proof** **of** **Lemma** **A3.** Note that

$$L_{n}=\left(\begin{array}{c} L_{n1}\\ L_{n2}\\ L_{n3} \end{array}\right)=\left(\begin{array}{c} -\frac{1}{\sqrt{n}}\sum_{t=s+1}^{n}{\dot{Q}}_{\tau_{1}}\left(e_{t}-c_{\tau_{1}}^{*}\right)\omega_{1}\\ \vdots \\ -\frac{1}{\sqrt{n}}\sum_{t=s+1}^{n}{\dot{Q}}_{\tau_{K}}\left(e_{t}-c_{\tau_{K}}^{*}\right)\omega_{K}\\ -\frac{1}{\sqrt{n}}\sum_{k=1}^{K}\omega_{k}\sum_{t=s+1}^{n}{\dot{Q}}_{\tau_{k}}\left(e_{t}-c_{\tau_{k}}^{*}\right)r_{t}\\ -\frac{1}{\sqrt{n}}\sum_{k=1}^{K}\omega_{k}\sum_{t=s+1}^{n}{\dot{Q}}_{\tau_{k}}\left(e_{t}-c_{\tau_{k}}^{*}\right)\frac{z_{t}}{h_{t}} \end{array}\right).$$

By the Cramer–Wald device and the Central Limit Theorem, we obtain that

$$\sqrt{n}\left(\frac{1}{\sqrt{n}}L_{n}-\mu_{L}\right)\overset{\;D\;}{\to }N\left(0_{(K+p+q)\times 1},\Sigma^{0}\right).$$

We now calculate $\mu_{L}$ and $\Sigma^{0}$, respectively. It is easy to show that

$$\mu_{L}=0_{(K+p+q)\times 1}.$$

Let

$$\Sigma^{0}=\left(\begin{array}{cc} \Sigma_{11}^{0} & \Sigma_{12}^{0}\\ \Sigma_{21}^{0} & \Sigma_{22}^{0} \end{array}\right),$$

where

$$\Sigma_{11}^{0}=E\left(\left(\begin{array}{c} -{\dot{Q}}_{\tau_{1}}\left(e_{t}-c_{\tau_{1}}^{*}\right)\omega_{1}\\ \vdots \\ -{\dot{Q}}_{\tau_{K}}\left(e_{t}-c_{\tau_{K}}^{*}\right)\omega_{K} \end{array}\right){\left(\begin{array}{c} -{\dot{Q}}_{\tau_{1}}\left(e_{t}-c_{\tau_{1}}^{*}\right)\omega_{1}\\ \vdots \\ -{\dot{Q}}_{\tau_{K}}\left(e_{t}-c_{\tau_{K}}^{*}\right)\omega_{K} \end{array}\right)}^{⊤}\right),$$

which is a K×K matrix with the (j,k)th element being

$$\begin{array}{cc} & \;\;\;\;\Sigma_{11}\left(j,k\right)=\omega_{j}\omega_{k}E\left({\dot{Q}}_{\tau_{j}}\left(e_{t}-c_{\tau_{j}}^{*}\right){\dot{Q}}_{\tau_{k}}\left(e_{t}-c_{\tau_{k}}^{*}\right)\right)\\ & =4\omega_{j}\omega_{k}\int \left|\tau_{j}\tau_{k}-\tau_{j}I\left(e_{t}\leq c_{\tau_{k}}^{*}\right)-\tau_{k}I\left(e_{t}\leq c_{\tau_{j}}^{*}\right)+I\left(e_{t}\leq c_{\tau_{j}}^{*}\wedge c_{\tau_{k}}^{*}\right)\right|\\ & \;\;\;\;\;\;\;\;\;\;\;\;\;\;\;\;\;\;\times \left[e_{t}^{2}-\left(c_{\tau_{j}}^{*}+c_{\tau_{k}}^{*}\right)e_{t}+c_{\tau_{j}}^{*}c_{\tau_{k}}^{*}\right]dG\left(e_{t}\right). \end{array}$$

We can obtain the expressions of $\Sigma_{12}$, $\Sigma_{21}$ and $\Sigma_{22}$ similarly. Thus,

$$L_{n}\overset{\;D\;}{\to }N\left(0,\Sigma^{0}\right),$$

which completes the proof of Lemma A3. □

**Proof** **of** **Theorem** **4.** Let $\sqrt{n}\left(\alpha -\alpha^{*}\right)=\delta$, $\sqrt{n}\left(\beta -\beta^{*}\right)=u$, $\sqrt{n}\left(c_{\tau_{k}}-c_{\tau_{k}}^{*}\right)=v_{k}$, $v={\left(v_{1},\cdots ,v_{K}\right)}^{⊤}$ and

$$\begin{array}{cc} S_{tk} & =\log|\epsilon_{t}\left(\alpha \right)|-\log\left\{h_{t}\left(\alpha ,\beta \right)\right\}-c_{\tau_{k}}\\ & =\log|\epsilon_{t}\left(\alpha^{*}+n^{-1/2}\delta \right)|-\log\left\{h_{t}\left(\alpha^{*}+n^{-1/2}\delta ,\beta^{*}+n^{-1/2}u\right)\right\}-\left(c_{\tau_{k}}+n^{-1/2}v_{k}\right). \end{array}$$

Define $S_{tk}^{*}=\log\left|\epsilon_{t}\left(\alpha^{*}\right)\right|-\log\left\{h_{t}\left(\alpha^{*},\beta^{*}\right)\right\}-c_{\tau_{k}}^{*}=e_{t}-c_{\tau_{k}}^{*}$, $\Delta_{tk}=S_{tk}^{*}-S_{tk}$, and

(A11) $$\begin{array}{c} \begin{array}{cc} \ell_{n}\left(v,\delta ,u\right) & =\sum_{k=1}^{K}\omega_{k}\sum_{t=s+1}^{n}\left\{Q_{\tau_{k}}\left(S_{tk}\right)-Q_{\tau_{k}}\left(e_{t}-c_{\tau_{k}}^{*}\right)\right\}\\ & =\sum_{k=1}^{K}\omega_{k}\sum_{t=s+1}^{n}\left\{Q_{\tau_{k}}\left(S_{tk}^{*}-\Delta_{tk}\right)-Q_{\tau_{k}}\left(S_{tk}^{*}\right)\right\}. \end{array} \end{array}$$

Then minimizing the objective in (9) is equivalent to minimizing $\ell_{n}\left(v,\delta ,u\right)$. By Lemma A1, we obtain that

(A12) $$\begin{array}{c} \ell_{n}\left(v,\delta ,u\right)=L_{n}^{⊤}\theta +\frac{1}{2}\theta^{⊤}\left(2G\right)\theta +o_{p}\left(1\right), \end{array}$$

where $\theta ={\left(v^{⊤},\delta^{⊤},u^{⊤}\right)}^{⊤}$; $L_{n}={\left(L_{n1}^{⊤},L_{n2}^{⊤},L_{n3}^{⊤}\right)}^{⊤}$, with

$$\begin{array}{cc} L_{n1} & ={\left(\begin{array}{c} \omega_{1}q_{n,1},\cdots ,\omega_{K}q_{n,K} \end{array}\right)}^{⊤},\;q_{n,k}=-\frac{1}{\sqrt{n}}\sum_{t=s+1}^{n}{\dot{Q}}_{\tau_{k}}\left(e_{t}-c_{\tau_{k}}^{*}\right)\left(k=1,\cdots ,K\right),\\ L_{n2} & =-\frac{1}{\sqrt{n}}\sum_{k=1}^{K}\omega_{k}\sum_{t=s+1}^{n}{\dot{Q}}_{\tau_{k}}\left(e_{t}-c_{\tau_{k}}^{*}\right)r_{t},\\ L_{n3} & =-\frac{1}{\sqrt{n}}\sum_{k=1}^{K}\omega_{k}\sum_{t=s+1}^{n}{\dot{Q}}_{\tau_{k}}\left(e_{t}-c_{\tau_{k}}^{*}\right)\frac{z_{t}}{h_{t}}; \end{array}$$

$G=\left(\begin{array}{cc} G_{11} & G_{12}\\ G_{21} & G_{22} \end{array}\right)$ is a block matrix, with each block being

$$G_{11}=\mathrm{diag}\left(\omega_{1}\left[\left(1-2\tau_{1}\right)G\left(c_{\tau_{1}}^{*}\right)+\tau_{1}\right],\cdots ,\omega_{K}\left[\left(1-2\tau_{K}\right)G\left(c_{\tau_{K}}^{*}\right)+\tau_{K}\right]\right),$$

$$G_{12}=\left[G_{11}1\right]⊗\mu^{⊤},\;G_{21}=\left[1^{⊤}G_{11}\right]⊗\mu ,\;G_{22}=1^{⊤}G_{11}1\Omega .$$

We further perform transformation calculations on matrix *G*, resulting in

$$\begin{array}{cc} & \;\;\;\;G=\left(\begin{array}{cc} G_{11} & \left[G_{11}1\right]⊗\mu^{⊤}\\ \left[1^{⊤}G_{11}\right]⊗\mu & 1^{⊤}G_{11}1\Omega \end{array}\right)\\ & =\left(\begin{array}{cc} G_{11}^{1/2} & 0\\ \left[1^{⊤}G_{11}^{1/2}\right]⊗\mu & I \end{array}\right)\left(\begin{array}{cc} I & 0\\ 0 & 1^{⊤}G_{11}1\Pi \end{array}\right)\left(\begin{array}{cc} G_{11}^{1/2} & \left[G_{11}^{1/2}1\right]⊗\mu^{⊤}\\ 0 & I \end{array}\right)\\ & =\left(\begin{array}{cc} G_{11}^{1/2} & 0\\ \left[1^{⊤}G_{11}^{1/2}\right]⊗\mu & I \end{array}\right){\left(\begin{array}{cc} I & 0\\ 0 & 1^{⊤}G_{11}1\Pi \end{array}\right)}^{1/2}{\left(\begin{array}{cc} I & 0\\ 0 & 1^{⊤}G_{11}1\Pi \end{array}\right)}^{1/2}\left(\begin{array}{cc} G_{11}^{1/2} & \left[G_{11}^{1/2}1\right]⊗\mu^{⊤}\\ 0 & I \end{array}\right)\\ & =\left[\left(\begin{array}{cc} G_{11}^{1/2} & 0\\ \left[1^{⊤}G_{11}^{1/2}\right]⊗\mu & I \end{array}\right){\left(\begin{array}{cc} I & 0\\ 0 & 1^{⊤}G_{11}1\Pi \end{array}\right)}^{1/2}\right]{\left[\left(\begin{array}{cc} G_{11}^{1/2} & 0\\ \left[1^{⊤}G_{11}^{1/2}\right]⊗\mu & I \end{array}\right){\left(\begin{array}{cc} I & 0\\ 0 & 1^{⊤}G_{11}1\Pi \end{array}\right)}^{1/2}\right]}^{⊤}, \end{array}$$

that is to say, *G* is a positive matrix. Furthermore, according to Lemma A3, we obtain that

$$L_{n}\overset{\;D\;}{\to }N\left(0,\Sigma^{0}\right),$$

where $\Sigma^{0}=\left(\begin{array}{cc} \Sigma_{11}^{0} & \Sigma_{12}^{0}\\ \Sigma_{21}^{0} & \Sigma_{22}^{0} \end{array}\right)$ is a block matrix, and each block is, respectively: $\Sigma_{11}^{0}$ is a K×K matrix with the (j,k)th element being

$$\Sigma_{11}^{0}\left(j,k\right)=\omega_{j}\omega_{k}E\left[{\dot{Q}}_{\tau_{j}}\left(e_{t}-c_{\tau_{j}}^{*}\right){\dot{Q}}_{\tau_{k}}\left(e_{t}-c_{\tau_{k}}^{*}\right)\right];$$

$$\Sigma_{12}^{0}=\left[\Sigma_{11}^{0}1\right]⊗\mu^{⊤};\;\Sigma_{21}^{0}=\left[1^{⊤}\Sigma_{11}^{0}\right]⊗\mu ;\;\Sigma_{22}^{0}=1^{⊤}\Sigma_{11}^{0}1\Omega .$$

Therefore, by Theorem 2 in [54], we can obtain that

$$\hat{\theta }\overset{\;D\;}{\to }N\left(0,\frac{1}{4}G^{-1}\Sigma^{0}G^{-1}\right).$$

Note $\hat{\theta }=\sqrt{n}{\left({\hat{c}}_{\tau_{1}}-c_{\tau_{1}}^{*},\cdots ,{\hat{c}}_{\tau_{K}}-c_{\tau_{K}}^{*},{\left(\hat{\alpha }-\alpha^{*}\right)}^{⊤},{\left(\hat{\beta }-\beta^{*}\right)}^{⊤}\right)}^{⊤}$. Now, we obtain that

$$\sqrt{n}{\left({\hat{c}}_{\tau_{1}}-c_{\tau_{1}}^{*},\cdots ,{\hat{c}}_{\tau_{K}}-c_{\tau_{K}}^{*},{\left(\hat{\alpha }-\alpha^{*}\right)}^{⊤},{\left(\hat{\beta }-\beta^{*}\right)}^{⊤}\right)}^{⊤}\overset{\;D\;}{\to }N\left(0,\frac{1}{4}G^{-1}\Sigma^{0}G^{-1}\right),$$

next, we need to compute $\frac{1}{4}G^{-1}\Sigma^{0}G^{-1}$. Denote $G^{-1}=\left(\begin{array}{cc} G^{11} & G^{12}\\ G^{21} & G^{22} \end{array}\right)$, by applying the inverse operation rules of block matrices, we can obtain that

$$\begin{array}{cc} & \;\;\;\;G_{22.1}=G_{22}-G_{21}G_{11}^{-1}G_{12}\\ & =1^{⊤}G_{11}1\Omega -\left\{\left[1^{⊤}G_{11}\right]⊗\mu \right\}G_{11}^{-1}\left\{\left[G_{11}1\right]⊗\mu^{⊤}\right\}\\ & =1^{⊤}G_{11}1\left[\Omega -\mu \mu^{⊤}\right]. \end{array}$$

Because $\Omega_{r}=E\left(r_{t}r_{t}^{⊤}\right)$, $\Pi_{r}=\mathrm{Var}\left(r_{t}\right)$, $\mu_{r}=E\left(r_{t}\right)$, then $\Pi_{r}=\Omega_{r}-\mu_{r}\mu_{r}^{⊤}$; because $\Omega_{z}=E\left(z_{t}z_{t}^{⊤}h_{t}^{-2}\right)$, $\Pi_{z}=\mathrm{Var}\left(\frac{z_{t}}{h_{t}}\right)$, $\mu_{z}=E\left(\frac{z_{t}}{h_{t}}\right)$, then $\Pi_{z}=\Omega_{z}-\mu_{z}\mu_{z}^{⊤}$. Because $\Omega_{rz}=E\left(\frac{z_{t}}{h_{t}}r_{t}^{⊤}\right)$, $\Pi_{rz}=\mathrm{Cov}\left(r_{t},\frac{z_{t}}{h_{t}}\right)$, then $\Pi_{rz}^{⊤}=\mathrm{Cov}\left(\frac{z_{t}}{h_{t}},r_{t}\right)=\Omega_{rz}-\mu_{z}\mu_{r}^{⊤}$; so, we obtain that

$$\Omega -\mu \mu^{⊤}=\Pi .$$

Substituting the above formula into $G_{22.1}$ yields

$$G_{22.1}=1^{⊤}G_{11}1\Pi =\left\{\sum_{k=1}^{K}\omega_{k}\left[\left(1-2\tau_{k}\right)G\left(c_{\tau_{k}}^{*}\right)+\tau_{k}\right]\right\}\Pi .$$

So, we can obtain the expressions for $G^{11}$, $G^{12}$, $G^{21}$ and $G^{22}$ as follows:

(A13) $$\begin{array}{c} \begin{array}{cc} & \;\;\;\;G^{11}=G_{11}^{-1}+G_{11}^{-1}G_{12}G_{22.1}^{-1}G_{21}G_{11}^{-1}\\ & =G_{11}^{-1}+G_{11}^{-1}\left\{\left[G_{11}1\right]⊗\mu^{⊤}\right\}{\left\{1^{⊤}G_{11}1\Pi \right\}}^{-1}\left\{\left[1^{⊤}G_{11}\right]⊗\mu \right\}G_{11}^{-1}\\ & =G_{11}^{-1}+{\left[1^{⊤}G_{11}1\right]}^{-1}\left\{1⊗\mu^{⊤}\right\}\Pi^{-1}\left\{1^{⊤}⊗\mu \right\}\\ & =G_{11}^{-1}+{\left[1^{⊤}G_{11}1\right]}^{-1}\left[\mu^{⊤}\Pi^{-1}\mu \right]11^{⊤}\\ & =\mathrm{diag}\left(\frac{1}{\omega_{1}\left[\left(1-2\tau_{1}\right)G\left(c_{\tau_{1}}^{*}\right)+\tau_{1}\right]},\cdots ,\frac{1}{\omega_{K}\left[\left(1-2\tau_{K}\right)G\left(c_{\tau_{K}}^{*}\right)+\tau_{K}\right]}\right)\\ & \;\;\;\;+\frac{\mu^{⊤}\Pi^{-1}\mu }{\sum_{k=1}^{K}\omega_{k}\left[\left(1-2\tau_{k}\right)G\left(c_{\tau_{k}}^{*}\right)+\tau_{k}\right]}11^{⊤}; \end{array} \end{array}$$

(A14) $$\begin{array}{c} \begin{array}{cc} & \;\;\;\;G^{12}=-G_{11}^{-1}G_{12}G_{22.1}^{-1}\\ & =-G_{11}^{-1}\left\{\left[G_{11}1\right]⊗\mu^{⊤}\right\}{\left\{1^{⊤}G_{11}1\Pi \right\}}^{-1}\\ & =-{\left(1^{⊤}G_{11}1\right)}^{-1}1⊗\left[\mu^{⊤}\Pi^{-1}\right]\\ & =-\frac{1}{\sum_{k=1}^{K}\omega_{k}\left[\left(1-2\tau_{k}\right)G\left(c_{\tau_{k}}^{*}\right)+\tau_{k}\right]}1⊗\left[\mu^{⊤}\Pi^{-1}\right]; \end{array} \end{array}$$

(A15) $$\begin{array}{c} \begin{array}{cc} & \;\;\;\;G^{21}=-G_{22.1}^{-1}G_{21}G_{11}^{-1}\\ & =-{\left(1^{⊤}G_{11}1\right)}^{-1}1^{⊤}⊗\left[\Pi^{-1}\mu \right]\\ & =-\frac{1}{\sum_{k=1}^{K}\omega_{k}\left[\left(1-2\tau_{k}\right)G\left(c_{\tau_{k}}^{*}\right)+\tau_{k}\right]}1^{⊤}⊗\left[\Pi^{-1}\mu \right]; \end{array} \end{array}$$

and

(A16) $$\begin{array}{c} \begin{array}{cc} & \;\;\;\;G^{22}=G_{22.1}^{-1}={\left[1^{⊤}G_{11}1\Pi \right]}^{-1}\\ & =\frac{1}{\sum_{k=1}^{K}\omega_{k}\left[\left(1-2\tau_{k}\right)G\left(c_{\tau_{k}}^{*}\right)+\tau_{k}\right]}\Pi^{-1}. \end{array} \end{array}$$

Denote

$$\Sigma =\left(\begin{array}{cc} \Sigma_{11} & \Sigma_{12}\\ \Sigma_{21} & \Sigma_{22} \end{array}\right)=\frac{1}{4}G^{-1}\Sigma^{0}G^{-1}=\frac{1}{4}\left(\begin{array}{cc} G^{11} & G^{12}\\ G^{21} & G^{22} \end{array}\right)\left(\begin{array}{cc} \Sigma_{11}^{0} & \Sigma_{12}^{0}\\ \Sigma_{21}^{0} & \Sigma_{22}^{0} \end{array}\right)\left(\begin{array}{cc} G^{11} & G^{12}\\ G^{21} & G^{22} \end{array}\right),$$

next, we have to calculate $\Sigma_{11}$, $\Sigma_{12}$, $\Sigma_{21}$ and $\Sigma_{22}$, respectively. First of all,

(A17) $$\begin{array}{c} \Sigma_{11}=\frac{1}{4}\left[G^{11}\Sigma_{11}^{0}G^{11}+G^{12}\Sigma_{21}^{0}G^{11}+G^{11}\Sigma_{12}^{0}G^{21}+G^{12}\Sigma_{22}^{0}G^{21}\right], \end{array}$$

where

(A18) $$\begin{array}{c} \begin{array}{cc} & \;\;\;\;G^{11}\Sigma_{11}^{0}G^{11}\\ & =\left\{G_{11}^{-1}+{\left[1^{⊤}G_{11}1\right]}^{-1}\left[\mu^{⊤}\Pi^{-1}\mu \right]11^{⊤}\right\}\Sigma_{11}^{0}\left\{G_{11}^{-1}+{\left[1^{⊤}G_{11}1\right]}^{-1}\left[\mu^{⊤}\Pi^{-1}\mu \right]11^{⊤}\right\}\\ & =G_{11}^{-1}\Sigma_{11}^{0}G_{11}^{-1}+G_{11}^{-1}\Sigma_{11}^{0}{\left[1^{⊤}G_{11}1\right]}^{-1}\left[\mu^{⊤}\Pi^{-1}\mu \right]11^{⊤}\\ & \;\;\;\;+{\left[1^{⊤}G_{11}1\right]}^{-1}\left[\mu^{⊤}\Pi^{-1}\mu \right]11^{⊤}\Sigma_{11}^{0}G_{11}^{-1}\\ & \;\;\;\;+{\left[1^{⊤}G_{11}1\right]}^{-1}\left[\mu^{⊤}\Pi^{-1}\mu \right]11^{⊤}\Sigma_{11}^{0}{\left[1^{⊤}G_{11}1\right]}^{-1}\left[\mu^{⊤}\Pi^{-1}\mu \right]11^{⊤}\\ & =G_{11}^{-1}\Sigma_{11}^{0}G_{11}^{-1}+\frac{\mu^{⊤}\Pi^{-1}\mu }{1^{⊤}G_{11}1}\left[G_{11}^{-1}\Sigma_{11}^{0}11^{⊤}+11^{⊤}\Sigma_{11}^{0}G_{11}^{-1}\right]+{\left[\frac{\mu^{⊤}\Pi^{-1}\mu }{1^{⊤}G_{11}1}\right]}^{2}\left(1^{⊤}\Sigma_{11}^{0}1\right)11^{⊤}; \end{array} \end{array}$$

(A19) $$\begin{array}{c} \begin{array}{cc} & \;\;\;\;G^{12}\Sigma_{21}^{0}G^{11}\\ & =\left\{-{\left(1^{⊤}G_{11}1\right)}^{-1}1⊗\left[\mu^{⊤}\Pi^{-1}\right]\right\}\left\{\left[1^{⊤}\Sigma_{11}^{0}\right]⊗\mu \right\}\\ & \;\;\;\;\left\{G_{11}^{-1}+{\left[1^{⊤}G_{11}1\right]}^{-1}\left[\mu^{⊤}\Pi^{-1}\mu \right]11^{⊤}\right\}\\ & =-\frac{\mu^{⊤}\Pi^{-1}\mu }{1^{⊤}G_{11}1}11^{⊤}\Sigma_{11}^{0}G_{11}^{-1}-{\left[\frac{\mu^{⊤}\Pi^{-1}\mu }{1^{⊤}G_{11}1}\right]}^{2}\left(1^{⊤}\Sigma_{11}^{0}1\right)11^{⊤}; \end{array} \end{array}$$

(A20) $$\begin{array}{c} \begin{array}{cc} & \;\;\;\;G^{11}\Sigma_{12}^{0}G^{21}={\left[G^{12}\Sigma_{21}^{0}G^{11}\right]}^{⊤}\\ & =-\frac{\mu^{⊤}\Pi^{-1}\mu }{1^{⊤}G_{11}1}G_{11}^{-1}\Sigma_{11}^{0}11^{⊤}-{\left[\frac{\mu^{⊤}\Pi^{-1}\mu }{1^{⊤}G_{11}1}\right]}^{2}\left(1^{⊤}\Sigma_{11}^{0}1\right)11^{⊤}; \end{array} \end{array}$$

(A21) $$\begin{array}{c} \begin{array}{cc} & \;\;\;\;G^{12}\Sigma_{22}^{0}G^{21}\\ & =\left\{-{\left(1^{⊤}G_{11}1\right)}^{-1}1⊗\left[\mu^{⊤}\Pi^{-1}\right]\right\}\left\{1^{⊤}\Sigma_{11}^{0}1\Omega \right\}\left\{-{\left(1^{⊤}G_{11}1\right)}^{-1}1^{⊤}⊗\left[\Pi^{-1}\mu \right]\right\}\\ & ={\left(1^{⊤}G_{11}1\right)}^{-2}\left(1^{⊤}\Sigma_{11}^{0}1\right)\left(\mu^{⊤}\Pi^{-1}\Omega \Pi^{-1}\mu \right)11^{⊤}\\ & ={\left(1^{⊤}G_{11}1\right)}^{-2}\left(1^{⊤}\Sigma_{11}^{0}1\right)\left[\mu^{⊤}\Pi^{-1}\left(\Pi +\mu \mu^{⊤}\right)\Pi^{-1}\mu \right]11^{⊤}\\ & ={\left(1^{⊤}G_{11}1\right)}^{-2}\left(1^{⊤}\Sigma_{11}^{0}1\right)\left[\mu^{⊤}\Pi^{-1}\mu_{z}+{\left(\mu^{⊤}\Pi^{-1}\mu \right)}^{2}\right]11^{⊤}. \end{array} \end{array}$$

So, by substituting the results of (A18)–(A21) into the expression for $\Sigma_{11}$ in (A17), we can obtain the value of $\Sigma_{11}$ as

(A22) $$\begin{array}{c} \begin{array}{cc} & \;\;\;\;\Sigma_{11}=\frac{1}{4}\left[G^{11}\Sigma_{11}^{0}G^{11}+G^{12}\Sigma_{21}^{0}G^{11}+G^{11}\Sigma_{12}^{0}G^{21}+G^{12}\Sigma_{22}^{0}G^{21}\right]\\ & =\frac{1}{4}G_{11}^{-1}\Sigma_{11}^{0}G_{11}^{-1}+\frac{1}{4}{\left(1^{⊤}G_{11}1\right)}^{-2}\left(1^{⊤}\Sigma_{11}^{0}1\right)\left(\mu^{⊤}\Pi^{-1}\mu \right)11^{⊤}\\ & =\xi +\sigma^{2}\left(\omega \right)\left(\mu^{⊤}\Pi^{-1}\mu \right)11^{⊤}, \end{array} \end{array}$$

where ξ is a K×K matrix with the (j,k)th element being

$$\xi_{jk}=\frac{E\left[{\dot{Q}}_{\tau_{j}}\left(e_{t}-c_{\tau_{j}}^{*}\right){\dot{Q}}_{\tau_{k}}\left(e_{t}-c_{\tau_{k}}^{*}\right)\right]}{4\left[\left(1-2\tau_{j}\right)G\left(c_{\tau_{j}}^{*}\right)+\tau_{j}\right]\left[\left(1-2\tau_{k}\right)G\left(c_{\tau_{k}}^{*}\right)+\tau_{k}\right]},$$

$$\sigma^{2}\left(\omega \right)=\frac{\omega^{⊤}\Lambda \omega }{{\left[\sum_{k=1}^{K}\omega_{k}\left(\left(1-2\tau_{k}\right)G\left(c_{\tau_{k}}^{*}\right)+\tau_{k}\right)\right]}^{2}},$$

with Λ is a K×K matrix with the (j,k)th element being Λ(j,k),

$$\begin{array}{cc} & \;\;\;\;\Lambda \left(j,k\right)=\frac{1}{4}E\left\{{\dot{Q}}_{\tau_{j}}\left(e_{t}-c_{\tau_{j}}^{*}\right){\dot{Q}}_{\tau_{k}}\left(e_{t}-c_{\tau_{k}}^{*}\right)\right\}\\ & =\int \left|\tau_{j}\tau_{k}-\tau_{j}I\left(e_{t}\leq c_{\tau_{k}}^{*}\right)-\tau_{k}I\left(e_{t}\leq c_{\tau_{j}}^{*}\right)+I\left(e_{t}\leq c_{\tau_{j}}^{*}\wedge c_{\tau_{k}}^{*}\right)\right|\left[e_{t}^{2}-\left(c_{\tau_{j}}^{*}+c_{\tau_{k}}^{*}\right)e_{t}+c_{\tau_{j}}^{*}c_{\tau_{k}}^{*}\right]dG\left(e_{t}\right). \end{array}$$

Secondly, we have

(A23) $$\begin{array}{c} \Sigma_{12}=\frac{1}{4}\left[G^{11}\Sigma_{11}^{0}G^{12}+G^{12}\Sigma_{21}^{0}G^{12}+G^{11}\Sigma_{12}^{0}G^{22}+G^{12}\Sigma_{22}^{0}G^{22}\right], \end{array}$$

where

(A24) $$\begin{array}{c} \begin{array}{cc} & \;\;\;\;G^{11}\Sigma_{11}^{0}G^{12}\\ & =\left\{G_{11}^{-1}+{\left[1^{⊤}G_{11}1\right]}^{-1}\left[\mu^{⊤}\Pi^{-1}\mu \right]11^{⊤}\right\}\Sigma_{11}^{0}\left\{-{\left(1^{⊤}G_{11}1\right)}^{-1}1⊗\left[\mu^{⊤}\Pi^{-1}\right]\right\}\\ & =-{\left(1^{⊤}G_{11}1\right)}^{-1}\left(G_{11}^{-1}\Sigma_{11}^{0}1\right)⊗\left(\mu^{⊤}\Pi^{-1}\right)\\ & \;\;\;\;-{\left(1^{⊤}G_{11}1\right)}^{-2}\left(\mu^{⊤}\Pi^{-1}\mu \right)\left(1^{⊤}\Sigma_{11}^{0}1\right)1⊗\left(\mu^{⊤}\Pi^{-1}\right); \end{array} \end{array}$$

(A25) $$\begin{array}{c} \begin{array}{cc} & \;\;\;\;G^{12}\Sigma_{21}^{0}G^{12}\\ & =\left\{-{\left(1^{⊤}G_{11}1\right)}^{-1}1⊗\left[\mu^{⊤}\Pi^{-1}\right]\right\}\left\{\left[1^{⊤}\Sigma_{11}^{0}\right]⊗\mu \right\}\left\{-{\left(1^{⊤}G_{11}1\right)}^{-1}1⊗\left[\mu^{⊤}\Pi^{-1}\right]\right\}\\ & ={\left(1^{⊤}G_{11}1\right)}^{-2}\left(1^{⊤}\Sigma_{11}^{0}1\right)\left(\mu^{⊤}\Pi^{-1}\mu \right)1⊗\left(\mu^{⊤}\Pi^{-1}\right); \end{array} \end{array}$$

(A26) $$\begin{array}{c} \begin{array}{cc} & \;\;\;\;G^{11}\Sigma_{12}^{0}G^{22}\\ & =\left\{G_{11}^{-1}+{\left[1^{⊤}G_{11}1\right]}^{-1}\left[\mu^{⊤}\Pi^{-1}\mu \right]11^{⊤}\right\}\left\{\left[\Sigma_{11}^{0}1\right]⊗\mu^{⊤}\right\}\left\{{\left[1^{⊤}G_{11}1\Pi_{z}\right]}^{-1}\right\}\\ & ={\left(1^{⊤}G_{11}1\right)}^{-1}\left(G_{11}^{-1}\Sigma_{11}^{0}1\right)⊗\left(\mu^{⊤}\Pi^{-1}\right)\\ & \;\;\;\;+{\left(1^{⊤}G_{11}1\right)}^{-2}\left(\mu^{⊤}\Pi^{-1}\mu \right)\left(1^{⊤}\Sigma_{11}^{0}1\right)1⊗\left(\mu^{⊤}\Pi^{-1}\right); \end{array} \end{array}$$

(A27) $$\begin{array}{c} \begin{array}{cc} G^{12}\Sigma_{22}^{0}G^{22} & =\left\{-{\left(1^{⊤}G_{11}1\right)}^{-1}1⊗\left[\mu^{⊤}\Pi^{-1}\right]\right\}\left\{1^{⊤}\Sigma_{11}^{0}1\Omega \right\}\left\{{\left[1^{⊤}G_{11}1\Pi \right]}^{-1}\right\}\\ & =-{\left(1^{⊤}G_{11}1\right)}^{-2}\left(1^{⊤}\Sigma_{11}^{0}1\right)1⊗\left(\mu^{⊤}\Pi^{-1}\Omega \Pi^{-1}\right)\\ & =-{\left(1^{⊤}G_{11}1\right)}^{-2}\left(1^{⊤}\Sigma_{11}^{0}1\right)1⊗\left[\mu^{⊤}\Pi^{-1}\left(\Pi +\mu \mu^{⊤}\right)\Pi^{-1}\right]\\ & =-{\left(1^{⊤}G_{11}1\right)}^{-2}\left(1^{⊤}\Sigma_{11}^{0}1\right)1⊗\left(\mu^{⊤}\Pi^{-1}\right)\\ & \;\;\;\;-{\left(1^{⊤}G_{11}1\right)}^{-2}\left(1^{⊤}\Sigma_{11}^{0}1\right)\left(\mu^{⊤}\Pi^{-1}\mu \right)1⊗\left(\mu^{⊤}\Pi^{-1}\right). \end{array} \end{array}$$

So, by substituting the results of (A24)–(A27) into the expression for $\Sigma_{12}$ in (A23), we can obtain the value of $\Sigma_{12}$ as

(A28) $$\begin{array}{c} \begin{array}{cc} & \;\;\;\;\Sigma_{12}=\frac{1}{4}\left[G^{11}\Sigma_{11}^{0}G^{12}+G^{12}\Sigma_{21}^{0}G^{12}+G^{11}\Sigma_{12}^{0}G^{22}+G^{12}\Sigma_{22}^{0}G^{22}\right]\\ & =-\frac{1}{4}{\left(1^{⊤}G_{11}1\right)}^{-2}\left(1^{⊤}\Sigma_{11}^{0}1\right)1⊗\left(\mu^{⊤}\Pi^{-1}\right)\\ & =-\sigma^{2}\left(\omega \right)1⊗\left(\mu^{⊤}\Pi^{-1}\right). \end{array} \end{array}$$

Thirdly, we have

(A29) $$\begin{array}{c} \Sigma_{21}={\left(\Sigma_{12}\right)}^{⊤}=-\sigma^{2}\left(\omega \right)1^{⊤}⊗\left(\Pi^{-1}\mu \right). \end{array}$$

Fourthly, we have

(A30) $$\begin{array}{c} \Sigma_{22}=\frac{1}{4}\left[G^{21}\Sigma_{11}^{0}G^{12}+G^{22}\Sigma_{21}^{0}G^{12}+G^{21}\Sigma_{12}^{0}G^{22}+G^{22}\Sigma_{22}^{0}G^{22}\right], \end{array}$$

where

(A31) $$\begin{array}{c} \begin{array}{cc} G^{21}\Sigma_{11}^{0}G^{12} & =\left\{-{\left(1^{⊤}G_{11}1\right)}^{-1}1^{⊤}⊗\left[\Pi^{-1}\mu \right]\right\}\Sigma_{11}^{0}\left\{-{\left(1^{⊤}G_{11}1\right)}^{-1}1⊗\left[\mu^{⊤}\Pi^{-1}\right]\right\}\\ & ={\left(1^{⊤}G_{11}1\right)}^{-2}\left(1^{⊤}\Sigma_{11}^{0}1\right)\left(\Pi^{-1}\mu \mu^{⊤}\Pi^{-1}\right); \end{array} \end{array}$$

(A32) $$\begin{array}{c} \begin{array}{cc} G^{22}\Sigma_{21}^{0}G^{12} & =\left\{{\left[1^{⊤}G_{11}1\Pi \right]}^{-1}\right\}\left\{\left[1^{⊤}\Sigma_{11}^{0}\right]⊗\mu \right\}\left\{-{\left(1^{⊤}G_{11}1\right)}^{-1}1⊗\left[\mu^{⊤}\Pi^{-1}\right]\right\}\\ & =-{\left(1^{⊤}G_{11}1\right)}^{-2}\left(1^{⊤}\Sigma_{11}^{0}1\right)\left(\Pi^{-1}\mu \mu^{⊤}\Pi^{-1}\right); \end{array} \end{array}$$

(A33) $$\begin{array}{c} G^{21}\Sigma_{12}^{0}G^{22}={\left(G^{22}\Sigma_{21}^{0}G^{12}\right)}^{⊤}=-{\left(1^{⊤}G_{11}1\right)}^{-2}\left(1^{⊤}\Sigma_{11}^{0}1\right)\left(\Pi^{-1}\mu \mu^{⊤}\Pi^{-1}\right); \end{array}$$

(A34) $$\begin{array}{c} \begin{array}{cc} G^{22}\Sigma_{22}^{0}G^{22} & =\left\{{\left[1^{⊤}G_{11}1\Pi \right]}^{-1}\right\}\left\{1^{⊤}\Sigma_{11}^{0}1\Omega \right\}\left\{{\left[1^{⊤}G_{11}1\Pi \right]}^{-1}\right\}\\ & ={\left(1^{⊤}G_{11}1\right)}^{-2}\left(1^{⊤}\Sigma_{11}^{0}1\right)\left(\Pi^{-1}\Omega \Pi^{-1}\right)\\ & ={\left(1^{⊤}G_{11}1\right)}^{-2}\left(1^{⊤}\Sigma_{11}^{0}1\right)\left[\Pi^{-1}\left(\Pi +\mu \mu^{⊤}\right)\Pi^{-1}\right]\\ & ={\left(1^{⊤}G_{11}1\right)}^{-2}\left(1^{⊤}\Sigma_{11}^{0}1\right)\left[\Pi^{-1}+\Pi^{-1}\mu \mu^{⊤}\Pi^{-1}\right]. \end{array} \end{array}$$

So, by substituting the results of (A31)–(A34) into the expression for $\Sigma_{22}$ in (A30), we can obtain the value of $\Sigma_{22}$ as

(A35) $$\begin{array}{c} \begin{array}{cc} & \;\;\;\;\Sigma_{22}=\frac{1}{4}\left[G^{21}\Sigma_{11}^{0}G^{12}+G^{22}\Sigma_{21}^{0}G^{12}+G^{21}\Sigma_{12}^{0}G^{22}+G^{22}\Sigma_{22}^{0}G^{22}\right]\\ & =\frac{1}{4}{\left(1^{⊤}G_{11}1\right)}^{-2}\left(1^{⊤}\Sigma_{11}^{0}1\right)\Pi^{-1}\\ & =\sigma^{2}\left(\omega \right)\Pi^{-1}. \end{array} \end{array}$$

Therefore, we obtain that

$$\sqrt{n}{\left({\hat{c}}_{\tau_{1}}-c_{\tau_{1}}^{*},\cdots ,{\hat{c}}_{\tau_{K}}-c_{\tau_{K}}^{*},{\left(\hat{\alpha }-\alpha^{*}\right)}^{⊤},{\left(\hat{\beta }-\beta^{*}\right)}^{⊤}\right)}^{⊤}\overset{\;D\;}{\to }N\left(0,\Sigma \right),$$

where $\Sigma =\left(\begin{array}{cc} \Sigma_{11} & \Sigma_{12}\\ \Sigma_{21} & \Sigma_{22} \end{array}\right)$. We have now concluded the proof of Theorem 4. □

## Appendix B. Partial Results of Simulation Studies

**Table A1:** Parameter estimate of various estimation methods, $\epsilon_{t}∼N\left(0,1\right)$.

	n=100									
Estimate	$\alpha_{1}^{\left(1\right)}$	$\alpha_{2}^{\left(1\right)}$	$\alpha_{1}^{\left(2\right)}$	$\alpha_{2}^{\left(2\right)}$	$\beta_{0}^{\left(1\right)}$	$\beta_{1}^{\left(1\right)}$	$\beta_{2}^{\left(1\right)}$	$\beta_{0}^{\left(2\right)}$	$\beta_{1}^{\left(2\right)}$	$\beta_{2}^{\left(2\right)}$
MLE	0.2357	0.2902	0.4264	0.1868	0.0349	0.2864	0.4907	0.0638	0.4346	0.3369
LS	0.2342	0.2878	0.4228	0.1854	0.0354	0.2852	0.4881	0.0641	0.4333	0.3357
QR${}_{0.25}$	0.2141	0.1569	0.4113	0.1019	0.0441	0.2249	0.4344	0.0789	0.3899	0.2959
QR${}_{0.75}$	0.2148	0.1752	0.4134	0.1089	0.0439	0.2256	0.4356	0.0792	0.3901	0.2981
ER${}_{0.25}$	0.2099	0.2123	0.4134	0.1099	0.0430	0.2257	0.4312	0.0794	0.3914	0.2953
ER${}_{0.75}$	0.2084	0.2101	0.4129	0.1098	0.0441	0.2241	0.4346	0.0791	0.3913	0.2953
WCER	0.2159	0.2257	0.4265	0.1439	0.0354	0.2459	0.4753	0.0671	0.4056	0.3149
	n=300									
**Estimate**	$\alpha_{1}^{\left(1\right)}$	$\alpha_{2}^{\left(1\right)}$	$\alpha_{1}^{\left(2\right)}$	$\alpha_{2}^{\left(2\right)}$	$\beta_{0}^{\left(1\right)}$	$\beta_{1}^{\left(1\right)}$	$\beta_{2}^{\left(1\right)}$	$\beta_{0}^{\left(2\right)}$	$\beta_{1}^{\left(2\right)}$	$\beta_{2}^{\left(2\right)}$
MLE	0.2417	0.2913	0.4396	0.1907	0.0327	0.2903	0.4936	0.0623	0.4403	0.3421
LS	0.2404	0.2902	0.4390	0.1902	0.0331	0.2902	0.4925	0.0631	0.4391	0.3404
QR${}_{0.25}$	0.2209	0.2387	0.4242	0.1499	0.0401	0.2512	0.4548	0.0717	0.4024	0.3080
QR${}_{0.75}$	0.2213	0.2389	0.4244	0.1508	0.0398	0.2508	0.4539	0.0729	0.4011	0.3079
ER${}_{0.25}$	0.2203	0.2399	0.4233	0.1508	0.0399	0.2489	0.4553	0.0727	0.4013	0.3086
ER${}_{0.75}$	0.2202	0.2380	0.4239	0.1511	0.0392	0.2513	0.4546	0.0741	0.4006	0.3089
WCER	0.2295	0.2604	0.4376	0.1701	0.0339	0.2824	0.4869	0.0642	0.4248	0.3302
	n=800									
**Estimate**	$\alpha_{1}^{\left(1\right)}$	$\alpha_{2}^{\left(1\right)}$	$\alpha_{1}^{\left(2\right)}$	$\alpha_{2}^{\left(2\right)}$	$\beta_{0}^{\left(1\right)}$	$\beta_{1}^{\left(1\right)}$	$\beta_{2}^{\left(1\right)}$	$\beta_{0}^{\left(2\right)}$	$\beta_{1}^{\left(2\right)}$	$\beta_{2}^{\left(2\right)}$
MLE	0.2449	0.2928	0.4446	0.1925	0.0316	0.2944	0.4958	0.0617	0.4434	0.3449
LS	0.2441	0.2919	0.4443	0.1919	0.0318	0.2941	0.4951	0.0620	0.4430	0.3441
QR${}_{0.25}$	0.2311	0.2578	0.4313	0.1641	0.0372	0.2588	0.4661	0.0687	0.4099	0.3191
QR${}_{0.75}$	0.2306	0.2584	0.4321	0.1644	0.0371	0.2580	0.4656	0.0689	0.4101	0.3195
ER${}_{0.25}$	0.2301	0.2579	0.4311	0.1658	0.0376	0.2594	0.4659	0.0692	0.4108	0.3188
ER${}_{0.75}$	0.2294	0.2581	0.4314	0.1654	0.0374	0.2587	0.4648	0.0696	0.4106	0.3191
WCER	0.2409	0.2802	0.4418	0.1814	0.0322	0.2901	0.4918	0.0624	0.4337	0.3379
	n=1500									
**Estimate**	$\alpha_{1}^{\left(1\right)}$	$\alpha_{2}^{\left(1\right)}$	$\alpha_{1}^{\left(2\right)}$	$\alpha_{2}^{\left(2\right)}$	$\beta_{0}^{\left(1\right)}$	$\beta_{1}^{\left(1\right)}$	$\beta_{2}^{\left(1\right)}$	$\beta_{0}^{\left(2\right)}$	$\beta_{1}^{\left(2\right)}$	$\beta_{2}^{\left(2\right)}$
MLE	0.2467	0.2955	0.4462	0.1954	0.0306	0.2977	0.4971	0.0605	0.4465	0.3473
LS	0.2462	0.2944	0.4460	0.1951	0.0308	0.2972	0.4968	0.0606	0.4460	0.3469
QR${}_{0.25}$	0.2361	0.2732	0.4435	0.1862	0.0336	0.2704	0.4761	0.0651	0.4241	0.3269
QR${}_{0.75}$	0.2368	0.2738	0.4437	0.1858	0.0339	0.2711	0.4758	0.0651	0.4239	0.3263
ER${}_{0.25}$	0.2355	0.2721	0.4435	0.1864	0.0338	0.2701	0.4759	0.0649	0.4231	0.3277
ER${}_{0.75}$	0.2364	0.2718	0.4432	0.1871	0.0341	0.2705	0.4755	0.0652	0.4229	0.3266
WCER	0.2446	0.2903	0.4459	0.1945	0.0307	0.2924	0.4964	0.0607	0.4451	0.3467
	n=2500									
**Estimate**	$\alpha_{1}^{\left(1\right)}$	$\alpha_{2}^{\left(1\right)}$	$\alpha_{1}^{\left(2\right)}$	$\alpha_{2}^{\left(2\right)}$	$\beta_{0}^{\left(1\right)}$	$\beta_{1}^{\left(1\right)}$	$\beta_{2}^{\left(1\right)}$	$\beta_{0}^{\left(2\right)}$	$\beta_{1}^{\left(2\right)}$	$\beta_{2}^{\left(2\right)}$
MLE	0.2485	0.2978	0.4483	0.1976	0.0302	0.2988	0.4985	0.0602	0.4483	0.3487
LS	0.2483	0.2972	0.4479	0.1970	0.0304	0.2983	0.4980	0.06004	0.4478	0.3484
QR${}_{0.25}$	0.2424	0.2858	0.4463	0.1924	0.0316	0.2858	0.4877	0.0625	0.4362	0.3373
QR${}_{0.75}$	0.2439	0.2856	0.4465	0.1926	0.0319	0.2851	0.4875	0.0626	0.4364	0.3371
ER${}_{0.25}$	0.2428	0.2853	0.4465	0.1925	0.0318	0.2853	0.4872	0.0624	0.4361	0.3378
ER${}_{0.75}$	0.2437	0.2850	0.4462	0.1922	0.0320	0.2850	0.4874	0.0625	0.4363	0.3376
WCER	0.2474	0.2960	0.4477	0.1965	0.0305	0.2980	0.4979	0.0605	0.4475	0.3482

**Table A2:** Parameter estimate of various estimation methods, $\epsilon_{t}∼t\left(6\right)$.

	n=100									
Estimate	$\alpha_{1}^{\left(1\right)}$	$\alpha_{2}^{\left(1\right)}$	$\alpha_{1}^{\left(2\right)}$	$\alpha_{2}^{\left(2\right)}$	$\beta_{0}^{\left(1\right)}$	$\beta_{1}^{\left(1\right)}$	$\beta_{2}^{\left(1\right)}$	$\beta_{0}^{\left(2\right)}$	$\beta_{1}^{\left(2\right)}$	$\beta_{2}^{\left(2\right)}$
MLE	0.2362	0.2762	0.4302	0.1822	0.0346	0.2869	0.4903	0.0638	0.4337	0.3394
LS	0.2135	0.2057	0.4181	0.2707	0.0409	0.2341	0.4424	0.0765	0.4004	0.3038
QR${}_{0.25}$	0.2119	0.2044	0.4189	0.2756	0.0412	0.3689	0.4418	0.0759	0.3988	0.3030
QR${}_{0.75}$	0.2121	0.2055	0.4180	0.1248	0.0410	0.3692	0.4411	0.0761	0.3980	0.3025
ER${}_{0.25}$	0.2122	0.2039	0.4187	0.1250	0.0416	0.2299	0.4420	0.0767	0.3996	0.3036
ER${}_{0.75}$	0.2118	0.2049	0.4176	0.1255	0.0422	0.2296	0.4416	0.0762	0.3988	0.3039
WCER	0.2179	0.2095	0.4272	0.1436	0.0365	0.2501	0.4851	0.0678	0.4106	0.3177
	n=300									
**Estimate**	$\alpha_{1}^{\left(1\right)}$	$\alpha_{2}^{\left(1\right)}$	$\alpha_{1}^{\left(2\right)}$	$\alpha_{2}^{\left(2\right)}$	$\beta_{0}^{\left(1\right)}$	$\beta_{1}^{\left(1\right)}$	$\beta_{2}^{\left(1\right)}$	$\beta_{0}^{\left(2\right)}$	$\beta_{1}^{\left(2\right)}$	$\beta_{2}^{\left(2\right)}$
MLE	0.2424	0.2894	0.4436	0.1913	0.0329	0.2918	0.4948	0.0621	0.4406	0.3426
LS	0.2228	0.2446	0.4287	0.2421	0.0376	0.2563	0.4645	0.0705	0.4084	0.3156
QR${}_{0.25}$	0.2221	0.2433	0.4259	0.2478	0.0379	0.3451	0.4633	0.0709	0.4045	0.3151
QR${}_{0.75}$	0.2218	0.2430	0.4256	0.1524	0.0381	0.3453	0.4639	0.0707	0.4043	0.3159
ER${}_{0.25}$	0.2225	0.2424	0.4264	0.1539	0.0381	0.2543	0.4624	0.0712	0.4044	0.3142
ER${}_{0.75}$	0.2223	0.2428	0.4255	0.1534	0.0387	0.2551	0.4626	0.0716	0.4039	0.3151
WCER	0.2321	0.2646	0.4384	0.1708	0.0337	0.2821	0.4899	0.0641	0.4259	0.3313
	n=800									
**Estimate**	$\alpha_{1}^{\left(1\right)}$	$\alpha_{2}^{\left(1\right)}$	$\alpha_{1}^{\left(2\right)}$	$\alpha_{2}^{\left(2\right)}$	$\beta_{0}^{\left(1\right)}$	$\beta_{1}^{\left(1\right)}$	$\beta_{2}^{\left(1\right)}$	$\beta_{0}^{\left(2\right)}$	$\beta_{1}^{\left(2\right)}$	$\beta_{2}^{\left(2\right)}$
MLE	0.2454	0.2922	0.4458	0.1936	0.0309	0.2947	0.4956	0.0611	0.4444	0.3458
LS	0.2319	0.2639	0.4325	0.2268	0.0343	0.2699	0.4720	0.0656	0.4203	0.3262
QR${}_{0.25}$	0.2307	0.2625	0.4323	0.2288	0.0370	0.3311	0.4702	0.0669	0.4189	0.3260
QR${}_{0.75}$	0.2305	0.2614	0.4324	0.1721	0.0369	0.3320	0.4705	0.0671	0.4184	0.3256
ER${}_{0.25}$	0.2309	0.2628	0.4331	0.1709	0.0366	0.2683	0.4699	0.0678	0.4194	0.3261
ER${}_{0.75}$	0.2300	0.2619	0.4329	0.1695	0.0368	0.2669	0.4687	0.0681	0.4183	0.3249
WCER	0.2412	0.2801	0.4423	0.1824	0.0314	0.2908	0.4913	0.0618	0.4346	0.3395
	n=1500									
**Estimate**	$\alpha_{1}^{\left(1\right)}$	$\alpha_{2}^{\left(1\right)}$	$\alpha_{1}^{\left(2\right)}$	$\alpha_{2}^{\left(2\right)}$	$\beta_{0}^{\left(1\right)}$	$\beta_{1}^{\left(1\right)}$	$\beta_{2}^{\left(1\right)}$	$\beta_{0}^{\left(2\right)}$	$\beta_{1}^{\left(2\right)}$	$\beta_{2}^{\left(2\right)}$
MLE	0.2477	0.2949	0.4473	0.1955	0.0304	0.2969	0.4972	0.0607	0.4459	0.3466
LS	0.2397	0.2804	0.4398	0.2168	0.0323	0.2808	0.4813	0.0647	0.4309	0.3353
QR${}_{0.25}$	0.2389	0.2799	0.4384	0.2176	0.0335	0.3203	0.4811	0.0653	0.4301	0.3349
QR${}_{0.75}$	0.2383	0.2787	0.4379	0.1821	0.0337	0.3209	0.4808	0.0655	0.4297	0.3341
ER${}_{0.25}$	0.2385	0.2792	0.4388	0.1823	0.0333	0.2792	0.4803	0.0651	0.4295	0.3348
ER${}_{0.75}$	0.2379	0.2783	0.4382	0.1832	0.0338	0.2790	0.4809	0.0649	0.4284	0.3340
WCER	0.2449	0.2898	0.4469	0.1933	0.0308	0.2932	0.4954	0.0610	0.4407	0.3419
	n=2500									
**Estimate**	$\alpha_{1}^{\left(1\right)}$	$\alpha_{2}^{\left(1\right)}$	$\alpha_{1}^{\left(2\right)}$	$\alpha_{2}^{\left(2\right)}$	$\beta_{0}^{\left(1\right)}$	$\beta_{1}^{\left(1\right)}$	$\beta_{2}^{\left(1\right)}$	$\beta_{0}^{\left(2\right)}$	$\beta_{1}^{\left(2\right)}$	$\beta_{2}^{\left(2\right)}$
MLE	0.2488	0.2977	0.4485	0.1976	0.0302	0.2983	0.4986	0.0604	0.4479	0.3482
LS	0.2438	0.2891	0.4441	0.2069	0.0315	0.2887	0.4898	0.0625	0.4401	0.3413
QR${}_{0.25}$	0.2440	0.2888	0.4439	0.2073	0.0317	0.3117	0.4891	0.0627	0.4398	0.3408
QR${}_{0.75}$	0.2437	0.2887	0.4436	0.1921	0.0319	0.3115	0.4889	0.0629	0.4396	0.3410
ER${}_{0.25}$	0.2436	0.2889	0.4437	0.1922	0.0316	0.2881	0.4893	0.0633	0.4395	0.3411
ER${}_{0.75}$	0.2437	0.2883	0.4434	0.1928	0.0321	0.2877	0.4887	0.0631	0.4397	0.3409
WCER	0.2475	0.2941	0.4481	0.1966	0.0304	0.2964	0.4976	0.0607	0.4452	0.3468

**Table A3:** Parameter estimate of various estimation methods, $\epsilon_{t}∼\chi^{2}\left(4\right)$.

	n=100									
Estimate	$\alpha_{1}^{\left(1\right)}$	$\alpha_{2}^{\left(1\right)}$	$\alpha_{1}^{\left(2\right)}$	$\alpha_{2}^{\left(2\right)}$	$\beta_{0}^{\left(1\right)}$	$\beta_{1}^{\left(1\right)}$	$\beta_{2}^{\left(1\right)}$	$\beta_{0}^{\left(2\right)}$	$\beta_{1}^{\left(2\right)}$	$\beta_{2}^{\left(2\right)}$
MLE	0.2377	0.2772	0.4352	0.1869	0.0337	0.2883	0.4892	0.0651	0.4377	0.3397
LS	0.2126	0.2009	0.4018	0.2833	0.0411	0.2244	0.5609	0.0767	0.3953	0.3002
QR${}_{0.25}$	0.2151	0.2048	0.4927	0.2839	0.0417	0.2239	0.5601	0.0769	0.3962	0.4001
QR${}_{0.75}$	0.2148	0.2037	0.4944	0.1149	0.0421	0.2222	0.4396	0.0762	0.3956	0.4007
ER${}_{0.25}$	0.2161	0.2057	0.4084	0.1179	0.0409	0.2255	0.4406	0.0757	0.3974	0.3006
ER${}_{0.75}$	0.2153	0.2061	0.4069	0.1128	0.0417	0.2218	0.4401	0.0772	0.3947	0.2995
WCER	0.2239	0.2123	0.4289	0.1518	0.0365	0.2519	0.4757	0.0683	0.4181	0.3188
	n=300									
**Estimate**	$\alpha_{1}^{\left(1\right)}$	$\alpha_{2}^{\left(1\right)}$	$\alpha_{1}^{\left(2\right)}$	$\alpha_{2}^{\left(2\right)}$	$\beta_{0}^{\left(1\right)}$	$\beta_{1}^{\left(1\right)}$	$\beta_{2}^{\left(1\right)}$	$\beta_{0}^{\left(2\right)}$	$\beta_{1}^{\left(2\right)}$	$\beta_{2}^{\left(2\right)}$
MLE	0.2414	0.2891	0.4439	0.1922	0.0318	0.2922	0.4951	0.0621	0.4419	0.3437
LS	0.2235	0.2456	0.4268	0.2344	0.0381	0.2655	0.5304	0.0701	0.4160	0.3227
QR${}_{0.25}$	0.2242	0.2469	0.4722	0.2342	0.0379	0.2667	0.5307	0.0699	0.4169	0.3771
QR${}_{0.75}$	0.2240	0.2459	0.4731	0.1651	0.0381	0.2661	0.4694	0.0698	0.4163	0.3773
ER${}_{0.25}$	0.2248	0.2477	0.4281	0.1664	0.0373	0.2676	0.4699	0.0698	0.4174	0.3240
ER${}_{0.75}$	0.2222	0.2445	0.4263	0.1649	0.0385	0.2642	0.4691	0.0712	0.4147	0.3224
WCER	0.2328	0.2667	0.4380	0.1728	0.0335	0.2839	0.4892	0.0638	0.4294	0.3329
	n=800									
**Estimate**	$\alpha_{1}^{\left(1\right)}$	$\alpha_{2}^{\left(1\right)}$	$\alpha_{1}^{\left(2\right)}$	$\alpha_{2}^{\left(2\right)}$	$\beta_{0}^{\left(1\right)}$	$\beta_{1}^{\left(1\right)}$	$\beta_{2}^{\left(1\right)}$	$\beta_{0}^{\left(2\right)}$	$\beta_{1}^{\left(2\right)}$	$\beta_{2}^{\left(2\right)}$
MLE	0.2456	0.2944	0.4463	0.1949	0.0310	0.2956	0.4965	0.0612	0.4446	0.3459
LS	0.2314	0.2613	0.4336	0.2289	0.0363	0.2705	0.5227	0.0684	0.4225	0.3286
QR${}_{0.25}$	0.2321	0.2629	0.4661	0.2281	0.0361	0.2709	0.5220	0.0681	0.4227	0.3717
QR${}_{0.75}$	0.2317	0.2623	0.4667	0.1713	0.0365	0.2703	0.4775	0.0686	0.4221	0.3718
ER${}_{0.25}$	0.2326	0.2633	0.4342	0.1723	0.0354	0.2714	0.4783	0.0675	0.4232	0.3295
ER${}_{0.75}$	0.2309	0.2608	0.4331	0.1705	0.0367	0.2699	0.4768	0.0689	0.4218	0.3282
WCER	0.2416	0.2801	0.4426	0.1874	0.0320	0.2909	0.4917	0.0618	0.4355	0.3389
	n=1500									
**Estimate**	$\alpha_{1}^{\left(1\right)}$	$\alpha_{2}^{\left(1\right)}$	$\alpha_{1}^{\left(2\right)}$	$\alpha_{2}^{\left(2\right)}$	$\beta_{0}^{\left(1\right)}$	$\beta_{1}^{\left(1\right)}$	$\beta_{2}^{\left(1\right)}$	$\beta_{0}^{\left(2\right)}$	$\beta_{1}^{\left(2\right)}$	$\beta_{2}^{\left(2\right)}$
MLE	0.2479	0.2958	0.4476	0.1965	0.0306	0.2972	0.4971	0.0605	0.4468	0.3467
LS	0.2357	0.2743	0.4379	0.2131	0.0344	0.2782	0.5192	0.0653	0.4305	0.3317
QR${}_{0.25}$	0.2365	0.2747	0.4620	0.2129	0.0340	0.2784	0.5191	0.0650	0.4308	0.3678
QR${}_{0.75}$	0.2361	0.2741	0.4627	0.1867	0.0345	0.2781	0.4805	0.0654	0.4306	0.3680
ER${}_{0.25}$	0.2366	0.2752	0.4384	0.1873	0.0337	0.2786	0.4811	0.0648	0.4313	0.3331
ER${}_{0.75}$	0.2351	0.2739	0.4373	0.1862	0.0347	0.2777	0.4803	0.0656	0.4299	0.3311
WCER	0.2442	0.2899	0.4469	0.1932	0.0308	0.2930	0.4955	0.0608	0.4401	0.3433
	n=2500									
**Estimate**	$\alpha_{1}^{\left(1\right)}$	$\alpha_{2}^{\left(1\right)}$	$\alpha_{1}^{\left(2\right)}$	$\alpha_{2}^{\left(2\right)}$	$\beta_{0}^{\left(1\right)}$	$\beta_{1}^{\left(1\right)}$	$\beta_{2}^{\left(1\right)}$	$\beta_{0}^{\left(2\right)}$	$\beta_{1}^{\left(2\right)}$	$\beta_{2}^{\left(2\right)}$
MLE	0.2489	0.2978	0.4486	0.1982	0.0304	0.2985	0.4985	0.0603	0.4483	0.3482
LS	0.2418	0.2870	0.4433	0.2073	0.0322	0.2886	0.5101	0.0625	0.4401	0.3412
QR${}_{0.25}$	0.2424	0.2872	0.4569	0.2076	0.0323	0.2884	0.5103	0.0627	0.4404	0.3590
QR${}_{0.75}$	0.2416	0.2875	0.4571	0.1925	0.0324	0.2881	0.4895	0.0624	0.4402	0.3594
ER${}_{0.25}$	0.2423	0.2878	0.4432	0.1928	0.0323	0.2885	0.4896	0.0624	0.4406	0.3408
ER${}_{0.75}$	0.2415	0.2876	0.4430	0.1930	22	0.2887	0.4893	0.0622	0.4404	0.3412
WCER	0.2472	0.2951	0.4480	0.1965	0.0306	0.2967	0.4979	0.0605	0.4461	0.3473
