# Supplementary material for: The Financial Risk Measurement EVaR Based on DTARCH Models
Source: Entropy (Basel). 2023 Aug 13;25(8):1204. doi: 10.3390/e25081204 (PMC10453247; doi:10.3390/e25081204)
Supplement: Supplementary file 1 [file entropy-25-01204-s001.zip › submit-0615-supplement/supp_DTARCH_EVaR_MDPI_20230615.pdf]

entropy

MDPI

Article

Supplementary Material of The Financial Risk Measurement EVaR Based on DTARCH Models

Xiaoqian Liu [1], Zhenni Tan [1], Yuehua Wu [1,*] and Yong Zhou [2]

1 Department of Mathematics and Statistics, York University, Toronto, ON M3J 1P3, Canada; xqliu@yorku.ca (X.L.); ztan43@yorku.ca (Z.T.)
2 Key Laboratory of Advanced Theory and Application in Statistics and Data Science, MOE, and Academy of Statistics and Interdisciplinary Sciences and School of Statistics, East China Normal University, Shanghai 200062, China; yzhou@fem.ecnu.edu.cn
* Correspondence: wuyh@yorku.ca

We consider the following DTARCH$(2,2;2,2)$ model:

$$y_t = \begin{cases} \alpha_1^{(1)} y_{t-1} + \alpha_2^{(1)} y_{t-2} + \epsilon_t, & \text{if } y_{t-2} \leq 0, \\ \alpha_1^{(2)} y_{t-1} + \alpha_2^{(2)} y_{t-2} + \epsilon_t, & \text{if } y_{t-2} > 0, \end{cases}$$

where $\left(\alpha_1^{(1)}, \alpha_2^{(1)}\right) = (0.25, 0.40)$, $\left(\alpha_1^{(2)}, \alpha_2^{(2)}\right) = (0.50, 0.30)$, and $\epsilon_t = h_t u_t$, with

$$h_t = \begin{cases} \beta_0^{(1)} + \beta_1^{(1)} |\epsilon_{t-1}| + \beta_2^{(1)} |\epsilon_{t-1}|, & \text{if } y_{t-2} \leq 0, \\ \beta_0^{(2)} + \beta_1^{(2)} |\epsilon_{t-1}| + \beta_2^{(2)} |\epsilon_{t-1}|, & \text{if } y_{t-2} > 0, \end{cases}$$

where $\left(\beta_0^{(1)}, \beta_1^{(1)}, \beta_2^{(1)}\right) = (0.04, 0.20, 0.40)$, $\left(\beta_0^{(2)}, \beta_1^{(2)}, \beta_2^{(2)}\right) = (0.08, 0.35, 0.45)$.

We consider three types of innovation variables, which are distributed as $N(0,1)$, $t(6)$, and $\chi^2(4)$. They are centralized and normalized so that the medians of the absolute innovations are 1, i.e., u_t is normalized to satisfy Median$(|u_t|) = 1$. The sample size n takes the values of 100, 300, 800, 1,500 and 2,500. All the results are based on 500 Monte Carlo replications. Seven equally spaced expectiles in $(0,1)$ are chosen in each simulation setting when we apply the WCER estimation process. For QR and ER estimation, we take $\tau = 0.25$ and 0.75 respectively. In each simulation, the root mean squared error (RMSE) for different estimators are calculated, and they are reported in Tables S1–S3. And the estimated results of all estimation methods are listed in Tables S4–S6. The simulation results of this model are similar to those of the model in the text.

check for updates

Citation: Liu, X.; Tan, Z.; Wu, Y.; Zhou, Y. The Financial Risk Measurement EVaR Based on DTARCH Models. *Entropy* **2023**, *25*, 1204. https://doi.org/10.3390/e25081204

Academic Editor: Damián H. Zanette

Received: 15 June 2023
Revised: 1 August 2023
Accepted: 9 August 2023
Published: 13 August 2023

Publisher's Note: MDPI stays neutral with regard to jurisdictional claims in published maps and institutional affiliations.

Copyright: © 2023 by the authors. Licensee MDPI, Basel, Switzerland. This article is an open access article distributed under the terms and conditions of the Creative Commons Attribution (CC BY) license (https://creativecommons.org/licenses/by/4.0/).

Table S1. RMSE comparison of various estimation methods, $\epsilon_t \sim N(0,1)$

	$n = 100$									
Estimate	$\alpha_1^{(1)}$	$\alpha_2^{(1)}$	$\alpha_1^{(2)}$	$\alpha_2^{(2)}$	$\beta_0^{(1)}$	$\beta_1^{(1)}$	$\beta_2^{(1)}$	$\beta_0^{(2)}$	$\beta_1^{(2)}$	$\beta_2^{(2)}$
MLE	15.24	14.36	13.91	14.88	2.07	16.98	12.33	2.62	15.95	12.47
LS	16.92	15.71	15.03	15.24	2.49	18.44	14.93	3.06	17.07	13.76
$QR_{0.25}$	23.12	22.94	22.17	23.13	3.78	28.86	25.15	4.67	27.41	25.96
$QR_{0.75}$	23.38	22.99	22.22	23.09	3.85	28.91	25.89	4.79	27.96	26.21
$ER_{0.25}$	23.49	23.07	22.25	23.17	3.62	28.79	25.98	4.84	27.39	26.09
$ER_{0.75}$	23.65	23.01	22.29	23.11	3.67	28.83	25.79	4.76	27.84	26.12
WCER	20.11	18.99	18.74	19.75	2.61	20.14	16.22	3.20	20.83	17.39

	$n = 300$									
Estimate	$\alpha_1^{(1)}$	$\alpha_2^{(1)}$	$\alpha_1^{(2)}$	$\alpha_2^{(2)}$	$\beta_0^{(1)}$	$\beta_1^{(1)}$	$\beta_2^{(1)}$	$\beta_0^{(2)}$	$\beta_1^{(2)}$	$\beta_2^{(2)}$
MLE	10.37	9.97	9.05	10.01	1.23	12.02	8.83	1.78	11.14	9.01
LS	11.13	10.14	10.02	11.06	1.40	13.15	10.73	2.20	12.02	10.03
$QR_{0.25}$	18.34	17.86	17.11	18.09	2.65	20.21	16.99	3.56	19.14	17.44
$QR_{0.75}$	18.40	17.92	17.19	18.05	2.67	20.17	17.05	3.58	19.19	17.47
$ER_{0.25}$	18.36	17.85	17.15	18.11	2.61	20.23	17.01	3.57	19.17	17.46
$ER_{0.75}$	18.42	17.87	17.20	18.10	2.69	20.27	17.08	3.60	19.20	17.49
WCER	14.96	13.98	13.10	14.13	1.81	16.08	12.88	2.45	15.22	13.19

	$n = 800$									
Estimate	$\alpha_1^{(1)}$	$\alpha_2^{(1)}$	$\alpha_1^{(2)}$	$\alpha_2^{(2)}$	$\beta_0^{(1)}$	$\beta_1^{(1)}$	$\beta_2^{(1)}$	$\beta_0^{(2)}$	$\beta_1^{(2)}$	$\beta_2^{(2)}$
MLE	7.04	6.99	6.11	7.13	0.77	7.95	5.94	1.09	8.02	6.23
LS	7.94	7.82	6.83	7.81	0.86	8.53	6.41	1.52	8.50	6.69
$QR_{0.25}$	12.69	11.21	10.98	12.33	1.62	14.82	13.21	2.98	14.12	12.67
$QR_{0.75}$	12.94	11.17	10.89	12.45	1.65	14.80	13.36	3.03	14.23	12.71
$ER_{0.25}$	12.83	11.25	10.99	12.41	1.68	14.88	13.19	3.01	14.17	12.69
$ER_{0.75}$	12.77	11.33	11.05	12.50	1.66	14.93	13.27	3.07	14.20	12.75
WCER	8.01	7.94	7.15	8.21	0.98	8.87	6.96	2.04	9.05	7.19

	$n = 1500$									
Estimate	$\alpha_1^{(1)}$	$\alpha_2^{(1)}$	$\alpha_1^{(2)}$	$\alpha_2^{(2)}$	$\beta_0^{(1)}$	$\beta_1^{(1)}$	$\beta_2^{(1)}$	$\beta_0^{(2)}$	$\beta_1^{(2)}$	$\beta_2^{(2)}$
MLE	4.97	4.84	4.02	5.01	0.49	5.55	3.86	0.66	5.93	4.09
LS	5.56	5.43	4.61	5.65	0.57	6.17	4.48	1.02	6.50	4.66
$QR_{0.25}$	9.69	9.51	8.02	10.07	1.03	10.11	8.74	1.99	10.55	9.07
$QR_{0.75}$	9.73	9.55	8.06	10.11	1.05	10.14	8.78	2.02	10.57	9.10
$ER_{0.25}$	9.71	9.54	8.01	10.10	1.04	10.13	8.75	2.01	10.53	9.09
$ER_{0.75}$	9.75	9.58	8.05	10.15	1.07	10.12	8.77	2.03	10.59	9.13
WCER	5.78	5.65	4.99	6.03	0.66	6.29	4.64	1.21	6.87	5.10

	$n = 2500$									
Estimate	$\alpha_1^{(1)}$	$\alpha_2^{(1)}$	$\alpha_1^{(2)}$	$\alpha_2^{(2)}$	$\beta_0^{(1)}$	$\beta_1^{(1)}$	$\beta_2^{(1)}$	$\beta_0^{(2)}$	$\beta_1^{(2)}$	$\beta_2^{(2)}$
MLE	2.44	2.31	1.59	2.57	0.20	3.08	1.48	0.29	3.15	1.42
LS	2.83	2.79	2.02	3.09	0.27	3.56	1.94	0.35	3.61	1.90
$QR_{0.25}$	4.92	4.65	3.21	5.16	0.44	6.17	3.03	0.61	6.38	2.89
$QR_{0.75}$	4.95	4.67	3.25	5.20	0.46	6.20	3.06	0.62	6.43	2.92
$ER_{0.25}$	4.94	4.63	3.23	5.19	0.43	6.21	3.05	0.60	6.40	2.91
$ER_{0.75}$	4.97	4.69	3.27	5.15	0.48	6.15	3.07	0.63	6.45	2.94
WCER	3.02	2.94	2.23	3.20	0.36	3.69	2.11	0.50	3.74	2.07

Notes: All RMSEs are multiplied by 10^2.

Table S2. RMSE comparison of various estimation methods, $\epsilon_t \sim t(6)$

Estimate	$\alpha_1^{(1)}$	$\alpha_2^{(1)}$	$\alpha_1^{(2)}$	$\alpha_2^{(2)}$	$\beta_0^{(1)}$	$\beta_1^{(1)}$	$\beta_2^{(1)}$	$\beta_0^{(2)}$	$\beta_1^{(2)}$	$\beta_2^{(2)}$
$n = 100$										
MLE	11.64	11.14	10.33	9.68	1.57	16.35	13.83	1.99	13.61	11.32
LS	24.61	22.31	21.14	20.05	3.14	30.22	27.40	4.03	27.09	23.15
$QR_{0.25}$	24.69	22.49	21.20	20.44	3.19	30.41	27.52	4.07	27.35	23.29
$QR_{0.75}$	24.77	22.53	21.25	20.49	3.25	30.49	27.55	4.09	27.42	23.37
$ER_{0.25}$	24.79	22.56	21.22	20.42	3.22	30.45	27.54	4.11	27.44	23.35
$ER_{0.75}$	24.73	22.50	21.27	20.50	3.24	30.47	27.57	4.05	27.40	23.31
WCER	15.23	15.02	13.84	11.56	1.94	20.01	15.96	2.32	15.11	15.15
$n = 300$										
Estimate	$\alpha_1^{(1)}$	$\alpha_2^{(1)}$	$\alpha_1^{(2)}$	$\alpha_2^{(2)}$	$\beta_0^{(1)}$	$\beta_1^{(1)}$	$\beta_2^{(1)}$	$\beta_0^{(2)}$	$\beta_1^{(2)}$	$\beta_2^{(2)}$
MLE	8.43	8.61	7.74	8.01	1.02	10.94	8.99	1.04	9.75	7.36
LS	18.14	18.94	16.33	17.69	2.30	19.55	18.92	2.38	19.16	16.77
$QR_{0.25}$	18.26	18.96	16.40	17.72	2.37	19.59	18.97	2.39	19.24	16.80
$QR_{0.75}$	18.37	18.91	16.45	17.78	2.35	19.62	19.03	2.37	19.29	16.85
$ER_{0.25}$	18.31	18.72	16.47	17.61	2.26	19.60	18.85	2.35	19.22	16.71
$ER_{0.75}$	18.03	18.99	16.31	17.80	2.32	19.47	19.96	2.40	19.09	16.87
WCER	12.55	12.81	10.21	9.95	1.23	14.27	11.92	1.28	10.97	10.74
$n = 800$										
Estimate	$\alpha_1^{(1)}$	$\alpha_2^{(1)}$	$\alpha_1^{(2)}$	$\alpha_2^{(2)}$	$\beta_0^{(1)}$	$\beta_1^{(1)}$	$\beta_2^{(1)}$	$\beta_0^{(2)}$	$\beta_1^{(2)}$	$\beta_2^{(2)}$
MLE	6.23	6.14	5.39	5.55	0.56	7.36	6.82	0.78	4.75	3.77
LS	13.02	12.86	11.04	11.92	1.51	14.15	13.89	1.62	10.21	9.33
$QR_{0.25}$	13.11	12.90	11.09	11.97	1.56	14.20	13.94	1.64	10.25	9.37
$QR_{0.75}$	13.15	12.88	11.12	12.05	1.55	14.22	13.96	1.65	10.30	9.40
$ER_{0.25}$	13.14	12.92	11.07	12.01	1.54	14.23	13.91	1.64	10.27	9.39
$ER_{0.75}$	13.17	12.89	11.13	11.99	1.55	14.19	13.97	1.66	10.29	9.42
WCER	8.86	8.77	7.25	8.24	0.79	11.41	9.04	1.03	6.72	5.32
$n = 1500$										
Estimate	$\alpha_1^{(1)}$	$\alpha_2^{(1)}$	$\alpha_1^{(2)}$	$\alpha_2^{(2)}$	$\beta_0^{(1)}$	$\beta_1^{(1)}$	$\beta_2^{(1)}$	$\beta_0^{(2)}$	$\beta_1^{(2)}$	$\beta_2^{(2)}$
MLE	3.62	3.49	2.98	3.17	0.30	5.45	3.34	0.46	2.89	1.83
LS	9.89	9.43	8.61	8.74	1.14	10.89	9.29	1.30	8.43	7.92
$QR_{0.25}$	9.94	9.47	8.70	8.78	1.16	10.94	9.32	1.36	8.47	7.99
$QR_{0.75}$	10.01	9.49	8.68	8.84	1.19	10.98	9.37	1.35	8.50	7.96
$ER_{0.25}$	9.96	9.39	8.64	8.70	1.11	10.90	9.22	1.28	8.49	7.89
$ER_{0.75}$	9.84	9.45	8.57	8.76	1.20	10.82	9.33	1.32	8.40	7.95
WCER	4.75	4.66	3.66	4.08	0.39	7.03	4.54	0.59	3.32	2.61
$n = 2500$										
Estimate	$\alpha_1^{(1)}$	$\alpha_2^{(1)}$	$\alpha_1^{(2)}$	$\alpha_2^{(2)}$	$\beta_0^{(1)}$	$\beta_1^{(1)}$	$\beta_2^{(1)}$	$\beta_0^{(2)}$	$\beta_1^{(2)}$	$\beta_2^{(2)}$
MLE	2.15	2.09	1.52	1.98	0.13	3.61	1.78	0.20	1.39	0.96
LS	5.33	5.11	4.23	5.01	0.50	7.25	4.60	0.61	3.79	2.92
$QR_{0.25}$	5.37	5.15	4.27	5.06	0.52	7.27	4.64	0.64	3.84	2.98
$QR_{0.75}$	5.40	5.17	4.25	5.09	0.54	7.29	4.68	0.62	3.89	3.03
$ER_{0.25}$	5.39	5.19	4.24	5.05	0.51	7.30	4.66	0.63	3.87	3.05
$ER_{0.75}$	5.35	5.20	4.29	5.11	0.55	7.28	4.70	0.61	3.85	3.07
WCER	2.91	2.87	2.24	2.79	0.20	4.73	2.87	0.23	1.96	1.65

Notes: All RMSEs are multiplied by 10^2.

Table S3. RMSE comparison of various estimation methods, $\epsilon_t \sim \chi^2(4)$

					$n = 100$					
Estimate	$\alpha_1^{(1)}$	$\alpha_2^{(1)}$	$\alpha_1^{(2)}$	$\alpha_2^{(2)}$	$\beta_0^{(1)}$	$\beta_1^{(1)}$	$\beta_2^{(1)}$	$\beta_0^{(2)}$	$\beta_1^{(2)}$	$\beta_2^{(2)}$
MLE	13.26	11.49	10.92	11.12	1.71	17.84	14.59	1.96	14.39	10.88
LS	26.61	22.92	20.98	22.21	3.50	32.37	28.11	3.99	28.03	20.16
$QR_{0.25}$	26.55	22.94	21.04	22.23	3.52	32.39	28.13	4.00	28.05	20.17
$QR_{0.75}$	26.59	22.97	21.07	22.27	3.54	32.42	28.17	4.02	28.09	20.20
$ER_{0.25}$	26.51	22.89	20.94	22.17	3.48	32.33	28.07	3.96	28.01	20.14
$ER_{0.75}$	26.67	22.96	21.05	22.25	3.52	32.40	28.15	4.05	28.07	20.19
WCER	17.32	15.13	14.87	15.01	1.97	20.02	16.77	2.22	16.49	13.97
					$n = 300$					
Estimate	$\alpha_1^{(1)}$	$\alpha_2^{(1)}$	$\alpha_1^{(2)}$	$\alpha_2^{(2)}$	$\beta_0^{(1)}$	$\beta_1^{(1)}$	$\beta_2^{(1)}$	$\beta_0^{(2)}$	$\beta_1^{(2)}$	$\beta_2^{(2)}$
MLE	8.81	8.04	7.99	8.52	1.02	9.98	9.03	1.07	8.97	6.95
LS	16.79	16.31	15.82	16.54	2.13	19.55	17.67	2.31	17.04	14.99
$QR_{0.25}$	16.92	16.40	15.83	16.59	2.18	19.57	17.71	2.47	17.10	15.03
$QR_{0.75}$	16.99	16.38	15.89	16.65	2.15	19.60	17.69	2.44	17.08	15.07
$ER_{0.25}$	16.72	16.27	15.79	16.51	2.11	19.54	17.64	2.30	16.99	14.94
$ER_{0.75}$	16.90	16.39	15.87	16.60	2.17	19.63	17.75	2.40	17.07	15.05
WCER	12.15	11.98	9.02	9.25	1.25	10.78	9.93	1.34	9.89	8.06
					$n = 800$					
Estimate	$\alpha_1^{(1)}$	$\alpha_2^{(1)}$	$\alpha_1^{(2)}$	$\alpha_2^{(2)}$	$\beta_0^{(1)}$	$\beta_1^{(1)}$	$\beta_2^{(1)}$	$\beta_0^{(2)}$	$\beta_1^{(2)}$	$\beta_2^{(2)}$
MLE	6.32	6.15	5.24	6.29	0.63	7.12	6.95	0.69	5.38	4.06
LS	12.43	12.20	10.82	12.41	1.47	14.16	13.85	1.52	11.15	9.61
$QR_{0.25}$	12.47	12.25	10.87	12.49	1.49	14.20	13.89	1.54	11.19	9.68
$QR_{0.75}$	12.52	12.31	10.85	12.45	1.52	14.18	13.44	1.55	11.14	6.64
$ER_{0.25}$	12.40	12.12	10.79	12.35	1.43	14.10	13.79	1.50	11.11	9.58
$ER_{0.75}$	12.49	12.27	10.90	12.47	1.50	14.21	13.92	1.56	11.20	9.66
WCER	8.02	7.94	6.45	7.56	0.98	8.63	8.31	1.01	6.59	5.13
					$n = 1500$					
Estimate	$\alpha_1^{(1)}$	$\alpha_2^{(1)}$	$\alpha_1^{(2)}$	$\alpha_2^{(2)}$	$\beta_0^{(1)}$	$\beta_1^{(1)}$	$\beta_2^{(1)}$	$\beta_0^{(2)}$	$\beta_1^{(2)}$	$\beta_2^{(2)}$
MLE	4.20	4.02	3.11	4.13	0.34	5.06	4.98	0.41	3.25	2.17
LS	8.52	8.05	6.31	8.33	0.71	10.03	9.92	0.84	6.62	4.43
$QR_{0.25}$	8.56	8.07	6.33	8.37	0.73	10.04	9.95	0.86	6.64	4.49
$QR_{0.75}$	8.60	8.06	6.37	8.34	0.72	10.09	9.99	0.89	6.67	4.44
$ER_{0.25}$	8.48	8.01	6.30	8.30	0.70	10.01	9.90	0.82	6.60	4.41
$ER_{0.75}$	8.55	8.08	6.35	8.36	0.74	10.07	9.97	0.87	6.65	4.47
WCER	5.65	5.48	4.62	5.53	0.60	6.64	6.39	0.72	4.74	3.66
					$n = 2500$					
Estimate	$\alpha_1^{(1)}$	$\alpha_2^{(1)}$	$\alpha_1^{(2)}$	$\alpha_2^{(2)}$	$\beta_0^{(1)}$	$\beta_1^{(1)}$	$\beta_2^{(1)}$	$\beta_0^{(2)}$	$\beta_1^{(2)}$	$\beta_2^{(2)}$
MLE	2.09	1.94	1.32	2.03	0.15	2.99	2.76	0.19	2.00	1.01
LS	4.33	4.01	2.96	4.29	0.33	6.01	5.56	0.42	4.24	2.35
$QR_{0.25}$	4.37	4.03	2.98	4.31	0.35	6.07	5.60	0.44	4.27	2.39
$QR_{0.75}$	4.36	4.04	3.03	4.33	0.34	6.05	5.63	0.45	4.25	2.37
$ER_{0.25}$	4.30	3.98	2.94	4.28	0.32	5.99	5.54	0.40	4.22	2.33
$ER_{0.75}$	4.35	4.05	2.98	4.35	0.35	6.06	5.62	0.46	4.26	2.40
WCER	2.91	2.83	2.04	3.11	0.20	3.87	3.51	0.31	2.96	1.82

Notes: All RMSEs are multiplied by 10^2.

Table S4. Parameter estimate of various estimation methods, $\epsilon_t \sim N(0,1)$

	$n = 100$									
Estimate	$\alpha_1^{(1)}$	$\alpha_2^{(1)}$	$\alpha_1^{(2)}$	$\alpha_2^{(2)}$	$\beta_0^{(1)}$	$\beta_1^{(1)}$	$\beta_2^{(1)}$	$\beta_0^{(2)}$	$\beta_1^{(2)}$	$\beta_2^{(2)}$
MLE	0.2361	0.3899	0.4758	0.2865	0.0452	0.1858	0.3896	0.0849	0.3339	0.4362
LS	0.2338	0.3870	0.4736	0.2847	0.0460	0.1834	0.3875	0.0855	0.3316	0.4344
$QR_{0.25}$	0.2033	0.3560	0.4491	0.2528	0.0560	0.1512	0.3531	0.0974	0.3008	0.4034
$QR_{0.75}$	0.2032	0.3558	0.4488	0.2531	0.0562	0.1508	0.3534	0.0976	0.3003	0.4030
$ER_{0.25}$	0.2012	0.3554	0.4493	0.2523	0.0561	0.1511	0.3528	0.0977	0.3005	0.4032
$ER_{0.75}$	0.2008	0.3551	0.4481	0.2526	0.0568	0.1515	0.3526	0.0975	0.3002	0.4028
WCER	0.2167	0.3688	0.4571	0.2648	0.0519	0.1660	0.3681	0.0923	0.3145	0.4169
	$n = 300$									
Estimate	$\alpha_1^{(1)}$	$\alpha_2^{(1)}$	$\alpha_1^{(2)}$	$\alpha_2^{(2)}$	$\beta_0^{(1)}$	$\beta_1^{(1)}$	$\beta_2^{(1)}$	$\beta_0^{(2)}$	$\beta_1^{(2)}$	$\beta_2^{(2)}$
MLE	0.2410	0.3914	0.4888	0.2906	0.0436	0.1899	0.3927	0.0825	0.3388	0.4401
LS	0.2382	0.3893	0.4865	0.2881	0.0444	0.1877	0.3908	0.0830	0.3369	0.4380
$QR_{0.25}$	0.2181	0.3719	0.4658	0.2715	0.0509	0.1697	0.3729	0.0911	0.3161	0.4196
$QR_{0.75}$	0.2179	0.3715	0.4653	0.2711	0.0511	0.1691	0.3731	0.0910	0.3164	0.4193
$ER_{0.25}$	0.2178	0.3717	0.4655	0.2710	0.0512	0.1693	0.3725	0.0913	0.3163	0.4194
$ER_{0.75}$	0.2182	0.3713	0.4656	0.2714	0.0514	0.1695	0.3727	0.0914	0.3160	0.4191
WCER	0.2299	0.3828	0.4766	0.2818	0.0470	0.1801	0.3840	0.0859	0.3259	0.4304
	$n = 800$									
Estimate	$\alpha_1^{(1)}$	$\alpha_2^{(1)}$	$\alpha_1^{(2)}$	$\alpha_2^{(2)}$	$\beta_0^{(1)}$	$\beta_1^{(1)}$	$\beta_2^{(1)}$	$\beta_0^{(2)}$	$\beta_1^{(2)}$	$\beta_2^{(2)}$
MLE	0.2448	0.3954	0.4937	0.2950	0.0419	0.1943	0.3960	0.0813	0.3440	0.4441
LS	0.2439	0.3944	0.4928	0.2944	0.0423	0.1931	0.3951	0.0821	0.3432	0.4434
$QR_{0.25}$	0.2223	0.3769	0.4705	0.2752	0.0478	0.1720	0.3791	0.0871	0.3216	0.4221
$QR_{0.75}$	0.2220	0.3765	0.4709	0.2748	0.0479	0.1715	0.3789	0.0872	0.3219	0.4219
$ER_{0.25}$	0.2218	0.3763	0.4704	0.2746	0.0480	0.1718	0.3788	0.0870	0.3217	0.4223
$ER_{0.75}$	0.2214	0.3766	0.4710	0.2749	0.0476	0.1713	0.3785	0.0874	0.3215	0.4220
WCER	0.2390	0.3907	0.4875	0.2898	0.0438	0.1885	0.3918	0.0828	0.3377	0.4383
	$n = 1500$									
Estimate	$\alpha_1^{(1)}$	$\alpha_2^{(1)}$	$\alpha_1^{(2)}$	$\alpha_2^{(2)}$	$\beta_0^{(1)}$	$\beta_1^{(1)}$	$\beta_2^{(1)}$	$\beta_0^{(2)}$	$\beta_1^{(2)}$	$\beta_2^{(2)}$
MLE	0.2469	0.3971	0.4963	0.2967	0.0409	0.1966	0.3976	0.0807	0.3465	0.4470
LS	0.2458	0.3962	0.4956	0.2960	0.0414	0.1957	0.3967	0.0813	0.3457	0.4461
$QR_{0.25}$	0.2321	0.3838	0.4807	0.2829	0.0435	0.1811	0.3877	0.0852	0.3308	0.4315
$QR_{0.75}$	0.2318	0.3833	0.4809	0.2825	0.0436	0.1808	0.3873	0.0851	0.3306	0.4319
$ER_{0.25}$	0.2319	0.3835	0.4806	0.2826	0.0434	0.1810	0.3875	0.0853	0.3307	0.4317
$ER_{0.75}$	0.2315	0.3831	0.4803	0.2828	0.0437	0.1805	0.3872	0.0854	0.3303	0.4312
WCER	0.2440	0.3943	0.4925	0.2933	0.0417	0.1934	0.3951	0.0814	0.3428	0.4441
	$n = 2500$									
Estimate	$\alpha_1^{(1)}$	$\alpha_2^{(1)}$	$\alpha_1^{(2)}$	$\alpha_2^{(2)}$	$\beta_0^{(1)}$	$\beta_1^{(1)}$	$\beta_2^{(1)}$	$\beta_0^{(2)}$	$\beta_1^{(2)}$	$\beta_2^{(2)}$
MLE	0.2486	0.3985	0.4982	0.2983	0.0403	0.1983	0.3989	0.0802	0.3485	0.4487
LS	0.2481	0.3979	0.4976	0.2978	0.0405	0.1978	0.3983	0.0804	0.3479	0.4481
$QR_{0.25}$	0.2408	0.3912	0.4896	0.2910	0.0419	0.1893	0.3929	0.0824	0.3391	0.4403
$QR_{0.75}$	0.2404	0.3913	0.4895	0.2906	0.0420	0.1895	0.3926	0.0825	0.3389	0.4405
$ER_{0.25}$	0.2406	0.3911	0.4894	0.2908	0.0418	0.1891	0.3924	0.0823	0.3390	0.4401
$ER_{0.75}$	0.2405	0.3909	0.4892	0.2905	0.0421	0.1894	0.3927	0.0824	0.3387	0.4402
WCER	0.2473	0.3969	0.4965	0.2967	0.0406	0.1966	0.3977	0.0805	0.3468	0.4473

Table S5. Parameter estimate of various estimation methods, $\epsilon_t \sim t(6)$

	$n = 100$									
Estimate	$\alpha_1^{(1)}$	$\alpha_2^{(1)}$	$\alpha_1^{(2)}$	$\alpha_2^{(2)}$	$\beta_0^{(1)}$	$\beta_1^{(1)}$	$\beta_2^{(1)}$	$\beta_0^{(2)}$	$\beta_1^{(2)}$	$\beta_2^{(2)}$
MLE	0.2376	0.3882	0.4771	0.2861	0.0447	0.1844	0.3887	0.0845	0.3322	0.4358
LS	0.2121	0.3640	0.4316	0.3419	0.0601	0.2443	0.3646	0.0997	0.3017	0.4075
$QR_{0.25}$	0.2118	0.3636	0.5681	0.3423	0.0607	0.2549	0.3643	0.0998	0.4087	0.4073
$QR_{0.75}$	0.2115	0.3638	0.5685	0.2573	0.0605	0.1455	0.3641	0.1000	0.4089	0.4070
$ER_{0.25}$	0.2119	0.4363	0.4313	0.2575	0.0604	0.1453	0.4356	0.0999	0.2914	0.4071
$ER_{0.75}$	0.2884	0.3633	0.4317	0.2571	0.0606	0.1455	0.3640	0.0996	0.2912	0.4926
WCER	0.2242	0.3763	0.4438	0.2709	0.0498	0.1686	0.3772	0.0893	0.3141	0.4203
	$n = 300$									
Estimate	$\alpha_1^{(1)}$	$\alpha_2^{(1)}$	$\alpha_1^{(2)}$	$\alpha_2^{(2)}$	$\beta_0^{(1)}$	$\beta_1^{(1)}$	$\beta_2^{(1)}$	$\beta_0^{(2)}$	$\beta_1^{(2)}$	$\beta_2^{(2)}$
MLE	0.2412	0.3921	0.4839	0.2904	0.0429	0.1897	0.3925	0.0828	0.3373	0.4399
LS	0.2208	0.3716	0.4559	0.3319	0.0509	0.2328	0.3738	0.0905	0.3123	0.4186
$QR_{0.25}$	0.2202	0.3714	0.5445	0.3322	0.0511	0.2333	0.3732	0.0907	0.3880	0.4183
$QR_{0.75}$	0.2204	0.3711	0.5449	0.2671	0.0510	0.1663	0.3736	0.0909	0.3885	0.4185
$ER_{0.25}$	0.2205	0.4285	0.4557	0.2675	0.0513	0.1665	0.4265	0.0906	0.3118	0.4184
$ER_{0.75}$	0.2797	0.3710	0.4553	0.2670	0.0512	0.1662	0.3731	0.0910	0.3116	0.4820
WCER	0.2320	0.3828	0.4668	0.2793	0.0457	0.1789	0.3847	0.0855	0.3238	0.4297
	$n = 800$									
Estimate	$\alpha_1^{(1)}$	$\alpha_2^{(1)}$	$\alpha_1^{(2)}$	$\alpha_2^{(2)}$	$\beta_0^{(1)}$	$\beta_1^{(1)}$	$\beta_2^{(1)}$	$\beta_0^{(2)}$	$\beta_1^{(2)}$	$\beta_2^{(2)}$
MLE	0.2455	0.3960	0.4916	0.2951	0.0415	0.1944	0.3961	0.0813	0.3435	0.4446
LS	0.2277	0.3796	0.4704	0.3231	0.0489	0.2239	0.3799	0.0862	0.3235	0.4263
$QR_{0.25}$	0.2273	0.3791	0.5299	0.3235	0.0492	0.2242	0.3795	0.0863	0.3769	0.4261
$QR_{0.75}$	0.2275	0.3793	0.5297	0.2761	0.0490	0.1760	0.3791	0.0865	0.3767	0.4258
$ER_{0.25}$	0.2274	0.4205	0.4702	0.2763	0.0491	0.1759	0.4207	0.0866	0.3234	0.4255
$ER_{0.75}$	0.2730	0.3792	0.4700	0.2766	0.0493	0.1755	0.3794	0.0864	0.3232	0.4741
WCER	0.2404	0.3911	0.4825	0.2898	0.0434	0.1883	0.3916	0.0829	0.3359	0.4385
	$n = 1500$									
Estimate	$\alpha_1^{(1)}$	$\alpha_2^{(1)}$	$\alpha_1^{(2)}$	$\alpha_2^{(2)}$	$\beta_0^{(1)}$	$\beta_1^{(1)}$	$\beta_2^{(1)}$	$\beta_0^{(2)}$	$\beta_1^{(2)}$	$\beta_2^{(2)}$
MLE	0.2474	0.3976	0.4955	0.2970	0.0409	0.1965	0.3979	0.0807	0.3462	0.4463
LS	0.2328	0.3844	0.4809	0.3176	0.0440	0.2179	0.3847	0.0834	0.3318	0.4321
$QR_{0.25}$	0.2325	0.3840	0.5194	0.3180	0.0444	0.2184	0.3843	0.0836	0.3685	0.4318
$QR_{0.75}$	0.2323	0.3842	0.5199	0.2818	0.0443	0.1814	0.3841	0.0837	0.3689	0.4315
$ER_{0.25}$	0.2326	0.4158	0.4804	0.2822	0.0442	0.1820	0.4155	0.0835	0.3313	0.4319
$ER_{0.75}$	0.2678	0.3838	0.4802	0.2816	0.0445	0.1815	0.3840	0.0836	0.3312	0.4683
WCER	0.2443	0.3950	0.4907	0.2932	0.0419	0.1926	0.3955	0.0815	0.3421	0.4423
	$n = 2500$									
Estimate	$\alpha_1^{(1)}$	$\alpha_2^{(1)}$	$\alpha_1^{(2)}$	$\alpha_2^{(2)}$	$\beta_0^{(1)}$	$\beta_1^{(1)}$	$\beta_2^{(1)}$	$\beta_0^{(2)}$	$\beta_1^{(2)}$	$\beta_2^{(2)}$
MLE	0.2489	0.3990	0.4978	0.2986	0.0404	0.1983	0.3990	0.0803	0.3482	0.4483
LS	0.2411	0.3919	0.4903	0.3085	0.0425	0.2087	0.3922	0.0821	0.3406	0.4408
$QR_{0.25}$	0.2408	0.3917	0.5099	0.3089	0.0427	0.2090	0.3916	0.0823	0.3597	0.4405
$QR_{0.75}$	0.2405	0.3916	0.5102	0.2913	0.0426	0.1907	0.3918	0.0824	0.3598	0.4403
$ER_{0.25}$	0.2407	0.4085	0.4899	0.2912	0.0428	0.1909	0.4083	0.0822	0.3401	0.4404
$ER_{0.75}$	0.2591	0.3917	0.4902	0.2910	0.0427	0.1906	0.3915	0.0825	0.3404	0.4594
WCER	0.2479	0.3981	0.4955	0.2971	0.0407	0.1967	0.3979	0.0806	0.3462	0.4465

Table S6. Parameter estimate of various estimation methods, $\epsilon_t \sim \chi^2(4)$

Estimate	$\alpha_1^{(1)}$	$\alpha_2^{(1)}$	$\alpha_1^{(2)}$	$\alpha_2^{(2)}$	$\beta_0^{(1)}$	$\beta_1^{(1)}$	$\beta_2^{(1)}$	$\beta_0^{(2)}$	$\beta_1^{(2)}$	$\beta_2^{(2)}$
					$n = 100$					
MLE	0.2369	0.3878	0.4767	0.2855	0.0453	0.1835	0.3876	0.0848	0.3314	0.4349
LS	0.2121	0.3642	0.4418	0.3403	0.0617	0.2452	0.3637	0.1002	0.3011	0.4081
$QR_{0.25}$	0.2115	0.3638	0.5587	0.2591	0.0619	0.1543	0.3632	0.1005	0.3994	0.4078
$QR_{0.75}$	0.2111	0.3634	0.5585	0.2595	0.0622	0.1541	0.3634	0.1009	0.3997	0.4072
$ER_{0.25}$	0.2113	0.3635	0.4416	0.2594	0.0621	0.1544	0.4367	0.1006	0.3004	0.4926
$ER_{0.75}$	0.2890	0.3631	0.4412	0.2590	0.0624	0.1540	0.3631	0.1008	0.3008	0.4077
WCER	0.2239	0.3757	0.4539	0.2708	0.0505	0.1669	0.3746	0.0898	0.3123	0.4194
					$n = 300$					
Estimate	$\alpha_1^{(1)}$	$\alpha_2^{(1)}$	$\alpha_1^{(2)}$	$\alpha_2^{(2)}$	$\beta_0^{(1)}$	$\beta_1^{(1)}$	$\beta_2^{(1)}$	$\beta_0^{(2)}$	$\beta_1^{(2)}$	$\beta_2^{(2)}$
MLE	0.2406	0.3918	0.4821	0.2892	0.0432	0.1884	0.3908	0.0830	0.3365	0.4387
LS	0.2209	0.3719	0.4538	0.3322	0.0525	0.2334	0.3721	0.0922	0.3119	0.4169
$QR_{0.25}$	0.2206	0.3716	0.5466	0.2674	0.0527	0.1663	0.3718	0.0925	0.3887	0.4165
$QR_{0.75}$	0.2203	0.3714	0.5469	0.2676	0.0528	0.1665	0.3715	0.0929	0.3885	0.4163
$ER_{0.25}$	0.2205	0.3717	0.4530	0.2675	0.0528	0.1664	0.4283	0.0924	0.3117	0.4833
$ER_{0.75}$	0.2799	0.3713	0.4535	0.2671	0.0526	0.1660	0.3713	0.0926	0.3114	0.4164
WCER	0.2308	0.3823	0.4649	0.2789	0.0463	0.1770	0.3811	0.0862	0.3225	0.4278
					$n = 800$					
Estimate	$\alpha_1^{(1)}$	$\alpha_2^{(1)}$	$\alpha_1^{(2)}$	$\alpha_2^{(2)}$	$\beta_0^{(1)}$	$\beta_1^{(1)}$	$\beta_2^{(1)}$	$\beta_0^{(2)}$	$\beta_1^{(2)}$	$\beta_2^{(2)}$
MLE	0.2434	0.3938	0.4889	0.2927	0.0423	0.1920	0.3936	0.0821	0.3408	0.4424
LS	0.2269	0.3777	0.4671	0.3251	0.0497	0.2261	0.3779	0.0889	0.3213	0.4249
$QR_{0.25}$	0.2266	0.3773	0.5333	0.2746	0.0501	0.1735	0.3776	0.0891	0.3790	0.4246
$QR_{0.75}$	0.2264	0.3775	0.5337	0.2742	0.0500	0.1734	0.3774	0.0893	0.3794	0.4244
$ER_{0.25}$	0.2265	0.3774	0.4665	0.2745	0.0499	0.1736	0.4225	0.0892	0.3208	0.4758
$ER_{0.75}$	0.2733	0.3770	0.4669	0.2743	0.0502	0.1733	0.3773	0.0890	0.3211	0.4245
WCER	0.2371	0.3875	0.4780	0.2856	0.0448	0.1841	0.3875	0.0843	0.3319	0.4347
					$n = 1500$					
Estimate	$\alpha_1^{(1)}$	$\alpha_2^{(1)}$	$\alpha_1^{(2)}$	$\alpha_2^{(2)}$	$\beta_0^{(1)}$	$\beta_1^{(1)}$	$\beta_2^{(1)}$	$\beta_0^{(2)}$	$\beta_1^{(2)}$	$\beta_2^{(2)}$
MLE	0.2465	0.3968	0.4943	0.2962	0.0410	0.1958	0.3967	0.0810	0.3452	0.4460
LS	0.2335	0.3839	0.4789	0.3167	0.0440	0.2183	0.3840	0.0835	0.3299	0.4318
$QR_{0.25}$	0.2333	0.3836	0.5215	0.2831	0.0442	0.1815	0.3837	0.0837	0.3704	0.4315
$QR_{0.75}$	0.2331	0.3834	0.5217	0.2826	0.0444	0.1811	0.3834	0.0836	0.3707	0.4316
$ER_{0.25}$	0.2334	0.3835	0.4786	0.2830	0.0441	0.1814	0.4164	0.0838	0.3295	0.4686
$ER_{0.75}$	0.2670	0.3834	0.4782	0.2825	0.0445	0.1812	0.3834	0.0836	0.3294	0.4312
WCER	0.2432	0.3935	0.4895	0.2926	0.0419	0.1920	0.3937	0.0817	0.3405	0.4422
					$n = 2500$					
Estimate	$\alpha_1^{(1)}$	$\alpha_2^{(1)}$	$\alpha_1^{(2)}$	$\alpha_2^{(2)}$	$\beta_0^{(1)}$	$\beta_1^{(1)}$	$\beta_2^{(1)}$	$\beta_0^{(2)}$	$\beta_1^{(2)}$	$\beta_2^{(2)}$
MLE	0.2483	0.3985	0.4974	0.2983	0.0406	0.1978	0.3985	0.0804	0.3475	0.4479
LS	0.2412	0.3917	0.4888	0.3090	0.0422	0.2095	0.3919	0.0818	0.3396	0.4408
$QR_{0.25}$	0.2410	0.3913	0.5115	0.2908	0.0424	0.1903	0.3913	0.0819	0.3607	0.4401
$QR_{0.75}$	0.2408	0.3915	0.5118	0.2905	0.0425	0.1901	0.3914	0.0821	0.3609	0.4405
$ER_{0.25}$	0.2407	0.3916	0.4883	0.2904	0.0423	0.1902	0.4085	0.0820	0.3394	0.4597
$ER_{0.75}$	0.2591	0.3914	0.4881	0.2906	0.0427	0.1904	0.3911	0.0822	0.3392	0.4406
WCER	0.2467	0.3969	0.4946	0.2965	0.0411	0.1955	0.3968	0.0808	0.3445	0.4456
