# Supplementary figures and images for: The Financial Risk Measurement EVaR Based on DTARCH Models
Source: Entropy (Basel). 2023 Aug 13;25(8):1204. doi: 10.3390/e25081204 (PMC10453247; doi:10.3390/e25081204)

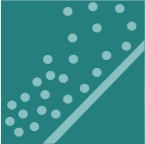

*entropy*

Supplement: Supplementary file 1 [file entropy-25-01204-s001.zip › submit-0615-supplement/Definitions/entropy-logo-eps-converted-to.pdf]

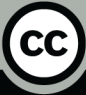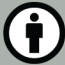

BY

Supplement: Supplementary file 1 [file entropy-25-01204-s001.zip › submit-0615-supplement/Definitions/logo-ccby-eps-converted-to.pdf]

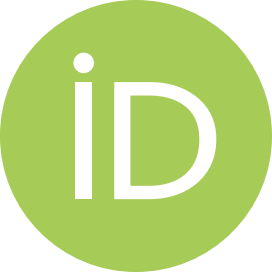

Supplement: Supplementary file 1 [file entropy-25-01204-s001.zip › submit-0615-supplement/Definitions/logo-orcid.pdf]

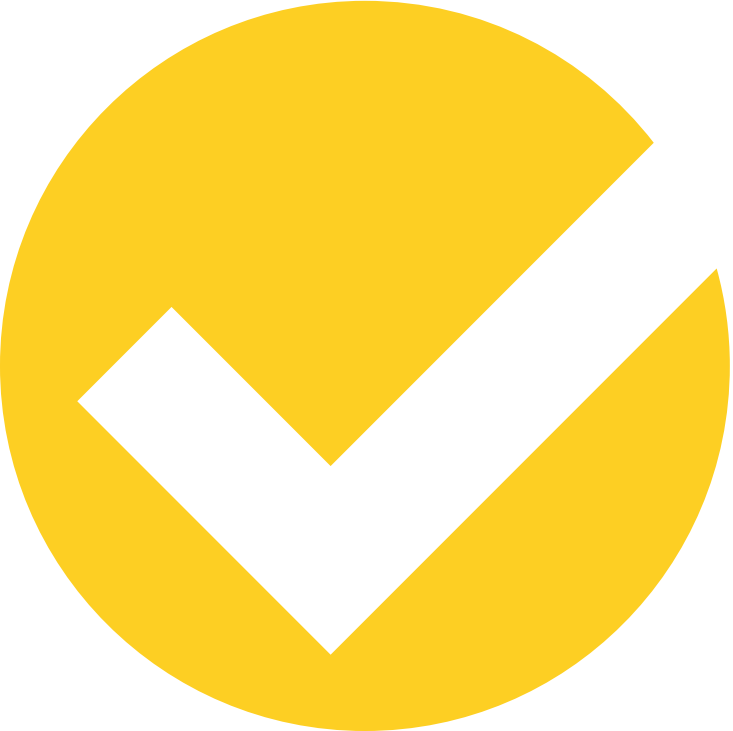

check for  
updates

Supplement: Supplementary file 1 [file entropy-25-01204-s001.zip › submit-0615-supplement/Definitions/logo-updates-eps-converted-to.pdf]

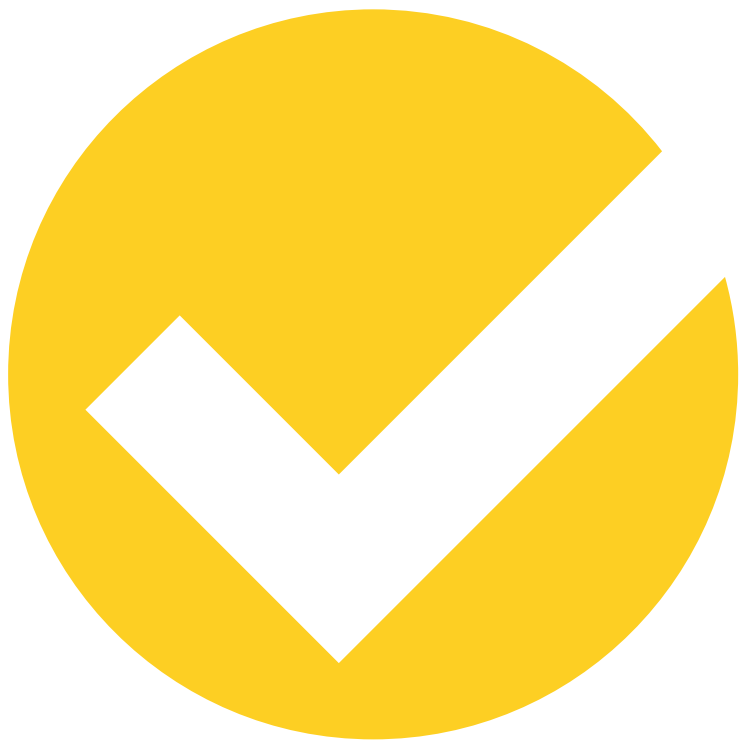

check for  
updates

Supplement: Supplementary file 1 [file entropy-25-01204-s001.zip › submit-0615-supplement/Definitions/logo-updates.pdf]
